# Lactobacillus plantarum 299v probiotic supplementation in men with stable coronary artery disease suppresses systemic inflammation

**DOI:** 10.1038/s41598-021-83252-7

**Published:** 2021-02-17

**Authors:** Benjamin C. Hofeld, Venkata K. Puppala, Sudhi Tyagi, Kwang Woo Ahn, Amberly Anger, Shuang Jia, Nita H. Salzman, Martin J. Hessner, Michael E. Widlansky

**Affiliations:** 1 Department of Medicine, Division of Cardiovascular Medicine, Medical College of Wisconsin, Milwaukee, WI USA; 2 Department of Biostatistics, Medical College of Wisconsin, Milwaukee, WI USA; 3 Department of Pediatrics, Medical College of Wisconsin, Milwaukee, WI USA; 4 Department of Microbiology and Immunology, Medical College of Wisconsin, Milwaukee, WI USA; 5 Division of Cardiovascular Medicine, Professor of Medicine and Pharmacology, Medical College of Wisconsin, Hub for Collaborative Medicine, 5th Floor A5743, 8701 W. Watertown Plank Road, Milwaukee, WI 53226 USA

**Keywords:** Cardiology, Cardiovascular biology, Cardiovascular diseases, Vascular diseases, Coronary artery disease and stable angina

## Abstract

Recent trials demonstrate that systemic anti-inflammatory therapy reduces cardiovascular events in coronary artery disease (CAD) patients. We recently demonstrated *Lactobacillus plantarum 299v* (*Lp299v)* supplementation improved vascular endothelial function in men with stable CAD. Whether this favorable effect is in part due to anti-inflammatory action remains unknown. Testing this hypothesis, we exposed plasma obtained before and after *Lp299v* supplementation from these subjects to a healthy donor’s PBMCs and measured differences in the PBMC transciptome, performed gene ontological analyses, and compared *Lp299v-*induced transcriptome changes with changes in vascular function. Daily alcohol users (DAUs) (n = 4) had a significantly different response to *Lp299v* and were separated from the main analyses. Non-DAUs- (n = 15) showed improved brachial flow-mediated dilation (FMD) and reduced circulating IL-8, IL-12, and leptin. 997 genes were significantly changed. *I.I.com* decreased (1.01 ± 0.74 vs. 0.22 ± 0.51; P < 0.0001), indicating strong anti-inflammatory effects. Pathway analyses revealed downregulation of IL-1β, interferon-stimulated pathways, and toll-like receptor signaling, and an increase in regulator T-cell (T_reg_) activity. Reductions in GBP1, JAK2, and TRAIL expression correlated with improved FMD. In non-DAU men with stable CAD, post-*Lp299v* supplementation plasma induced anti-inflammatory transcriptome changes in human PBMCs that could benefit CAD patients. Future studies should delineate changes in circulating metabolites responsible for these effects.

## Introduction

Over the last several decades, appreciation has grown for the critical role of systemic inflammation regulated by peripheral blood mononuclear cells (including lymphocytes, monocytes, and granulocytes) in the development of atherosclerotic disease and plaque vulnerability^1,2^. The mechanistic role of PBMC-driven inflammation in the development and activity of coronary artery disease has led to the completion of large randomized trials targeting pro-inflammatory cytokine and using anti-inflammatory medications that have shown some benefit in the secondary prevention of atherosclerotic events^3,4^.

Emerging data also implicate the gut microbiota in the regulation of systemic inflammation. Proinflammatory gut microbiota in murine models leads to lower levels of short chain fatty acid production, increased production of pro-inflammatory cytokines including interferon gamma (IFNα), IL-1β, and IL-2, increased PBMC production and accelerated atherosclerosis with increased neutrophil infiltration^5^. In humans, the relative abundance of certain genus of bacteria have been shown to be associated with coronary artery disease and carotid atherosclerosis^6,7^. Metagenomic analysis suggests the gut microbiomes of patients with atherosclerosis are enriched with genes that may stimulate innate immunity, with suppressed levels of genes expressing anti-inflammatory molecules including SCFAs^8^. The gut microbiota also influences gut barrier function which may influence the inflammatory impact of the gut microbiota and its metabolites^9^.

Murine studies have demonstrated worsened endothelial dysfunction accompanied by increased systemic inflammatory markers and immune cell activity in two models: conventionally raised versus germ-free ApoE^-/-^ mice, and germ-free WT mice colonized with normal gut flora versus non-colonized GF WT mice^10,11^. In an LDLR^-/-^ model, GF mice demonstrated lower carotid artery thrombus area accompanied by decreased leukocyte adhesion and decreased adhesion-dependent platelet activation ex vivo^12^. A study in angiotensin II-infused LysM transgenic mice showed reduced eNOS glutathionylation (uncoupling) and aortic endothelial superoxide formation with LysM( +) monocyte ablation^13^. Together, these murine data suggest the attractive hypothesis that targeting the gut microbiota with an intervention that leads to anti-inflammatory effect on PBMCs may also favorably impact the health of the vasculature in humans.

We recently reported that six weeks of supplementation with a probiotic, *Lactobacillus plantarum 299v (Lp299v)*, results in improved vascular endothelial function, reduced circulating levels of interleukins 8 and 12, and increased propionic acid production in men with stable coronary artery disease^14^. We hypothesized that a portion of the favorable impact of *Lp299v* is due to a systemic, genomic anti-inflammatory effect, and that a portion of these transcriptome changes would be associated with improvements in in vivo endothelial function. To test these hypotheses, we used plasma from subjects in our interventional study of *Lp299v* supplementation to determine whether post-probiotic supplementation plasma induces a systemic anti-inflammatory impact that could be favorable in men with CAD.

## Results

### Study subjects

The characteristics of the 15 subjects included in these analyses prior to and following *Lp299v* supplementation are included in Table 1. Study subjects (n = 15) were all men with an average age of 63 ± 7 years. Medical therapies included the following: aspirin (86%), beta blocker (73%), ezetimibe (6.7%), fibrate (6.7%), HMG-CoA Reductase Inhibitor (86.7%), P2Y12 inhibitor (40%), niacin (6.7%), and ranolazine (6.7%). Self-reported alcohol intake frequency were as follows: rarely/never (n = 8), several times per month (n = 1), once per week (n = 2), several times per week (n = 4). Supplemental Table S1 provides subject characteristic data on the four subjects who were excluded due to daily ETOH intake. Of note, post-supplementation, both the 15 non-daily drinking subjects and the 4 daily drinking subjects showed significantly increased circulating propionic acid levels. (Table 1 and Supplemental Table S1). There were no significant changes in butyric acid for either group. Acetic acid concentrations trended downward in both groups without statistically significant changes.

**Table 1:** Subject characteristics.

	Pre-*Lp299v*	Post-*Lp299v*	*p* Value
**Physical measurements**			
Systolic blood pressure (mmHg)	136 ± 15	137 ± 14	0.80
Diastolic blood pressure (mmHg)	75 ± 6	75 ± 8	0.84
Body mass index (kg/m^2^)	31.3 ± 4.2	31.2 ± 4.2	
**Plasma biomarkers**			
Fasting glucose (mg/dL)	112 ± 45	112 ± 50	0.91
Total cholesterol (mg/dL)	170 ± 34	164 ± 24	0.39
HDL (mg/dL)	49 ± 12	47 ± 10	0.40
LDL (md/dL)	95 ± 31	89 ± 25	0.32
Triglycerides (mg/dL)	130 ± 61	140 ± 81	0.44
Leptin (ng/mL)	136 ± 98	111 ± 90	0.002
**Short chain fatty acids**			
Propionic acid (µM)	31.9 ± 3.5	36.2 ± 3.6	0.005
Butryic acid (µM)	0.8 ± 0.4	0.8 ± 0.3	0.99
Acetic acid (µM)	42.1 ± 9.6	37.2 ± 7.4	0.16
**Vascular function measurements**			
Resting brachial diameter (mm)	4.14 ± 0.43	4.01 ± 0.39	0.14
Peak hyperermic shear (dynes/cm^2^)	74.8 ± 14.3	82.9 ± 15.1	0.58
Baseline peak shear (dynes/cm^2^)	41.4 ± 10.4	40.8 ± 12.2	0.77
Nitroglycerin-mediated dilation (%)	19.9 ± 5.7	19.2 ± 5.1	0.75

*All data presented as mean ± SD. N = 6 for Nitroglycerin-Mediated Dilation. N = 14 short chain fatty acid measurements. N = 15 for all other comparisons. *p* Values derived from Paired t-Test.

^†^*Lp299v* = *Lactobacillus plantarum 299v.*

### Measures of brachial artery flow-mediated dilation, ILs-8,12, and leptin

**Figure 1:**
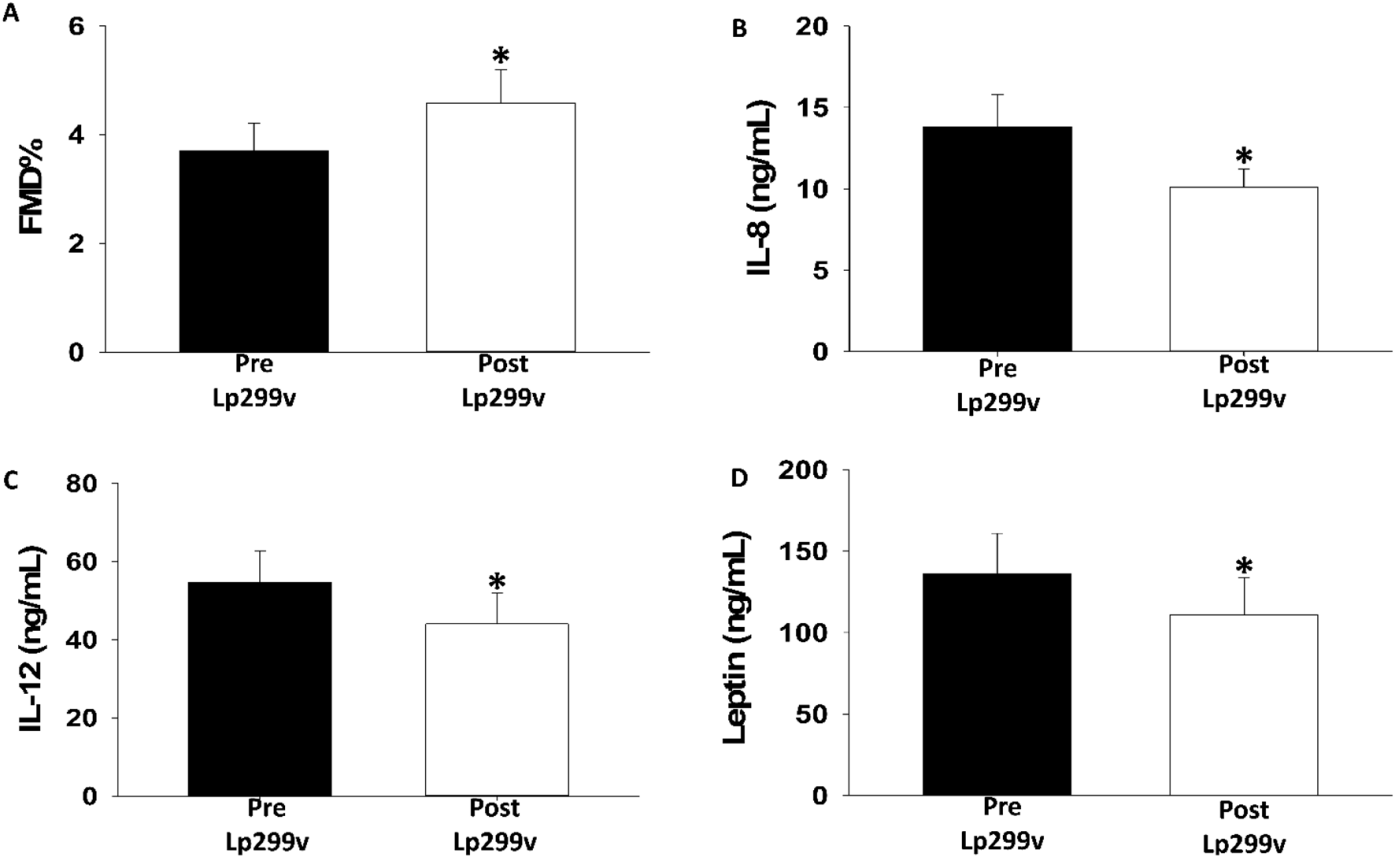
Effects of Lp299v supplementation on Brachial Artery FMD, ILs-8, 12, and Leptin in Non-Daily Alcohol Drinkers: *Lp299v* supplementation resulted in (**A**) improved brachial flow mediated dilation (FMD)% (3.70 ± 0.51 vs. 4.58 ± 0.61%, n = 15; P = 0.016) while IL-8 (**B,** 13.8 ± 2.0 vs. 10.1 ± 1.1 pg/mL, n = 15; P = 0.032), IL-12 (**C,** 54.6 ± 8.1 vs. 33.0 ± 7.9 pg/mL, n = 15; P = 0.042), and leptin (**D**, 136 ± 25 vs. 111 ± 23 ng/mL, n = 15; P = 0.002) all significantly decreased. All statistical tests paired t-tests. All values given as mean ± SE. *p < 0.05.

As seen in Fig. 1A-D, brachial artery flow-mediated dilation significantly increased (1A, 3.70 ± 0.51 vs. 4.58 ± 0.61%, P = 0.016) while IL-8 (1B, 13.8 ± 2.0 vs. 10.1 ± 1.1 pg/mL, P = 0.032), IL-12 (1C, 54.6 ± 8.1 vs. 33.0 ± 7.9 pg/mL, P = 0.042), and leptin (1D, 136 ± 25 vs. 111 ± 23, ng/mL P = 0.002) all significantly decreased. These findings confirm that this subset of subjects showed similar degrees of improvements in all these parameters as previously reported for the full study cohort.

### Plasma-induced Transcriptome changes with Lp299v supplementation

**Figure 2:**
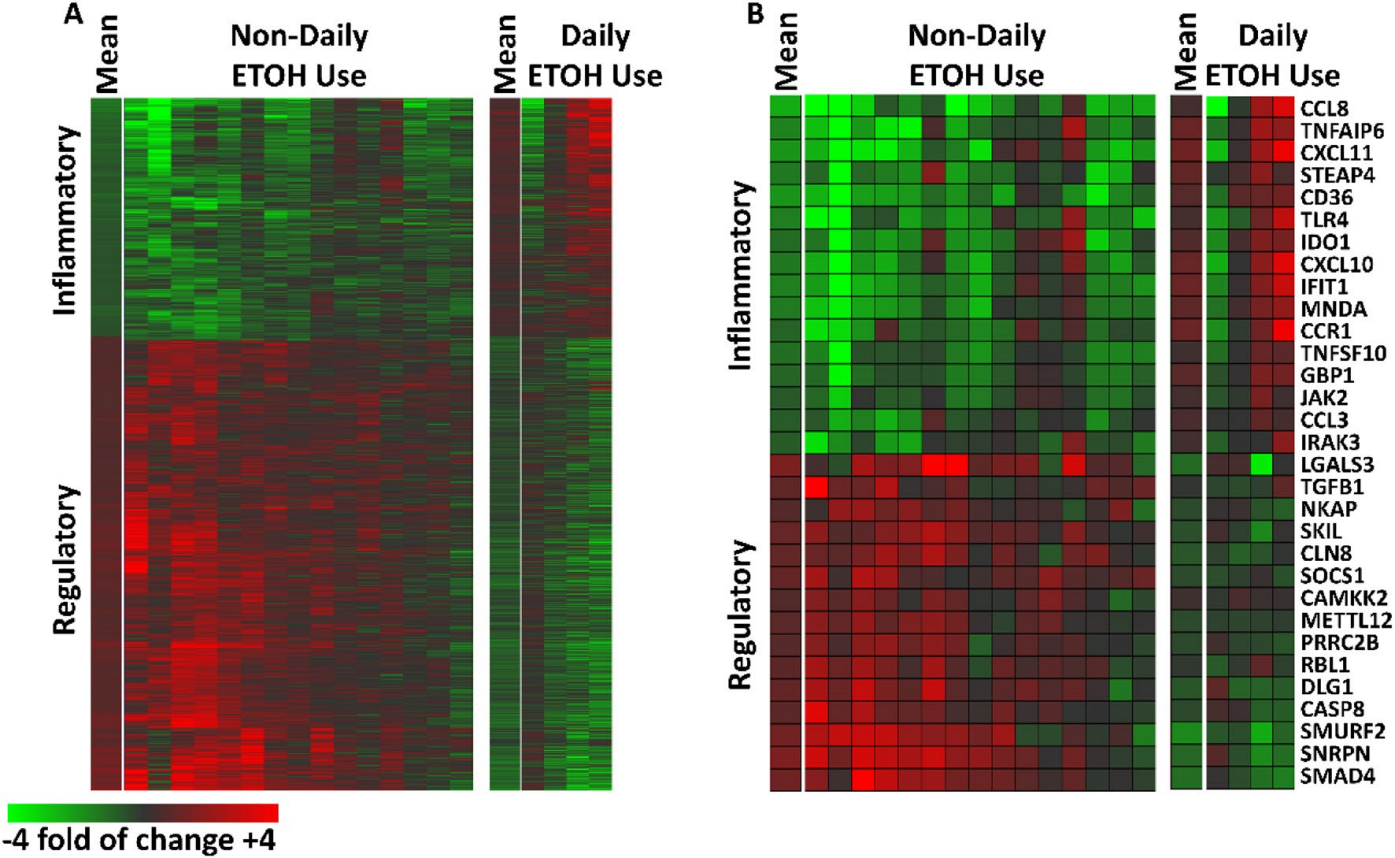
Post-Lp299v Plasma-Induced PBMC Signatures‒Stratified by Daily and Non-Daily Alcohol Drinkers: (**A**) 1,443 probe sets representing 997 unique genes were altered by at least 20% with a qval FDR < 0.2) in 15 individuals with stable CAD who did not report daily alcohol use (left) and four individuals with stable CAD who did report daily alcohol use (right). The mean for each group is indicated and each subsequent column is a subject. Data are expressed as a fold of change post- vs pre-supplementation with *Lp299v*. Red represents upregulated probe sets (n = 505); green represents downregulated probe sets (n = 938). (**B**) Well annotated transcripts associated with inflammatory and regulatory activity that showed significant change with *Lp299v* supplementation in these subjects.

Overall, 1,443 probe sets (505 downregulated and 938 upregulated) representing 997 unique genes met our criteria for differential induction after *Lp299v* supplementation; these data are depicted as heat maps (Fig. 2) where the data are expressed as a ratio of post-supplement versus pre-supplement. As seen in Fig. 2, the pattern of changes in gene transcription of the 15 non-DAU subjects differs from DAUs (Fig. 2A). In general, the 15 non-DAU subjects exhibited reduced induction of inflammatory transcripts and increased induction of regulatory transcripts after Lp299v supplementation. A subset of well-annotated transcripts representing these changes is depicted in Fig. 2B. A full list of the gene transcripts that were significantly changed are included in Supplemental Table S2.

### Ingenuity pathway analysis: regulated pathways and predicted upstream regulators

Canonical pathways considered to be significantly changed (|z-score|≥ 2 and P < 0.01) by exposure to post-*Lp299v* supplementation plasma are listed in Table 2. Additionally, IPA identified 101 upstream regulators whose suppression was suggested by the patterns in gene expression changes (Supplemental Table S3), and 25 upstream regulators that appear to be activated (Supplemental Table S4). These data suggest a significant anti-inflammatory effect of *Lp299v* supplementation, including downregulation of interferon-signaling (including IFN-γ and IL-1β), TNF-α signaling, toll-like receptor activation (TLRs 2,3,4,7, and 9), tryptophan degradation, and upregulation of IL-10 receptor subunit alpha which suppresses pro-inflammatory cytokine activation and stimulates IL-1 receptor antagonist activity. Further, upregulation of both the CD28 and iCOS signaling pathways for T helper cells along with genes regulated by suppressor of cytokine signaling-1 (SOCS1), PRDM1 (Blimp1), and DNA exonuclease 3′-repair exonuclease 1 (TREX1) suggest *Lp299v* supplementation shifts T cell activity towards suppressive immunity through regulatory T-cells (T_regs_) and suppression of innate immune activation^15–17^.

**Table 2:** Canonical pathways significantly impacted by *Lp299v* supplementation.

Canonical Pathway	*p* Value	z-score	Genes
Tryptophan degradation to 2-amino-3-carboxymuconate semialdehyde	0.00024	− 2.000	IDO1,IDO2,KMO,KYNU
Phospholipase C signaling	0.00041	2.132	ARHGEF7,CALM1,CHP1,FYN,GNB4, GNB5,HDAC2,HDAC9,LCP2,LYN,MARCK, MEF2A,MRAS,MYL12A,NAPEPLD,NFATC2, NFATC3,PLA2G4A,PPP1CB,PPP1R12A, PRKD3,RAP1A,RAP2B,RHOF,SOS2
Interferon signaling	0.00048	− 2.828	IFIT1,IFIT3,IFITM3,IFNB1,ISG15,JAK2,OAS1,SOCS1
PKCθ signaling in T lymphocytes	0.0017	2.183	ATM,CACNA1A,CD80,CD86,CHP1,FGFR1, FYN,IKBKB,LCP2,MAP3K2,MAP3K3,MRAS, NFATC2,NFATC210 ± ± ± 0,PIK3R1,RAP1A, RAP2B,SOS2
iCOS-iCOSL signaling in T helper cells	0.0030	2.309	AKT3,ATM,CALM1,CD80,CD86,CHP1,FGFR1,IKBKB,IL2RB,LCP2,NFATC2,NFATC3,PIK3R,PLEKHA2
CD28 signaling in T helper cells	0.0060	2.138	ACTR2,AKT3,ATM,CALM1,CD80,CD86,CHP1,FGFR1,FYN,IKBKB,LCP2,NFATC2,NFATC3,PIK3R1
RANK signaling in osteoclasts	0.0076	2.111	AKT3,ATM,CALM1,CHP1,FGFR1,IKBKB, MAP3K2,MAP3K3,MAPK14,MITF,NFATC2, PIK3R1
fMLP signaling in neutrophils	0.0079	2.309	ACTR2,ATM,CALM1,CHP1,FGFR1,GNB4, GNB5,MRAS,NFATC2,NFATC3,PIK3R1, PRKD3,RAP1A,RAP2B

*Lp299v = *Lactobacillus plantarum 299v* genes that were differentially expressed by at least 20% between pre- and post-*Lp299v* plasma sample exposure with an FDR < 0.2 were considered significantly changed in this exploratory pilot study. Hierarchical clustering was performed using Genesis^53^. In addition, using this set of genes, we calculated the composite inflammatory index (*I.I.*_*com*_*)*^54^*. I I.*_*com*_ was determined by calculating the ratio between the mean log intensity of the inflammatory genes (n = 505) versus the mean log intensity of the regulatory genes (n = 938) as described in Chen et al.^54^ A high score reflects greater inflammatory bias and a low score reflects greater regulatory bias. The changes in differentially expressed genes were correlated with changes in brachial FMD% using Pearson’s r with significant correlations considered for P < 0.00035 following Bonferroni correction for multiple testing. The set of genes considered to be differentially expressed were further analyzed using Ingenuity Pathway Analysis (Qiagen) and the Database for Annotation, Visualization, and Integrated Discovery (DAVID) to determine up- and down-regulated pathways and functional gene clusters as well as potential upstream regulators for the transcriptome results[55].

### DAVID functional annotation clustering and pathway analyses

DAVID 6.8 recognized 423 of the down-regulated *Homo sapien* genes transcripts in our dataset. Pathway analysis identified 7 KEGG Pathways significantly downregulated by *Lp299v* supplementation (Table 3). Consistent with our findings from the IPA upstream regulator analysis, DAVID identified downregulation of cytokine and chemokine signaling and innate immunity activation through toll-like receptors. These analyses additionally identified downregulation of pathways activated by acute infections.

**Table 3:** KEGG pathways downregulated by *Lp299v.*

Canonical pathway	*p* Value	Number of genes/fold enrichment	Genes
Toll-like receptor signaling pathway	0.000012	13/4.8	CCL3L1,CCL3L3,CCL3,CXCL10,CXCL11, CXCL9,CD80,CD86,IFNB1, LY96,TLR4,TLR7,TLR8
Cytokine-cytokine receptor interaction	0.000035	19/3.1	CCL24,CCL3L1,CCL3L3,CCL3,CCL8,CXCL10,CXCL11, CXCL9,CXCL2,TNFRSF10D, ACVR2A,INHBA,IFNB1,IL1R2,IL15,PF4V1,TNFSF10, TNFSF13B
Complement and coagulation cascades	0.000051	10/5.7	A2M,C1QB,C1QC,C3AR1,C5AR1,CFB,CFD,SERPINA1,SERPING1,TFP1
Influenza A	0.00011	15/3.4	OAS1,OAS2,OAS3,CXCL10,DDX58, TNFRSF10, CASP1,IFNB1,IFIH1,NXT2,RSAD2,TLR4,TLR7,TNFSF10
Chemokine signaling pathway	0.00023	15/3.5	CCL24,CCL3L1,CCL3L3,CCL3,CCL8,CCR1,CXCL10,CXCL11,CXCL9, CXCL2,GNB4,JAK2,LYN,ARRB1, PF4V1
Rheumatoid arthritis	0.00034	10/4.5	ATP6V1C1,CCL3L1,CCL3L3,CCL3, CD80,CD86,ANGPT1,IL15, TLR4,TNFSF13B
Measles	0.00049	12/3.6	OAS1,OAS2,OAS3, DDX58,JAK2,TNFSF10,CCNE2,IFNB1,IFIH1,TLR4,TLR7
Staphylococcus aureus infection	0.0023	7/5.1	FGCR1A,C1QB,C1QC,C3AR1,C5AR1,CFB,CFD

*Analysis performed using DAVID 6.8. Clustering Parameters: Threshold:2, EASE: 0.05. *p* Value Threshold: P < 0.005.

^†^*Lp299v* = *Lactobacillus plantarum 299v.*

DAVID 6.8 recognized 608 of the up-regulated *Homo sapien* genes transcripts in our dataset. Pathway analysis identified 8 KEGG Pathways significantly upregulated by *Lp299v* supplementation (Table 4). Consistent with our findings from the IPA upstream regulator analysis, DAVID identified upregulation of CD4 + T cell signaling pathways that regulate adaptive immunity. These analyses additionally identified two pathways involved with cell cycle checkpoints and DNA repair (FoxO and p53 signaling pathways).

**Table 4:** KEGG pathways upregulated by *Lp299v.*

Canonical pathway	*p* Value	Number of genes/fold enrichment	Genes
Chronic myeloid leukemia	0.000032	11/5.3	ABL1,AKT3,CRKL,MDM2,SMAD4,SOS2,IKBKB,PIK3R1,STAT5B, TGFB1,TGFBR2
FoxO signaling pathway	0.00010	14/3.6	AKT3,AMT,BCL2L11,FBXO32,MDM2,SKP2,SMAD4,SOS2,FOXO3,IKBKB,MAPK14,PIK3R1, TGFB1,TGFBR2
p53 signaling pathway	0.00011	10/5.2	ATM,MDM2,MDM4,CASP8,CNND3,CYCS,SERPINE1,SESN3,TSC2,ZMAT3
Neurotrophin signaling pathway	0.00015	13/3.8	ABL1,AKT3,CRKL,RAP1A,SOS2, CALM1,FOXO3,IKBKB,IRAK4, MAPK14, AP3K3,PIK3R1,ZNF274
Cell cycle	0.00077	12/3.4	ABL1,ATM,MDM2,RBL1,SKP2, SMAD4,ANAPC5,CDC14A,CCND3,TGFB1,YWHAZ
Hepatitis B	0.00084	13/3.1	AKT3,SMAD4,ATF6B,CASP8,CYCS, IKBKB,MAVS,NFATC2,NFATC3, PIK3R1,TGFB1,YWHAZ
T cell receptor signaling pathway	0.0021	10/3.5	AKT3,FYN,SOS2,DLF1,IKBKB,LCP2,MAPK14,NFATC2,NFATC3,PIK3R1
Prolactin signaling pathway	0.0039	8/3.9	HERC1,MDM2,SKP2,SMURF2, ANAPC5,CUL4A,SOCS1,UBE2G2, UBE2I,UBE2W,VHL

*Analysis performed using DAVID 6.8. Clustering Parameters: Threshold:2, EASE: 0.05. *p* Value Threshold: P < 0.005.

^†^*Lp299v* = *Lactobacillus plantarum 299v.*

### Ontology-based quantitative scoring of plasma-induced signatures

**Figure 3:**
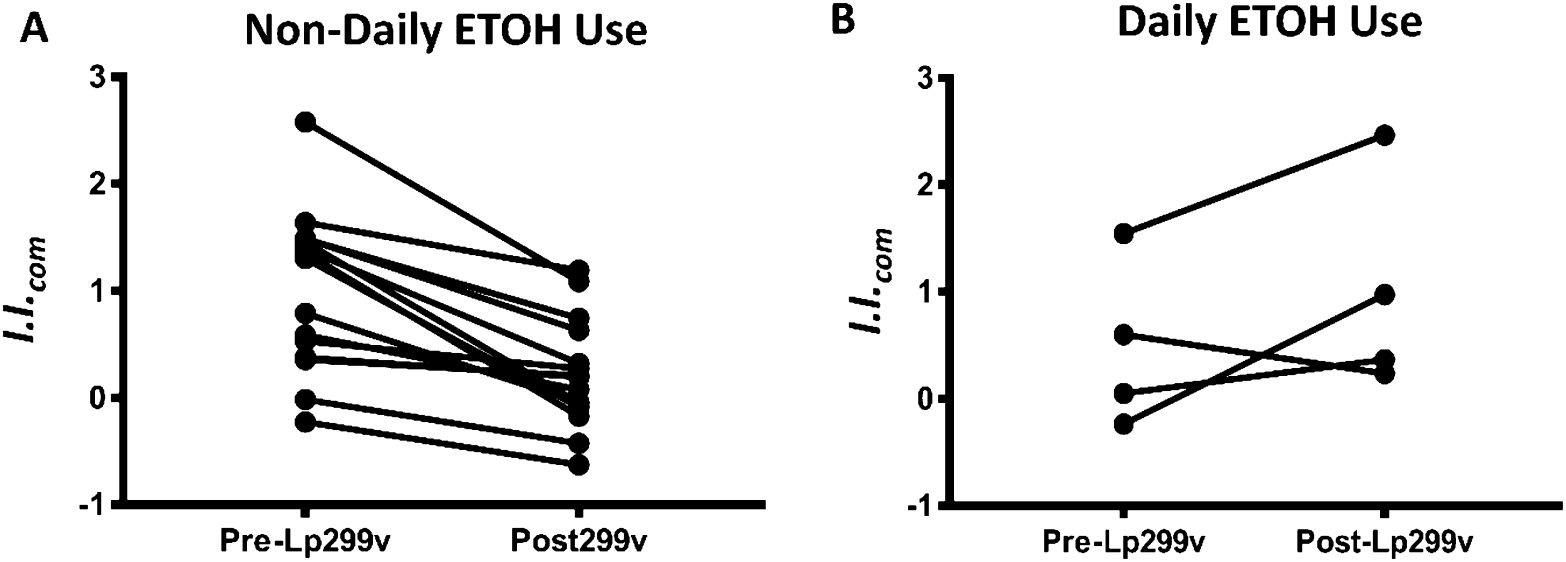
Impact of *Lp299v* supplementation on the Composite Inflammatory Index (*I.I.*_*com*_) for Non-Daily and Daily Alcohol Drinkers. **(A)** Exposure to post**-Lp299v** supplementation plasma from non-daily alcohol using subjects drove an anti-inflammatory gene expression profile in PBMCs compared to plasma obtained prior to *Lp299v* supplementations (0.22 ± 0.51 vs 1.01 ± 0.74, n = 15; P < 0.0001 by paired t-test). (**B**) *Lp299v* had no significant impact on the inflammatory gene transcription expression in subjects who reported drinking alcohol daily (0.49 ± 0.78 vs 1.01 ± 1.02, n = 4; P = 0.23 by paired t-test). All values given as mean ± SD.

As defined by the gene ontological analyses, the induced probe sets regulated by Lp299v supplementation can be broadly considered as “inflammatory” or “regulatory.” Therefore, signatures were quantitatively scored with a composite inflammatory index (*I.I.*_*com*_) determined by calculating an average ratio between the mean log intensity of the inflammatory genes versus the mean log intensity of the regulatory genes^22^. High scores reflect greater inflammatory bias and low scores reflect greater regulatory bias. The mean *I.I.*_*com*_ of the 15 CAD patients was significantly lower after *Lp299v* supplementation (1.01 ± 0.74 vs 0.22 ± 0.51 P < 0.0001, Fig. 3A) suggesting a strong anti-inflammatory influence of *Lp299v* supplementation. No such impact was seen in the four DAU subjects (0.49 ± 0.78 vs 1.01 ± 1.02, P = 0.23, Fig.e 3B). Baseline *I.I.*_*com*_ did not differ when comparing DAUs versus non-DAUs (P = 0.24).

### Correlations between changes in PBMC gene expression and brachial artery endothelial function

Of the 1373 genes differentially expressed following *Lp299v* supplementation, only three genes, guanyl binding protein 1 (GBP1), Janus kinase 2 (JAK2), and TNF Superfamily Member 10 (TNFSF10), were significantly correlated with changes in FMD% (Table 5). Transcription of all 3 genes was suppressed overall and the correlations all reflected an inverse association with changes in FMD%. Correlations between changes in gene expression and changes in FMD% for the additional genes in this set is included as Supplemental Table S5.

**Table 5:** Changes in PBMC expression associated with changes in FMD% following Lp299v supplementation.

Gene name	N36 gene symbol	Fold change	Pearson’s r	*p* Value
Guanylate binding protein 1	GBP1	− 1.48011	− 0.85	0.000044
Janus kinase 2	JAK2	− 1.27588	− 0.79	0.000069
TNF superfamily member 10	TNFSF10	− 1.51119	− 0.81	0.00020

*Changes in differentially expressed genes were correlated with changes in brachial FMD% using Pearson’s r with significant correlations considered for P < 0.00035 following Bonferroni correction for multiple testing.

## Discussion

In the absence of daily ETOH use, six weeks of supplementation with 20 billion colony-forming units of *Lp299v* in men with stable CAD resulted not only improved vascular endothelial function, but also induced changes in plasma composition to yield a strong anti-inflammatory effect. Favorable effects observed include downregulation of inflammation driven by IL-1β and TNF-α which have been successfully targeted with canakinumab (IL-1β monoclonal antibody) and colchicine to reduce secondary cardiovascular risk in large clinical trials^3,4^. In addition, *Lp299v* supplementation broadly suppressed activation of innate immunity through TLR signaling as well as favoring suppression of inflammation through upregulation of regulatory T-cells (T_regs_). Additionally, we identified suppression of transcription of three genes (GBP1, JAK2, and TNFSF10) that correlated inversely with flow-mediated dilation of the brachial artery. Whether these genes mechanistically mediate the development of inflammation-associated vascular endothelial dysfunction deserves further investigation. *Lp299v’s* favorable effects do not appear to extend to DAUs, suggesting that alcohol ingestion diminishes the anti-inflammatory effect of *Lp299v* supplementation on circulating plasma. Levels of SCFAs (acetic, propionic, and butyric acid) were similarly changed in individuals who drank daily and those who did not suggesting that these SCFAs were not mediating *Lp299v*’s anti-inflammatory effects. Taken together, our data support a paradigm of *Lp299v* contributing to improved vascular health in individuals with CAD at least in part through reductions in inflammation driven by yet-to-be identified gut-derived metabolites that should fuel future investigations.

Prior in vitro work exposing human PBMCs and *L. plantarum* species demonstrate the potential for *L. plantarum* to modulate the PBMC inflammatory response^18^. Our initial studies with *Lp299v* were stimulated by the favorable effects we observed with *Lp299v* supplementation on infarct size and reducing pro-inflammatory leptin levels in male salt-sensitive rats^19^. Different species of *L. plantarum* appear to have differing effects on the activation state and differentiation of human PBMCs in vitro^20^*.* Importantly, these data also demonstrate the in vitro effect of the direct exposure of *L. plantartum* species on PBMCs poorly predicts the in vivo response to this class of probiotics^20–22^. Our data significantly extend these prior findings by focusing on highly clinically relevant human in vivo supplementation actions with *Lp299v* and clearly delineating this supplementation changes the composition of plasma so as to exert a systemic anti-inflammatory effect that can be observed in PBMCs.

*Lp299v* supplementation in the present study had favorable anti-inflammatory effects on plasma composition including lowering of leptin, IL-12, and IL-8, and the reductions in the latter two may be, in part, leptin-mediated. Prior studies have mechanistically shown increases in serum leptin resulting in increases in ILs 12 and 8 in macrophages. Leptin enhanced secretion of LPS-stimulated IL-12 by peritoneal macrophages and stimulated IL-8 expression via p38 and ERK signaling pathways in M2 macrophages^23,24^. Given this prior work, it is quite possible that the decrease in leptin is causal in the reductions we see in IL-12 and IL-18.

Daily alcohol ingestion appears to blunt the anti-inflammatory effects of *Lp299v* in our study as demonstrated by individual inflammatory indices calculated from the transcriptome data (Fig. 3). Prior work demonstrates that alcohol ingestion promotes the growth of gram negative bacteria in the intestine and increases overall gut permeability and circulating endotoxin, triggering systemic inflammation^25,26^. Any of these established physiologic changes could account for the blunted anti-inflammatory effects of Lp299v in DAUs in our study. Our transcriptome data is consistent with these earlier findings, showing significant reductions in signaling pathways triggered by circulating lipopolysaccharide and peptidoglycan, including TLR4 activation, in our 15 non-DAU subjects (Supplemental Table S3). However, we had only four subjects who self-reported daily alcohol drinking with some variability in response, making our findings only hypothesis-generating. The dose and frequency relationships between alcohol intake and the anti-inflammatory impact of *Lp299v* supplementation, as well as the underlying mechanisms driving this mitigating effect, remain to be delineated and merit further investigation.

Over the past two decades, there has been an increasing recognition of the critical role played by activation of the innate immune system through toll-like receptors in the development of atherosclerotic disease. IPA analyses of our transcriptome data suggest *Lp299v* supplementation downregulates TLR signaling, including TLRs 2,3,4,7, and 9. Each of these has been shown to be expressed by human coronary artery and microvascular endothelial cells with activation leading to increased expression of cell adhesion molecules and pro-inflammatory cytokines^27^. In the peripheral human circulation, TLRs 2 and 4 are ubiquitously expressed and are enhanced in atherosclerotic plaques^22, 23^. TLR3 is most expressed in the carotid arteries and aorta, TLR7 in the iliac and carotid arteries, and TLR9 with overall low expression but most prominent expression in the iliac arteries^28^ TLR 2,3,4,7, and 9 stimulation have been implicated in the development of atherosclerosis through combination of promoting of lipid uptake into the plaques, monocyte activation, endothelial dysfunction, and foam cell formation^29–34^. Recently TLR2 has been shown to facilitate microbiota-induced VWF expression and systemic interferon responses mice^35,36^. Our data suggest *Lp299v* may reduce inflammation in part through reducing TLR activation in humans. Additional work will be necessary to better determine how *Lp299v* interacts with the innate immune system to suppress inflammation and atherogenesis.

Functional analyses of our transcriptome data support a paradigm of *Lp299v* supplementation increasing the number and activity of regulatory T-cells (T_regs_). T_regs_ generally exert an anti-inflammatory influence and suppress self-antigen recognition. In mouse models of hypertension and atherosclerosis, T_regs_ reduce monocyte activation and infiltration into the vasculature and atherosclerosis formation, preserve endothelium-dependent vasodilation of coronary arterioles in mice through increased IL-10 expression, reduced IL-1β and TNF-α expression leading to suppression of endothelial expression of adhesion molecules like VCAM-1 and E-selectins^37,38^. Our data reporting that *Lp299v*-induces reductions in IL-1β and TNF signaling as well as upregulation of IL-10 receptor signaling in mononuclear cells are consistent with these mouse data and suggest an association between anti-inflammatory effect of T_reg_ upregulation and improvements in endothelial function extend to humans with CAD. Additional work to delineate the mechanistic connections in human are warranted, particularly in light of the efficacy of IL-1β inhibition in reducing cardiovascular risk^3^.

We identified three genes (JAK2, GBP1, and TNFSF10) whose transcription was significantly reduced in PBMCs by post-*Lp299v* supplementation plasma. These changes inversely correlated with increases in brachial FMD%. JAK2, a tyrosine kinase involved in cytokine signaling, increases plaque and plaque necrotic core size in hemopoietic cells and increases IL-1β from mononuclear cells challenged with lipopolysaccharide, when activated; it’s suppression increases expression and activity of anti-inflammatory T_regs_^39,40^. Here, the reduction in JAK2 transcription appears consistent with the increases in expression of canonical pathways involved with increased T_reg_ pathways (Table 2) and as well as the decrease in expression in genes regulated by IL-1β signaling detected by our Ingenuity Pathway Analysis for upstream regulators (Supplemental Table S3). GBP1 is an IFNγ-induced GTPase found in T-cells, B-cells, and endothelial cells whose activation in the innate immune response to pathogens results in an inflammatory activation of endothelial cells and inhibited angiogenesis^35–38^. TNFSF10 (TRAIL) is an apoptosis-inducing ligand expressed in T cells and induced by IFNα and β to enhance T-cell cytotoxicity^41^. It has been detected in CD3 + T-cells of human atherosclerotic plaques with higher concentrations in vulnerable plaques^42^. Further studies determining the impact of *Lp299v* on endothelial cell expression of all three genes and their concomitant impact on interactions between PBMCs and endothelium, and endothelial function, are warranted.

Our study has some limitations. This study included a small number of only male subjects; whether these findings generalize to women and hold for a large group of male subjects await further elucidation which we will be pursing though a randomized control trial (NCT03267758). We did not perform transcriptome analyses on PBMCs directly from study subjects prior to and following *Lp299v* supplementation given concerns for confounding by differences in the types and amounts of pharmacological therapies. However, our approach more clearly delineates that *Lp299v’s* anti-inflammatory effects are mediated by factors in the circulating plasma. We did not examine the direct impact of *Lp299v* on cultured endothelial cells which may reveal a direct impact of *Lp299v* supplementation on the activation state of the endothelium itself. Nevertheless, PBMCs play a critical role in the development of coronary atherosclerosis and the stability of atherosclerotic plaques making our findings highly relevant to vascular disease. Balanced against these limitations is the novelty of the findings suggesting the anti-inflammatory effect of supplementation with probiotic *Lp299v* may account for part of its favorable vascular effects.

## Conclusions

In this pilot study of 15 men with stable CAD, we demonstrated that six weeks of *Lp299v* supplementation not only improved vascular endothelial function, but also had a significant anti-inflammatory effect. This anti-inflammatory effect appears to be at least in part due to suppression of innate immune signaling and increased activity of T_regs_. We additionally identified three genes (JAK2, GBP1, and TNFSF10) whose mRNA transcription suppression by *Lp299v* in PBMCs was inversely correlated with brachial FMD% suggesting a potential more direct role for these proteins in inflammation-induced endothelial dysfunction in humans. The changes in circulating metabolites induced by *Lp299v* responsible for these favorable effects remain to elucidate and hold promise for further delineating the mechanisms behind improvements in vascular health with this probiotic intervention.

## Methods

### Subjects and study protocol

The study protocol was reviewed and approved by the Institutional Research Board at the Medical College of Wisconsin, and all experiments were performed in accordance with their regulations and guidelines. Informed consent was given by all participants within this study. 23 men with stable CAD (ages 40–75) were recruited as previously described^14^. This study includes analyses on 15 of the 23 subjects that were enrolled on the initial report on the impact of *Lp299v* supplementation on endothelial function in men with stable CAD^14^. As noted in the initial report, two subjects enrolled were disqualified because they failed to pass the initial screening visit. One subject had a stroke while on protocol and did not complete the study. We excluded an additional four subjects from these study analyses who were self-reported daily alcohol users (DAUs), but did perform transcriptome analyses using their plasma to validate their exclusion. One subject did not have plasma samples from all study visits and we were therefore unable to perform transcriptome analyses for this subject. After exclusions, a total of 15 subjects were available for this set of analyses. The inclusion and exclusion criteria are identical to the previously described study protocol^8^. Individuals with left-ventricular ejection fraction less than 45% within one year of enrollment, uncontrolled hypertension (blood pressure > 170/110 mmHg), a creatinine clearance less than 60 mL/min, recent antibiotic or probiotic administration (within 12 weeks of enrollment), chronic liver disease, cancer requiring chemotherapy within five years of enrollment, or changes in vasoactive medications (within six weeks of enrollment) were excluded.

All subjects underwent focused medical history reviews and physical exams to screen out occult disease or instability of cardiac status that would exclude a subject. In addition, each subject was asked to self-report their daily alcohol intake in one of five categories: rarely/never, once per month, several times per month, once per week, several times per week, and daily. Subjects who passed screening and enrolled in the study underwent an initial set of measurements (heart rate and blood pressure in triplicate; height and waist circumference; IL-8, IL-12, leptin, and glycosylated hemoglobin from peripheral upper extremity venous blood, which was saved for the plasma induced transcriptome studies; and endothelial function by vascular artery ultrasound). Subjects were subsequently placed on once daily supplementation with 80 ml (2.7 oz.) of GoodBelly Straight (Probi, Sweden). Each daily supplement contained 20 billion colony forming units of *L.plantarum 299v*. Subjects returned to the study center approximately six weeks following beginning supplementation and repeated the studies from their initial study visit.

### Measurement of endothelial function by vascular ultrasound

Measurements of vascular endothelial function were performed using high-resolution vascular ultrasound as previously described^14,43–48^. All image acquisition and analyses were performed by trained technicians. Our measurements are highly reproducible between different operators (average intra-class correlation between four technicians for ten studies of 0.97 [0.92–0.99, P < 0.001] and a single rater intraclass correlation coefficient of 0.88 [0.74–0.97, P < 0.001].

### Measurement of circulating IL-8, IL-12, and leptin plasma levels

Serum cytokines and leptin levels were measured as described previously^14^. Sample collections were taken from all subjects between the hours of 08:00 AM and 12:00 PM on the day of study visit. IL-8 and IL-12 measurements were performed by Eve Technologies (Calgary, Canada). Human plasma leptin was measured using a quantitative ELISA kit (R&D Systems, Minneapolis, MN, #DLP00) on EDTA-preserved plasma both pre- and post-probiotic treatment; assays were read with a SpectraMax M5e Microplate Reader (Molecular Devices, Sunnyvale, CA).

### Plasma-induced regulation of mononuclear cell transcription

Using a validated assay^49^, we incubated 200 µL of pre- and post-Lp299v supplementation plasma from each subject in separate wells of Costar 24-well plates seeded with 5 × 10^5^ UPN119 PBMCs (Cellular Technology LTD, Shaker Heights, OH). Wells also contained RPMI 1,640 medium supplemented with 100 U/ml penicillin and 100 µg/mL streptomycin to a total volume of 500 µL. RNA from PBMCs was subsequently isolated, labeled, and hybridized to the Affymetrix U133 + 2.0 array as previously described^50,51^. Affymetrix microarray data were uploaded to NCMI Gene Expression Omnibus (GEO, http://www.ncbi.nlm.nih.gov/geo/).

### Statistical analyses

All data analyses were performed using SPSS 24.0, SigmaPlot 12.5, and GraphPad Prism. Subject characteristics prior to and following *Lp299v* supplementation were compared by paired t-test, Wilcoxon signed-rank test, or chi-square tests as appropriate. Changes in brachial artery parameters and inflammatory cytokines were compared by paired t-test or the signed-rank test as appropriate. For these data, P < 0.05 was considered significant. For the plasma-induced transcriptome data, genes that were differentially expressed by at least 20% between pre- and post-*Lp299v* plasma sample exposure with an FDR < 0.2 were considered significantly changed in this exploratory pilot study. Hierarchical clustering was performed using Genesis^52^. In addition, using this set of genes, we calculated the composite inflammatory index (*I.I.*_*com*_*)*^53^. *I I.*_*com*_ was determined by calculating the ratio between the mean log intensity of the inflammatory genes (n = 505) versus the mean log intensity of the regulatory genes (n = 938) as described in Chen et al.^53^ A high score reflects greater inflammatory bias and a low score reflects greater regulatory bias. The changes in differentially expressed genes were correlated with changes in brachial FMD% using Pearson’s r with significant correlations considered for P < 0.00035 following Bonferroni correction for multiple testing. The set of genes considered to be differentially expressed were further analyzed using Ingenuity Pathway Analysis (Qiagen) and the Database for Annotation, Visualization, and Integrated Discovery (DAVID) to determine up- and down-regulated pathways and functional gene clusters as well as potential upstream regulators for the transcriptome results^54^.

## Supplementary Information

Supplementary Information

## Data Availability

Transcriptome microarray data is publicly available in the Gene Expression Omnibus (GEO) repository (accession number GSE156357). All other data analyzed for this study can be found within this published manuscript and the supplementary materials.

## Abbreviations

*I.I.com*: Composite inflammatory index
CAD: Coronary artery disease
SOCS1: Cytokine signaling-1
DAVID: Database for annotation, visualization, and integrated discovery
DAU: Daily alcohol user
TREX1: DNA exonuclease 3′-repair exonuclease 1
FMD: Flow-mediated dilation
GBP1: Guanyl binding protein 1
IFNα: Interferon gamma
JAK2: Janus kinase 2
Lp299v: Lactobacillus plantarum 299v
PBMCs: Peripheral blood mononuclear cells
Blimp1: PRDM1
T_reg_: Regulator T-cell
TNFSF10: TNF Superfamily Member 10
TRAIL: TNFSF10
