# Supplementary material for: Lactobacillus plantarum 299v probiotic supplementation in men with stable coronary artery disease suppresses systemic inflammation
Source: Sci Rep. 2021 Feb 17;11:3972. doi: 10.1038/s41598-021-83252-7 (PMC7889883; doi:10.1038/s41598-021-83252-7)
Supplement: Supplementary file 1 — Supplementary Information [file 41598_2021_83252_MOESM1_ESM.docx]

**SUPPLEMENTAL MATERIAL:**

*Lactobacillus plantarum 299v Probiotic Supplementation in Men with Stable Coronary Artery Disease Suppresses Systemic Inflammation*

Benjamin C. Hofeld, MD, Venkata K. Puppala, MD, Sudhi Tyagi, MD, Kwang Woo Ahn, PhD, Amberly Anger, BS, Shuang Jia, MS, Nita H. Salzman, MD, PhD, Martin J. Hessner, PhD, and Michael E. Widlansky, MD, MPH.

List of Supplemental Tables

Supplemental Table S1: Subject Characteristics for the Four Subjects Reporting Daily Alcohol Use

Supplemental Table S2: 1443 probe sets with Significant Changes in Gene Expression with *Lp299v* Supplementation

Supplemental Table S3: Ingenuity Pathway Analysis List of Upstream Regulators Predicted to be Inhibited by Lp299v *Supplementation*

Supplemental Table S4: Ingenuity Pathway Analysis List of Upstream Regulators Predicted to be Activated by Lp299v *Supplementation*

Supplemental Table S5: Correlations Between Changes in Gene Expression and Changes in Brachial Artery Flow- Mediated Dilation

Supplemental Table S1 – Subject Characteristics for the Four Subjects Reporting Daily Alcohol Use

|  | **Pre-*Lp299v*** | **Post-*Lp299v*** | **P-Value** |
| --- | --- | --- | --- |
| **Physical Measurements** |  |  |  |
| Systolic Blood Pressure | 127±2 | 130±7 | 0.61 |
| Diastolic Blood Pressure | 724±6 | 73±6 | 0.72 |
| Body Mass Index (kg/m^2^) | 31.0±4.7 | 31.4±48 | 0.15 |
| **Plasma Biomarkers** |  |  |  |
| Fasting Glucose (mg/dL) | 94±7 | 102±11 | 0.36 |
| Total Cholesterol (mg/dL) | 179±57 | 168±61 | 0.18 |
| HDL (mg/dL) | 58±18 | 55±23 | 0.32 |
| LDL (md/dL) | 101±46 | 94±50 | 0.22 |
| Triglycerides (mg/dL) | 96±16 | 101±31 | 0.78 |
| Leptin (ng/mL) | 98±56 | 73±40 | 0.23 |
| **Short Chain Fatty Acids** |  |  |  |
| Propionic acid (µM) | 29.0±1.4 | 35.9±2.9 | 0.003 |
| Butryic acid (µM) | 0.9±0.1 | 0.9±0.3 | 0.71 |
| Acetic acid (µM) | 43.7±5.3 | 36.7±4.3 | 0.19 |
| **Vascular Function Measurements** |  |  |  |
| Resting Brachial Diameter (mm) | 3.93±0.14 | 4.05±0.64 | 0.69 |
| Peak Hyperermic Shear (dynes/cm^2^) | 72.9±9.6 | 70.1±16.7 | 0.69 |
| Baseline Peak Shear (dynes/cm^2^) | 39.1±9.4 | 37.6±9.4 | 0.77 |
| Nitroglycerin-Mediated Dilation (%) | 24.0±8.6 | 24.9±10.7 | 0.72 |

All data presented as mean±SD. N=4 or all comparisons. Average age of subjects: 68±5 years. Two subjects took aspirin, one took ezetimibe, one took a beta blocker, one took a fibrate, 2 took a P2Y12 inhibitor,

Supplemental Table S2: 1443 probe sets with Significant Changes in Gene Expression with *Lp299v* Supplementation

| **N36 Gene Symbol** | **Chromosomal Location** | **Full Gene Name** | **Post Lp299v RMS** | **Pre Lp299v RMS** | **Log2R Post vsPre rmS1&**  **5-8-10** | **Fold- Change Post vsPre rmS1&**  **5-8-10** | **PairedTp Post vsPre rmS1& 5-**  **8-10** | **PairedT QvalFDR Post vsPre rmS1& 5-**  **8-10** |
| --- | --- | --- | --- | --- | --- | --- | --- | --- |
| CCL8 | chr17q11.2 | chemokine (C-C motif) ligand 8 | 6.40925 | 7.60959 | -1.20034 | -2.29794 | 0.0007437 | 0.128628 |
| IL1R2 | chr2q12 | interleukin 1 receptor, type II | 3.86271 | 5.08389 | -1.22117 | -2.33136 | 0.0034133 | 0.150538 |
| IL1R2 | chr2q12 | interleukin 1 receptor, type II | 3.5693 | 4.36299 | -0.793689 | -1.7335 | 0.0165108 | 0.196619 |
| CXCL11 | chr4q21.2 | chemokine (C-X-C motif) ligand 11 | 5.94992 | 6.91122 | -0.961303 | -1.94707 | 0.001614 | 0.145076 |
| TNFAIP6 | chr2q23.3 | tumor necrosis factor, alpha-induced  protein 6 | 4.46027 | 5.25103 | -0.790756 | -1.72998 | 0.0083762 | 0.170128 |
| CD36 | chr7q11.2 | CD36 molecule (thrombospondin  receptor) | 5.38269 | 6.34325 | -0.960562 | -1.94607 | 0.0001359 | 0.111326 |
| STEAP4 | chr7q21.12 | STEAP family member 4 | 4.10251 | 4.79406 | -0.691548 | -1.61502 | 0.0100017 | 0.175253 |
| IDO1 | chr8p12-p11 | indoleamine 2,3-dioxygenase 1 | 8.62174 | 9.18044 | -0.5587 | -1.47294 | 0.0175222 | 0.198559 |
| MNDA | chr1q22 | myeloid cell nuclear differentiation  antigen | 7.29626 | 7.99714 | -0.700886 | -1.6255 | 0.0004106 | 0.112085 |
| CXCL11 | chr4q21.2 | chemokine (C-X-C motif) ligand 11 | 7.13552 | 7.89373 | -0.75821 | -1.69139 | 0.003573 | 0.150674 |
| IFIT1 | chr10q23.31 | interferon-induced protein with  tetratricopeptide repeats 1 | 9.24388 | 9.99846 | -0.754581 | -1.68714 | 0.0009694 | 0.129126 |
| CXCL10 | chr4q21 | chemokine (C-X-C motif) ligand 10 | 8.46096 | 9.14756 | -0.686604 | -1.60949 | 0.0038064 | 0.150735 |
| RGL1 | chr1q25.3 | ral guanine nucleotide dissociation  stimulator-like 1 | 7.67971 | 8.41464 | -0.734926 | -1.66431 | 0.001684 | 0.145076 |
| PLA2G4A | chr1q25 | phospholipase A2, group IVA  (cytosolic, calcium-dependent) | 5.75918 | 6.47614 | -0.716965 | -1.64372 | 0.0027111 | 0.148407 |
| CD36 | chr7q11.2 | CD36 molecule (thrombospondin  receptor) | 6.42766 | 7.17324 | -0.745581 | -1.67665 | 0.0008357 | 0.128628 |
| CD36 | chr7q11.2 | CD36 molecule (thrombospondin  receptor) | 8.377 | 9.05625 | -0.67926 | -1.60132 | 0.0003695 | 0.111326 |
| CD36 | chr7q11.2 | CD36 molecule (thrombospondin  receptor) | 5.6322 | 6.23244 | -0.600242 | -1.51597 | 0.0003434 | 0.111326 |
| FGL2 | chr7q11.23 | fibrinogen-like 2 | 8.32574 | 8.89988 | -0.574143 | -1.48879 | 0.0011781 | 0.134826 |
| FGL2 | chr7q11.23 | fibrinogen-like 2 | 9.34462 | 9.89232 | -0.547694 | -1.46175 | 0.0022886 | 0.148407 |
| SERPINB2 | chr18q21.3 | serpin peptidase inhibitor, clade B  (ovalbumin), member 2 | 7.70862 | 8.54942 | -0.840802 | -1.79105 | 0.0016865 | 0.145076 |
| TLR4 | chr9q33.1 | toll-like receptor 4 | 4.18195 | 4.93194 | -0.749991 | -1.68178 | 0.0025405 | 0.148407 |
| IL1RN | chr2q14.2 | interleukin 1 receptor antagonist | 6.26469 | 6.93601 | -0.67132 | -1.59253 | 0.0143014 | 0.191711 |
| EMP1 | chr12p12.3 | epithelial membrane protein 1 | 6.27291 | 6.9146 | -0.641691 | -1.56016 | 0.0095128 | 0.173123 |
| EMP1 | chr12p12.3 | epithelial membrane protein 1 | 7.96556 | 8.58146 | -0.615903 | -1.53252 | 0.0097881 | 0.174094 |
| SCIN | chr7p21.3 | scinderin | 4.56601 | 5.26523 | -0.69922 | -1.62363 | 0.0035651 | 0.150674 |
| SERPING1 | chr11q12.1 | serpin peptidase inhibitor, clade G (C1  inhibitor), member 1 | 5.01491 | 5.62887 | -0.613963 | -1.53046 | 0.0029643 | 0.148407 |
| ARMC9 | chr2q37.1 | armadillo repeat containing 9 | 5.40095 | 6.01152 | -0.610568 | -1.52686 | 0.0016503 | 0.145076 |
| RIN2 | chr20p11.22 | Ras and Rab interactor 2 | 7.862 | 8.49576 | -0.633761 | -1.55161 | 0.0063898 | 0.163699 |
| RSAD2 | chr2p25.2 | radical S-adenosyl methionine domain  containing 2 | 8.99101 | 9.54249 | -0.551481 | -1.46559 | 0.0137201 | 0.190636 |
| NFXL1 | chr4p12 | nuclear transcription factor, X-box  binding-like 1 | 7.52453 | 8.19511 | -0.67058 | -1.59171 | 0.0004865 | 0.120703 |
| GBP1 | chr1p22.2 | guanylate binding protein 1, interferon-  inducible | 8.51156 | 9.05593 | -0.544362 | -1.45838 | 0.0028506 | 0.148407 |
| TNFSF10 | chr3q26 | tumor necrosis factor (ligand)  superfamily, member 10 | 8.58652 | 9.1822 | -0.595681 | -1.51119 | 0.0017906 | 0.145076 |
| TNFSF10 | chr3q26 | tumor necrosis factor (ligand)  superfamily, member 10 | 8.38199 | 8.94118 | -0.559195 | -1.47345 | 0.0047829 | 0.155863 |

| IFIT2 | chr10q23.31 | interferon-induced protein with  tetratricopeptide repeats 2 | 9.19936 | 9.74755 | -0.548195 | -1.46225 | 0.0031497 | 0.148505 |
| --- | --- | --- | --- | --- | --- | --- | --- | --- |
| CMPK2 | chr2p25.2 | cytidine monophosphate (UMP-CMP)  kinase 2, mitochondrial | 9.22536 | 9.76837 | -0.543009 | -1.45701 | 0.0035344 | 0.150674 |
| TNFSF10 | chr3q26 | tumor necrosis factor (ligand)  superfamily, member 10 | 6.82145 | 7.44509 | -0.62364 | -1.54076 | 0.0032622 | 0.149592 |
| SLC5A3 | chr21q22.12 | solute carrier family 5 (sodium/myo- inositol cotransporter), member 3 | 5.66176 | 6.42745 | -0.765692 | -1.70019 | 0.0034373 | 0.150538 |
| SLC5A3 | chr21q22.12 | solute carrier family 5 (sodium/myo- inositol cotransporter), member 3 | 9.83332 | 10.5143 | -0.680989 | -1.60324 | 0.0035971 | 0.150674 |
| SLC5A3 | chr21q22.12 | solute carrier family 5 (sodium/myo- inositol cotransporter), member 3 | 10.3211 | 10.8935 | -0.572421 | -1.48702 | 0.0149508 | 0.192697 |
| MRPS6 | chr21q22.11 | mitochondrial ribosomal protein S6 | 9.43331 | 9.92805 | -0.494737 | -1.40906 | 0.0100523 | 0.175825 |
| FCGR1B | chr1p11.2 | Fc fragment of IgG, high affinity Ib,  receptor (CD64) | 4.78766 | 5.47504 | -0.687378 | -1.61035 | 0.0008815 | 0.128628 |
| SORT1 | chr1p13.3\|1p21.  3-p13.1 | sortilin 1 | 4.34628 | 4.95815 | -0.611864 | -1.52823 | 0.0034454 | 0.150538 |
| SLC12A8 | chr3q21.2 | solute carrier family 12, member 8 | 3.80151 | 4.30579 | -0.504277 | -1.41841 | 0.0073019 | 0.167501 |
| FCGR1A /// FCGR1C | chr1q21.2 /// chr1q21.2-q21.3 | Fc fragment of IgG, high affinity Ia,  receptor (CD64) /// Fc fragment of IgG, high affinity Ic, receptor (CD64), pseudogene | 4.97888 | 5.44901 | -0.470137 | -1.38524 | 0.01026 | 0.177837 |
| LINC00158 | chr21q21.3 | long intergenic non-protein coding  RNA 158 | 2.81065 | 3.39573 | -0.58508 | -1.50012 | 0.007349 | 0.167706 |
| WFDC21P | chr17q23.1 | WAP four-disulfide core domain 21,  pseudogene | 4.89337 | 5.42041 | -0.527045 | -1.44097 | 0.0064605 | 0.163699 |
| NEXN | chr1p31.1 | nexilin (F actin binding protein) | 4.64596 | 5.15513 | -0.50917 | -1.42323 | 0.0160661 | 0.195587 |
| NEURL3 | chr2q11.2 | neuralized E3 ubiquitin protein ligase 3 | 5.78031 | 6.19165 | -0.411339 | -1.32992 | 0.0148795 | 0.19252 |
| DUSP6 | chr12q22-q23 | dual specificity phosphatase 6 | 7.68683 | 8.31562 | -0.628789 | -1.54627 | 0.0013353 | 0.137103 |
| DUSP6 | chr12q22-q23 | dual specificity phosphatase 6 | 8.51956 | 9.06269 | -0.543127 | -1.45713 | 0.0050571 | 0.157506 |
| TLR4 | chr9q33.1 | toll-like receptor 4 | 5.54848 | 6.1872 | -0.638718 | -1.55694 | 0.0014378 | 0.140682 |
| DMXL2 | chr15q21.2 | Dmx-like 2 | 8.47084 | 8.99176 | -0.520919 | -1.43487 | 0.0048956 | 0.157154 |
| MPEG1 | chr11q12.1 | macrophage expressed 1 | 8.55782 | 9.07915 | -0.521339 | -1.43529 | 0.0164594 | 0.196619 |
| MPEG1 | chr11q12.1 | macrophage expressed 1 | 8.13288 | 8.63638 | -0.503497 | -1.41765 | 0.0138212 | 0.190686 |
| C5AR1 | chr19q13.3-  q13.4 | complement component 5a receptor 1 | 6.0598 | 6.60638 | -0.546582 | -1.46062 | 0.0128048 | 0.187439 |
| GAS2L3 | chr12q23.1 | growth arrest-specific 2 like 3 | 5.00364 | 5.45568 | -0.452039 | -1.36797 | 0.0182198 | 0.199412 |
| LILRB2 | chr19q13.4 | leukocyte immunoglobulin-like  receptor, subfamily B (with TM and ITIM domains), member 2 | 6.49053 | 6.93437 | -0.443841 | -1.36022 | 0.0155413 | 0.194293 |
| PDK4 | chr7q21.3 | pyruvate dehydrogenase kinase,  isozyme 4 | 6.75179 | 7.22351 | -0.471725 | -1.38677 | 0.0044949 | 0.154031 |
| NLN | chr5q12.3 | neurolysin (metallopeptidase M3  family) | 3.40557 | 3.85848 | -0.452912 | -1.3688 | 0.0076031 | 0.168472 |
| TLR4 | chr9q33.1 | toll-like receptor 4 | 5.20074 | 5.63093 | -0.430194 | -1.34741 | 0.0059358 | 0.160703 |
| SAMD9L | chr7q21.2 | sterile alpha motif domain containing 9  like | 7.38414 | 7.82952 | -0.445381 | -1.36167 | 0.006975 | 0.165923 |
| OAS2 /// OAS3 | chr12q24.2 | 2'-5'-oligoadenylate synthetase 2 /// 2'-  5'-oligoadenylate synthetase 3 | 8.68288 | 9.11278 | -0.429906 | -1.34715 | 0.0134573 | 0.189772 |
| FAR2 | chr12p11.22 | fatty acyl-CoA reductase 2 | 3.65404 | 4.14695 | -0.492914 | -1.40728 | 0.0082313 | 0.169323 |

| LOC101928  143 /// NUMB | chr14q24.3 | uncharacterized LOC101928143 /// numb homolog (Drosophila) | 6.00741 | 6.43075 | -0.423337 | -1.34103 | 0.0065141 | 0.163699 |
| --- | --- | --- | --- | --- | --- | --- | --- | --- |
| MSR1 | chr8p22 | macrophage scavenger receptor 1 | 3.9154 | 4.52642 | -0.611023 | -1.52734 | 0.0041271 | 0.151685 |
| ARMC9 | chr2q37.1 | armadillo repeat containing 9 | 4.22494 | 4.748 | -0.523058 | -1.437 | 0.006659 | 0.164139 |
| IGSF6 | chr16p12.2 | immunoglobulin superfamily, member  6 | 4.94383 | 5.44916 | -0.505331 | -1.41945 | 0.0060868 | 0.161323 |
| SPRED1 | chr15q14 | sprouty-related, EVH1 domain  containing 1 | 3.99146 | 4.46599 | -0.47453 | -1.38947 | 0.0069665 | 0.165836 |
| HTR2B | chr2q36.3-q37.1 | 5-hydroxytryptamine (serotonin)  receptor 2B, G protein-coupled | 3.25681 | 3.72797 | -0.471158 | -1.38622 | 0.0072642 | 0.167291 |
| NLN | chr5q12.3 | neurolysin (metallopeptidase M3  family) | 4.52759 | 4.93864 | -0.411051 | -1.32965 | 0.018061 | 0.198864 |
| SECTM1 | chr17q25 | secreted and transmembrane 1 | 6.39432 | 6.81729 | -0.422971 | -1.34069 | 0.0009257 | 0.128628 |
| ANKRD22 | chr10q23.31 | ankyrin repeat domain 22 | 5.09414 | 5.38339 | -0.289258 | -1.22201 | 0.0099475 | 0.175091 |
| ANKRD22 | chr10q23.31 | ankyrin repeat domain 22 | 4.99323 | 5.45399 | -0.460763 | -1.37627 | 0.0091454 | 0.172989 |
| HCAR3 | chr12q24.31 | hydroxycarboxylic acid receptor 3 | 4.41776 | 4.85556 | -0.437801 | -1.35454 | 0.0036364 | 0.150674 |
| FABP5 | chr8q21.13 | fatty acid binding protein 5 (psoriasis-  associated) | 6.6162 | 7.19073 | -0.574526 | -1.48919 | 0.0001696 | 0.111326 |
| IGSF6 | chr16p12.2 | immunoglobulin superfamily, member  6 | 7.57408 | 8.07442 | -0.500336 | -1.41454 | 0.0032689 | 0.149592 |
| MIR155HG | --- | MIR155 host gene | 7.51291 | 7.95868 | -0.44577 | -1.36204 | 0.0079899 | 0.168566 |
| MITF | chr3p14.2-p14.1 | microphthalmia-associated  transcription factor | 4.06993 | 4.54559 | -0.47566 | -1.39055 | 0.0012926 | 0.137103 |
| CLEC12A | chr12p13.2 | C-type lectin domain family 12,  member A | 6.45206 | 6.89463 | -0.442569 | -1.35902 | 0.0061963 | 0.161809 |
| C1GALT1C1 | chrXq24 | C1GALT1 specific chaperone 1 | 7.2174 | 7.62127 | -0.403879 | -1.32306 | 0.0094892 | 0.173123 |
| FGFR1OP2 | chr12p11.23 | FGFR1 oncogene partner 2 | 6.67032 | 7.04316 | -0.372845 | -1.2949 | 0.0046945 | 0.155506 |
| FXYD6 | chr11q23.3 | FXYD domain containing ion transport  regulator 6 | 5.6509 | 6.09075 | -0.43985 | -1.35646 | 0.0034692 | 0.150538 |
| LILRA1 | chr19q13.4 | leukocyte immunoglobulin-like  receptor, subfamily A (with TM domain), member 1 | 4.78706 | 5.18624 | -0.399189 | -1.31877 | 0.0038022 | 0.150735 |
| CCR1 | chr3p21 | chemokine (C-C motif) receptor 1 | 7.03858 | 7.52131 | -0.482728 | -1.39738 | 0.0096351 | 0.173245 |
| CCR1 | chr3p21 | chemokine (C-C motif) receptor 1 | 9.32979 | 9.79852 | -0.468735 | -1.3839 | 0.0116482 | 0.185171 |
| CCRL2 | chr3p21 | chemokine (C-C motif) receptor-like 2 | 6.90871 | 7.39576 | -0.487054 | -1.40158 | 0.0062154 | 0.161814 |
| JAKMIP2 | chr5q32 | janus kinase and microtubule  interacting protein 2 | 5.73062 | 6.15957 | -0.428953 | -1.34626 | 0.0171593 | 0.198289 |
| SLC44A1 | chr9q31.2 | solute carrier family 44 (choline  transporter), member 1 | 8.27884 | 8.71166 | -0.432814 | -1.34986 | 0.0110287 | 0.181825 |
| C3AR1 | chr12p13.31 | complement component 3a receptor 1 | 8.98906 | 9.52034 | -0.531281 | -1.44521 | 0.0020587 | 0.147757 |
| DRAM1 | chr12q23.2 | DNA-damage regulated autophagy  modulator 1 | 6.63937 | 7.07094 | -0.431568 | -1.3487 | 0.0039028 | 0.151155 |
| SASH1 | chr6q24.3 | SAM and SH3 domain containing 1 | 7.54827 | 8.03878 | -0.490508 | -1.40494 | 0.0041687 | 0.151901 |
| CLEC7A | chr12p13.2 | C-type lectin domain family 7, member  A | 8.56734 | 8.99147 | -0.424133 | -1.34177 | 0.0079557 | 0.168472 |
| GNB4 | chr3q26.33 | guanine nucleotide binding protein (G protein), beta polypeptide 4 | 7.84025 | 8.21856 | -0.378312 | -1.29982 | 0.0088457 | 0.172409 |
| PLSCR1 | chr3q23 | phospholipid scramblase 1 | 8.16234 | 8.51618 | -0.353844 | -1.27796 | 0.0161864 | 0.196004 |
| TLR7 | chrXp22.3 | toll-like receptor 7 | 6.65622 | 7.0517 | -0.39548 | -1.31538 | 0.0067036 | 0.164139 |
| LAP3 | chr4p15.32 | leucine aminopeptidase 3 | 9.90987 | 10.2884 | -0.378559 | -1.30004 | 0.0028773 | 0.148407 |
| JAK2 | chr9p24 | Janus kinase 2 | 6.1674 | 6.51888 | -0.351489 | -1.27588 | 0.0085615 | 0.170624 |

| CARD6 | chr5p13.1 | caspase recruitment domain family,  member 6 | 6.34421 | 6.71002 | -0.36581 | -1.28861 | 0.013885 | 0.190686 |
| --- | --- | --- | --- | --- | --- | --- | --- | --- |
| SLC31A2 | chr9q32 | solute carrier family 31 (copper  transporter), member 2 | 7.7274 | 8.07677 | -0.349372 | -1.27401 | 0.0093271 | 0.173053 |
| SASH1 | chr6q24.3 | SAM and SH3 domain containing 1 | 6.26177 | 6.67257 | -0.410805 | -1.32943 | 0.0042343 | 0.152075 |
| TCF7L2 | chr10q25.3 | transcription factor 7-like 2 (T-cell  specific, HMG-box) | 5.21564 | 5.56927 | -0.353624 | -1.27777 | 0.0072896 | 0.167406 |
| DUSP6 | chr12q22-q23 | dual specificity phosphatase 6 | 7.75259 | 8.14706 | -0.39447 | -1.31446 | 0.0156779 | 0.194492 |
| SLAMF7 | chr1q23.1-q24.1 | SLAM family member 7 | 9.18601 | 9.547 | -0.360991 | -1.28431 | 0.0085793 | 0.170624 |
| KMO | chr1q42-q44 | kynurenine 3-monooxygenase  (kynurenine 3-hydroxylase) | 7.16737 | 7.60504 | -0.437677 | -1.35442 | 0.0087399 | 0.17126 |
| KYNU | chr2q22.2 | kynureninase | 9.31442 | 9.69776 | -0.383345 | -1.30436 | 0.0129085 | 0.187472 |
| TFEC | chr7q31.2 | transcription factor EC | 8.68478 | 9.10154 | -0.416766 | -1.33493 | 0.0055448 | 0.158791 |
| KYNU | chr2q22.2 | kynureninase | 8.80203 | 9.12366 | -0.321626 | -1.24974 | 0.015798 | 0.194682 |
| IFI27 | chr14q32 | interferon, alpha-inducible protein 27 | 5.09922 | 5.55177 | -0.452554 | -1.36846 | 0.0030296 | 0.148407 |
| PPP1R12A | chr12q15-q21 | protein phosphatase 1, regulatory  subunit 12A | 8.47052 | 8.91859 | -0.448073 | -1.36422 | 0.0011249 | 0.134713 |
| HESX1 | chr3p14.3 | HESX homeobox 1 | 4.54957 | 5.14539 | -0.595823 | -1.51133 | 0.0009058 | 0.128628 |
| GBP1 | chr1p22.2 | guanylate binding protein 1, interferon-  inducible | 8.38509 | 8.9508 | -0.565707 | -1.48011 | 0.0008637 | 0.128628 |
| GBP1 | chr1p22.2 | guanylate binding protein 1, interferon-  inducible | 8.34717 | 8.84265 | -0.495476 | -1.40979 | 0.0013383 | 0.137103 |
| FAR2 | chr12p11.22 | fatty acyl-CoA reductase 2 | 4.7007 | 5.19436 | -0.493665 | -1.40802 | 0.0057227 | 0.158791 |
| MIR21 ///  VMP1 | chr17q23.1 | microRNA 21 /// vacuole membrane  protein 1 | 8.11428 | 8.57612 | -0.461842 | -1.3773 | 0.0049828 | 0.157506 |
| IFIT2 | chr10q23.31 | interferon-induced protein with  tetratricopeptide repeats 2 | 8.39287 | 8.82519 | -0.432317 | -1.3494 | 0.0078637 | 0.168472 |
| IDO2 | chr8p11.21 | indoleamine 2,3-dioxygenase 2 | 4.22892 | 4.71914 | -0.490221 | -1.40466 | 0.0018287 | 0.145897 |
| TNFSF13B | chr13q32-q34 | tumor necrosis factor (ligand)  superfamily, member 13b | 8.91136 | 9.45241 | -0.541047 | -1.45503 | 0.0003614 | 0.111326 |
| TNFSF13B | chr13q32-q34 | tumor necrosis factor (ligand)  superfamily, member 13b | 9.06572 | 9.51952 | -0.453796 | -1.36964 | 0.0022649 | 0.148407 |
| ETV7 | chr6p21 | ets variant 7 | 4.12268 | 4.52448 | -0.401799 | -1.32115 | 0.001693 | 0.145076 |
| JAK2 | chr9p24 | Janus kinase 2 | 7.46004 | 7.83177 | -0.371732 | -1.29391 | 0.0050733 | 0.157506 |
| PPP1R12A | chr12q15-q21 | protein phosphatase 1, regulatory  subunit 12A | 8.69479 | 9.06301 | -0.368217 | -1.29076 | 0.0095011 | 0.173123 |
| PPP1R12A | chr12q15-q21 | protein phosphatase 1, regulatory  subunit 12A | 9.1452 | 9.49938 | -0.354177 | -1.27826 | 0.0065425 | 0.163699 |
| ATP8B4 | chr15q21.2 | ATPase, class I, type 8B, member 4 | 2.83916 | 3.21779 | -0.378627 | -1.3001 | 0.0079651 | 0.16854 |
| OAS1 | chr12q24.2 | 2'-5'-oligoadenylate synthetase 1 | 8.24714 | 8.64438 | -0.39724 | -1.31699 | 0.0102269 | 0.177837 |
| IFIT3 | chr10q24 | interferon-induced protein with  tetratricopeptide repeats 3 | 10.0474 | 10.5149 | -0.467472 | -1.38268 | 0.0091034 | 0.172989 |
| HERC5 | chr4q22.1 | HECT and RLD domain containing E3  ubiquitin protein ligase 5 | 9.3975 | 9.87348 | -0.47598 | -1.39086 | 0.0057947 | 0.160103 |
| ISG15 | chr1p36.33 | ISG15 ubiquitin-like modifier | 10.4251 | 10.8329 | -0.407723 | -1.32659 | 0.0119518 | 0.186018 |
| IFIT3 | chr10q24 | interferon-induced protein with  tetratricopeptide repeats 3 | 8.94901 | 9.31817 | -0.369158 | -1.2916 | 0.01736 | 0.198289 |
| PNPT1 | chr2p15 | polyribonucleotide  nucleotidyltransferase 1 | 8.40814 | 8.83382 | -0.425684 | -1.34321 | 0.0081844 | 0.169323 |
| USP18 | chr22q11.21 | ubiquitin specific peptidase 18 | 6.71678 | 7.13458 | -0.417794 | -1.33588 | 0.0087887 | 0.171813 |
| OAS1 | chr12q24.2 | 2'-5'-oligoadenylate synthetase 1 | 8.41509 | 8.85611 | -0.441028 | -1.35757 | 0.0018545 | 0.14618 |
| SAMD9L | chr7q21.2 | sterile alpha motif domain containing 9  like | 8.75006 | 9.18744 | -0.437384 | -1.35415 | 0.0033033 | 0.149592 |
| GMPR | chr6p23 | guanosine monophosphate reductase | 5.92537 | 6.31586 | -0.390495 | -1.31084 | 0.0067756 | 0.164604 |

| IFI44 | chr1p31.1 | interferon-induced protein 44 | 9.43649 | 9.7604 | -0.323909 | -1.25172 | 0.0047141 | 0.155506 |
| --- | --- | --- | --- | --- | --- | --- | --- | --- |
| IFIH1 | chr2q24 | interferon induced, with helicase C  domain 1 | 8.87689 | 9.28853 | -0.411643 | -1.3302 | 0.0009263 | 0.128628 |
| DDX58 | chr9p12 | DEAD (Asp-Glu-Ala-Asp) box  polypeptide 58 | 7.42187 | 7.74013 | -0.318261 | -1.24683 | 0.0057183 | 0.158791 |
| PARP9 | chr3q21 | poly(ADP-ribose) polymerase family  member 9 | 8.67831 | 9.01872 | -0.340412 | -1.26612 | 0.0119967 | 0.186078 |
| PARP14 | chr3q21.1 | poly(ADP-ribose) polymerase family  member 14 | 9.07846 | 9.37335 | -0.294885 | -1.22679 | 0.0165115 | 0.196619 |
| DTX3L | chr3q21.1 | deltex 3 like, E3 ubiquitin ligase | 8.95256 | 9.22587 | -0.273313 | -1.20858 | 0.016862 | 0.197956 |
| SAMD9L | chr7q21.2 | sterile alpha motif domain containing 9  like | 8.3698 | 8.81228 | -0.442486 | -1.35894 | 0.0126897 | 0.187417 |
| OSBPL1A | chr18q11.1 | oxysterol binding protein-like 1A | 4.14029 | 4.55037 | -0.410075 | -1.32876 | 0.002679 | 0.148407 |
| FAM46A | chr6q14 | family with sequence similarity 46,  member A | 6.0324 | 6.45551 | -0.423111 | -1.34082 | 0.0073957 | 0.167706 |
| TCF7L2 | chr10q25.3 | transcription factor 7-like 2 (T-cell  specific, HMG-box) | 5.63811 | 6.03648 | -0.398374 | -1.31802 | 0.0034464 | 0.150538 |
| TCF7L2 | chr10q25.3 | transcription factor 7-like 2 (T-cell  specific, HMG-box) | 7.41012 | 7.74422 | -0.334102 | -1.26059 | 0.0119394 | 0.186018 |
| SAMD9 | chr7q21.2 | sterile alpha motif domain containing 9 | 6.81928 | 7.37167 | -0.552387 | -1.46651 | 0.0002341 | 0.111326 |
| DDX58 | chr9p12 | DEAD (Asp-Glu-Ala-Asp) box  polypeptide 58 | 7.87026 | 8.31804 | -0.447779 | -1.36394 | 0.0021135 | 0.147757 |
| SAMD9 | chr7q21.2 | sterile alpha motif domain containing 9 | 8.53587 | 8.96142 | -0.425547 | -1.34308 | 0.0009304 | 0.128628 |
| STK3 | chr8q22.2 | serine/threonine kinase 3 | 6.10076 | 6.46271 | -0.361951 | -1.28516 | 0.0122195 | 0.186495 |
| CD80 | chr3q13.3-q21 | CD80 molecule | 5.68301 | 6.04864 | -0.365629 | -1.28844 | 0.0013058 | 0.137103 |
| PIK3AP1 | chr10q24.1 | phosphoinositide-3-kinase adaptor  protein 1 | 9.12277 | 9.47709 | -0.354318 | -1.27838 | 0.0075451 | 0.168472 |
| TDRD7 | chr9q22.33 | tudor domain containing 7 | 7.82423 | 8.15932 | -0.335094 | -1.26146 | 0.0033077 | 0.149592 |
| METTL7B | chr12q13.2 | methyltransferase like 7B | 3.57882 | 3.89239 | -0.313573 | -1.24278 | 0.0158251 | 0.194832 |
| IFIT5 | chr10q23.31 | interferon-induced protein with  tetratricopeptide repeats 5 | 7.55292 | 7.9206 | -0.367678 | -1.29027 | 0.004763 | 0.155737 |
| OAS2 | chr12q24.2 | 2'-5'-oligoadenylate synthetase 2 | 6.53711 | 6.86377 | -0.326664 | -1.25411 | 0.0076812 | 0.168472 |
| --- | --- | --- | 4.26863 | 4.5639 | -0.295267 | -1.22711 | 0.0100965 | 0.176392 |
| NAMPT | chr7q22.3 | nicotinamide  phosphoribosyltransferase | 8.33848 | 8.60889 | -0.270409 | -1.20615 | 0.0098793 | 0.174971 |
| PI4K2B | chr4p15.2 | phosphatidylinositol 4-kinase type 2  beta | 7.1835 | 7.50118 | -0.317674 | -1.24632 | 0.0032043 | 0.149592 |
| RABGAP1L | chr1q24 | RAB GTPase activating protein 1-like | 8.31324 | 8.63017 | -0.316929 | -1.24568 | 0.0012572 | 0.136585 |
| NT5C3A | chr7p14.3 | 5'-nucleotidase, cytosolic IIIA | 9.80586 | 10.0852 | -0.279381 | -1.21367 | 0.0023056 | 0.148407 |
| IFIT5 | chr10q23.31 | interferon-induced protein with  tetratricopeptide repeats 5 | 8.60524 | 8.8742 | -0.268957 | -1.20494 | 0.0139516 | 0.190686 |
| CFB | chr6p21.3 | complement factor B | 3.34637 | 3.81913 | -0.472758 | -1.38776 | 0.0066504 | 0.164139 |
| MSR1 | chr8p22 | macrophage scavenger receptor 1 | 3.5762 | 3.93183 | -0.355625 | -1.27954 | 0.0125168 | 0.187104 |
| PTX3 | chr3q25 | pentraxin 3, long | 3.00507 | 3.35988 | -0.354814 | -1.27882 | 0.0065516 | 0.163699 |
| CCL3 /// CCL3L1 /// CCL3L3 | chr17q12 /// chr17q21.1 | chemokine (C-C motif) ligand 3 ///  chemokine (C-C motif) ligand 3-like 1  /// chemokine (C-C motif) ligand 3-like 3 | 6.64787 | 7.0517 | -0.403828 | -1.32301 | 0.0052319 | 0.157641 |
| MGLL | chr3q21.3 | monoglyceride lipase | 6.7591 | 7.09795 | -0.338846 | -1.26474 | 0.0034974 | 0.150538 |
| MCTP1 | chr5q15 | multiple C2 domains, transmembrane  1 | 5.03747 | 5.34305 | -0.305579 | -1.23591 | 0.0008653 | 0.128628 |
| AK4 | chr1p31.3 | adenylate kinase 4 | 5.64184 | 6.00953 | -0.367686 | -1.29028 | 0.015632 | 0.194408 |
| HNMT | chr2q22.1 | histamine N-methyltransferase | 7.7375 | 8.13636 | -0.398861 | -1.31847 | 0.0072302 | 0.167222 |

| CPVL | chr7p15.1 | carboxypeptidase, vitellogenic-like | 5.2429 | 5.66814 | -0.425238 | -1.34279 | 0.0055348 | 0.158791 |
| --- | --- | --- | --- | --- | --- | --- | --- | --- |
| CLEC4A | chr12p13 | C-type lectin domain family 4, member  A | 6.80355 | 7.33775 | -0.534203 | -1.44814 | 0.0010724 | 0.13333 |
| CLEC4A | chr12p13 | C-type lectin domain family 4, member  A | 6.61093 | 7.07844 | -0.467515 | -1.38273 | 0.0024598 | 0.148407 |
| PDGFC | chr4q32 | platelet derived growth factor C | 6.22342 | 6.64064 | -0.417216 | -1.33535 | 0.0053716 | 0.158323 |
| CARD16 | chr11q22 | caspase recruitment domain family,  member 16 | 8.36686 | 8.78004 | -0.413179 | -1.33162 | 0.0119597 | 0.186018 |
| LY96 | chr8q21.11 | lymphocyte antigen 96 | 7.6685 | 8.20699 | -0.538487 | -1.45245 | 0.0007948 | 0.128628 |
| CFD | chr19p13.3 | complement factor D (adipsin) | 6.44904 | 6.81645 | -0.367408 | -1.29003 | 0.0126787 | 0.187417 |
| TFPI | chr2q32 | tissue factor pathway inhibitor  (lipoprotein-associated coagulation inhibitor) | 4.23524 | 4.56828 | -0.333043 | -1.25967 | 0.0171735 | 0.198289 |
| HNMT | chr2q22.1 | histamine N-methyltransferase | 4.16575 | 4.51181 | -0.346058 | -1.27108 | 0.0120204 | 0.186078 |
| TXN | chr9q31 | thioredoxin | 9.0489 | 9.37965 | -0.330752 | -1.25767 | 0.0089076 | 0.172413 |
| LRRK2 | chr12q12 | leucine-rich repeat kinase 2 | 5.31933 | 5.63819 | -0.318863 | -1.24735 | 0.0057892 | 0.160063 |
| CD86 | chr3q21 | CD86 molecule | 7.05532 | 7.39582 | -0.340504 | -1.2662 | 0.0071353 | 0.166808 |
| LACC1 | chr13q14.11 | laccase (multicopper oxidoreductase) domain containing 1 | 8.35695 | 8.66147 | -0.304521 | -1.23501 | 0.0162152 | 0.196072 |
| TLR8 | chrXp22 | toll-like receptor 8 | 8.03749 | 8.3716 | -0.334104 | -1.26059 | 0.0082469 | 0.169323 |
| LMO2 | chr11p13 | LIM domain only 2 (rhombotin-like 1) | 7.36203 | 7.66147 | -0.29944 | -1.23067 | 0.0155356 | 0.194293 |
| ADRBK2 | chr22q12.1 | adrenergic, beta, receptor kinase 2 | 5.9974 | 6.29167 | -0.294263 | -1.22626 | 0.0070367 | 0.166492 |
| CASP1 | chr11q23 | caspase 1 | 8.40191 | 8.71163 | -0.309711 | -1.23946 | 0.0050169 | 0.157506 |
| CASP1 | chr11q23 | caspase 1 | 9.59402 | 9.92572 | -0.331702 | -1.2585 | 0.0026575 | 0.148407 |
| CASP1 | chr11q23 | caspase 1 | 9.95256 | 10.2727 | -0.320127 | -1.24844 | 0.0015494 | 0.14471 |
| CASP1 | chr11q23 | caspase 1 | 9.58479 | 9.86515 | -0.280362 | -1.2145 | 0.0078326 | 0.168472 |
| CREG1 | chr1q24 | cellular repressor of E1A-stimulated  genes 1 | 8.39207 | 8.66292 | -0.270846 | -1.20652 | 0.0095049 | 0.173123 |
| IRAK3 | chr12q14.3 | interleukin 1 receptor associated  kinase 3 | 5.96442 | 6.38004 | -0.415619 | -1.33387 | 0.0155227 | 0.194293 |
| MARCKS | chr6q22.2 | myristoylated alanine-rich protein  kinase C substrate | 8.67247 | 9.08039 | -0.40792 | -1.32677 | 0.00595 | 0.160853 |
| TFEC | chr7q31.2 | transcription factor EC | 7.42045 | 7.87857 | -0.458114 | -1.37374 | 0.0013984 | 0.138618 |
| LAMP2 | chrXq24 | lysosomal-associated membrane  protein 2 | 6.70818 | 7.03168 | -0.323502 | -1.25136 | 0.0151492 | 0.193396 |
| --- | --- | --- | 3.32423 | 3.64704 | -0.322806 | -1.25076 | 0.0070798 | 0.166748 |
| CPNE8 | chr12q12 | copine VIII | 4.66559 | 5.09587 | -0.43028 | -1.34749 | 0.0017078 | 0.145076 |
| MCTP1 | chr5q15 | multiple C2 domains, transmembrane  1 | 3.61406 | 3.9982 | -0.38414 | -1.30508 | 0.0164536 | 0.196619 |
| ACSL4 | chrXq22.3-q23 | acyl-CoA synthetase long-chain family  member 4 | 5.7073 | 6.07506 | -0.367757 | -1.29035 | 0.0059717 | 0.160924 |
| NFE2L3 | chr7p15.2 | nuclear factor, erythroid 2-like 3 | 3.94784 | 4.34973 | -0.401888 | -1.32124 | 0.0016206 | 0.145076 |
| GPR180 | chr13q32.1 | G protein-coupled receptor 180 | 5.40382 | 5.74458 | -0.340757 | -1.26642 | 0.0068896 | 0.165562 |
| RAD51AP1 | chr12p13.2-  p13.1 | RAD51 associated protein 1 | 4.70555 | 5.03073 | -0.325182 | -1.25282 | 0.0060481 | 0.161186 |
| DNAJB4 | chr1p31.1 | DnaJ (Hsp40) homolog, subfamily B,  member 4 | 5.07755 | 5.40717 | -0.329616 | -1.25668 | 0.001525 | 0.143188 |
| ZNRF2 | chr7p14.3 | zinc and ring finger 2, E3 ubiquitin  protein ligase | 6.98517 | 7.29669 | -0.311527 | -1.24102 | 0.0054738 | 0.158791 |
| USP6NL | chr10p13 | USP6 N-terminal like | 3.7516 | 4.1178 | -0.366202 | -1.28895 | 0.0007085 | 0.128628 |
| CENPK | chr5q12.3 | centromere protein K | 2.95929 | 3.28859 | -0.329296 | -1.2564 | 0.000821 | 0.128628 |
| ATP6V1C1 | chr8q22.3 | ATPase, H+ transporting, lysosomal  42kDa, V1 subunit C1 | 7.01643 | 7.40554 | -0.38911 | -1.30959 | 0.0005906 | 0.128084 |

| --- | --- | --- | 5.76862 | 6.11557 | -0.346946 | -1.27187 | 0.0082874 | 0.169329 |
| --- | --- | --- | --- | --- | --- | --- | --- | --- |
| ME1 | chr6q12 | malic enzyme 1, NADP(+)-dependent,  cytosolic | 3.77317 | 4.1127 | -0.339537 | -1.26535 | 0.0030493 | 0.148407 |
| LOC105379  287 /// MTHFD2L | chr4q13.3 | uncharacterized LOC105379287 ///  methylenetetrahydrofolate dehydrogenase (NADP+ dependent) 2- like | 5.26303 | 5.52765 | -0.264619 | -1.20132 | 0.0170796 | 0.198257 |
| --- | --- | --- | 3.51268 | 4.00283 | -0.490149 | -1.40459 | 0.0154912 | 0.194281 |
| NRG1 | chr8p12 | neuregulin 1 | 3.34458 | 3.78117 | -0.436589 | -1.3534 | 0.0162753 | 0.196072 |
| SOD2 | chr6q25.3 | superoxide dismutase 2, mitochondrial | 7.73685 | 8.30929 | -0.572436 | -1.48703 | 0.0059103 | 0.160245 |
| --- | --- | --- | 4.12969 | 4.67848 | -0.548792 | -1.46286 | 0.0037175 | 0.150735 |
| FCER1A | chr1q23 | Fc fragment of IgE, high affinity I,  receptor for; alpha polypeptide | 4.51081 | 5.05031 | -0.539499 | -1.45347 | 0.0027725 | 0.148407 |
| MARCH1 | chr4q32.2 | membrane associated ring finger 1 | 6.24821 | 6.87604 | -0.627834 | -1.54524 | 0.0002481 | 0.111326 |
| SLC44A1 | chr9q31.2 | solute carrier family 44 (choline  transporter), member 1 | 5.91112 | 6.39414 | -0.483019 | -1.39767 | 0.0113857 | 0.183374 |
| MITF | chr3p14.2-p14.1 | microphthalmia-associated  transcription factor | 4.25949 | 4.77374 | -0.514245 | -1.42825 | 0.0001621 | 0.111326 |
| PAPSS2 | chr10q24 | 3'-phosphoadenosine 5'-  phosphosulfate synthase 2 | 4.5795 | 4.99171 | -0.412217 | -1.33073 | 0.0102502 | 0.177837 |
| --- | --- | --- | 4.22563 | 4.74078 | -0.515149 | -1.42914 | 0.0178249 | 0.198662 |
| --- | --- | --- | 3.52723 | 3.98344 | -0.456216 | -1.37194 | 0.0125324 | 0.187104 |
| LINC01296 | chr14q11.2 | long intergenic non-protein coding  RNA 1296 | 3.40622 | 3.85029 | -0.444068 | -1.36043 | 0.014638 | 0.191814 |
| ZNF479 | chr7p11.2 | zinc finger protein 479 | 5.04741 | 5.45381 | -0.406399 | -1.32537 | 0.0073123 | 0.167509 |
| FGF7 | chr15q21.2 | fibroblast growth factor 7 | 3.45637 | 3.81172 | -0.355351 | -1.2793 | 0.0116122 | 0.185171 |
| SCGB1C1  /// SCGB1C2 | chr11p15.5 /// chr17p | secretoglobin, family 1C, member 1 /// secretoglobin, family 1C, member 2 | 3.89134 | 4.22481 | -0.333473 | -1.26004 | 0.0100376 | 0.175647 |
| LOC105377  458 | --- | uncharacterized LOC105377458 | 2.32896 | 2.71027 | -0.381317 | -1.30253 | 0.0155143 | 0.194293 |
| --- | --- | --- | 2.68571 | 3.05894 | -0.373227 | -1.29525 | 0.0050965 | 0.157506 |
| --- | --- | --- | 3.30961 | 3.62966 | -0.320052 | -1.24838 | 0.0040662 | 0.151685 |
| --- | --- | --- | 3.31448 | 3.68796 | -0.373482 | -1.29548 | 0.0180709 | 0.198866 |
| MFAP3L | chr4q32.3 | microfibrillar associated protein 3 like | 5.51958 | 5.89242 | -0.372839 | -1.2949 | 0.015103 | 0.193396 |
| FLJ22763 | chr3q13.13 | uncharacterized LOC401081 | 3.49723 | 3.89645 | -0.399223 | -1.3188 | 0.0140914 | 0.190686 |
| CAPS2 | chr12q14.1 | calcyphosine 2 | 2.43402 | 2.86123 | -0.427214 | -1.34463 | 0.0036825 | 0.150735 |
| ACKR4 | chr3q22 | atypical chemokine receptor 4 | 2.56248 | 2.90829 | -0.345814 | -1.27087 | 0.0112882 | 0.182785 |
| LTBP1 | chr2p22-p21 | latent transforming growth factor beta  binding protein 1 | 4.5493 | 4.86603 | -0.316732 | -1.24551 | 0.0066602 | 0.164139 |
| NID1 | chr1q43 | nidogen 1 | 4.01033 | 4.52426 | -0.51393 | -1.42793 | 0.0102361 | 0.177837 |
| HDAC9 | chr7p21.1 | histone deacetylase 9 | 5.3555 | 5.81994 | -0.464437 | -1.37978 | 0.0110545 | 0.181975 |
| --- | --- | --- | 5.10581 | 5.56882 | -0.463001 | -1.37841 | 0.0153678 | 0.193848 |
| PRR16 | chr5q23.1 | proline rich 16 | 4.29126 | 4.68458 | -0.393319 | -1.31341 | 0.0112482 | 0.182674 |
| MS4A14 | chr11q12.2 | membrane-spanning 4-domains,  subfamily A, member 14 | 5.66361 | 6.11114 | -0.44753 | -1.3637 | 0.0043266 | 0.1529 |
| --- | --- | --- | 4.18795 | 4.58822 | -0.400273 | -1.31976 | 0.0075451 | 0.168472 |
| SERPINA1 | chr14q32.1 | serpin peptidase inhibitor, clade A  (alpha-1 antiproteinase, antitrypsin), member 1 | 8.83958 | 9.21398 | -0.374404 | -1.2963 | 0.0178255 | 0.198662 |
| SERPINA1 | chr14q32.1 | serpin peptidase inhibitor, clade A  (alpha-1 antiproteinase, antitrypsin), member 1 | 8.32326 | 8.69363 | -0.37037 | -1.29268 | 0.014609 | 0.191814 |
| FRY | chr13q13.1 | FRY microtubule binding protein | 4.97899 | 5.34855 | -0.369556 | -1.29196 | 0.0086431 | 0.170762 |

| RTN1 | chr14q23.1 | reticulon 1 | 5.92753 | 6.27287 | -0.345334 | -1.27045 | 0.0066075 | 0.16393 |
| --- | --- | --- | --- | --- | --- | --- | --- | --- |
| CXCL9 | chr4q21 | chemokine (C-X-C motif) ligand 9 | 5.24029 | 5.66066 | -0.420369 | -1.33827 | 0.0068913 | 0.165562 |
| ARG2 | chr14q24.1 | arginase 2 | 4.13605 | 4.60493 | -0.468882 | -1.38404 | 0.0023676 | 0.148407 |
| EPS8 | chr12p12.3 | epidermal growth factor receptor  pathway substrate 8 | 3.89858 | 4.32968 | -0.431101 | -1.34826 | 0.0013102 | 0.137103 |
| CPED1 | chr7q31.31 | cadherin-like and PC-esterase domain  containing 1 | 3.54651 | 3.94018 | -0.393668 | -1.31373 | 0.0033293 | 0.149592 |
| FXYD2 | chr11q23 | FXYD domain containing ion transport  regulator 2 | 5.11707 | 5.4822 | -0.365133 | -1.288 | 0.0115229 | 0.184291 |
| ANGPT1 | chr8q23.1 | angiopoietin 1 | 2.51726 | 2.8674 | -0.350143 | -1.27469 | 0.0162989 | 0.196072 |
| CCL24 | chr7q11.23 | chemokine (C-C motif) ligand 24 | 3.73945 | 4.08053 | -0.341082 | -1.26671 | 0.0031163 | 0.148407 |
| SORT1 | chr1p13.3\|1p21.  3-p13.1 | sortilin 1 | 6.5627 | 6.98345 | -0.42075 | -1.33862 | 0.0019378 | 0.146411 |
| FKBP1B | chr2p23.3 | FK506 binding protein 1B | 4.86377 | 5.32505 | -0.461277 | -1.37676 | 7.566E-05 | 0.111326 |
| IFNB1 | chr9p21 | interferon, beta 1, fibroblast | 2.34392 | 2.66719 | -0.323272 | -1.25116 | 0.0020955 | 0.147757 |
| MYO1B | chr2q12-q34 | myosin IB | 3.4593 | 3.87296 | -0.413661 | -1.33206 | 0.0014883 | 0.141716 |
| ZBTB1 | chr14q23.3 | zinc finger and BTB domain containing  1 | 5.24267 | 5.61692 | -0.37425 | -1.29617 | 0.0083505 | 0.16997 |
| ZSCAN5A | chr19q13.43 | zinc finger and SCAN domain  containing 5A | 3.28116 | 3.59449 | -0.313324 | -1.24257 | 0.0098321 | 0.174634 |
| TNFRSF10D | chr8p21 | tumor necrosis factor receptor  superfamily, member 10d, decoy with truncated death domain | 5.90867 | 6.21589 | -0.307226 | -1.23733 | 0.0110238 | 0.181825 |
| MTO1 | chr6q13 | mitochondrial tRNA translation  optimization 1 | 3.80299 | 4.13811 | -0.335122 | -1.26148 | 0.0065202 | 0.163699 |
| GBP4 | chr1p22.2 | guanylate binding protein 4 | 6.72033 | 7.01095 | -0.290618 | -1.22316 | 0.015381 | 0.193873 |
| PLS1 | chr3q23 | plastin 1 | 4.46937 | 4.80939 | -0.340021 | -1.26577 | 0.0055143 | 0.158791 |
| TMPRSS3 | chr21q22.3 | transmembrane protease, serine 3 | 3.05503 | 3.3354 | -0.280368 | -1.2145 | 0.0126409 | 0.187372 |
| KIF16B | chr20p11.23 | kinesin family member 16B | 4.90229 | 5.20792 | -0.30563 | -1.23596 | 0.014663 | 0.191883 |
| LGALS2 | chr22q13.1 | lectin, galactoside-binding, soluble, 2 | 6.20241 | 6.68772 | -0.48531 | -1.39989 | 0.0044713 | 0.153894 |
| AATBC | chr21q22.3 | apoptosis associated transcript in  bladder cancer | 5.51104 | 5.97083 | -0.459781 | -1.37533 | 0.0002052 | 0.111326 |
| DCANP1 /// TIFAB | chr5q31.1 | dendritic cell-associated nuclear  protein /// TRAF-interacting protein with forkhead-associated domain, family member B | 5.19546 | 5.67334 | -0.477877 | -1.39269 | 0.0002394 | 0.111326 |
| RASSF4 | chr10q11.21 | Ras association (RalGDS/AF-6) domain  family member 4 | 5.79034 | 6.14343 | -0.353089 | -1.27729 | 0.0068244 | 0.164807 |
| --- | --- | --- | 4.34864 | 4.68734 | -0.338703 | -1.26462 | 0.0024774 | 0.148407 |
| SUMO1 | chr2q33 | small ubiquitin-like modifier 1 | 3.45611 | 3.86885 | -0.41274 | -1.33121 | 0.0083315 | 0.169938 |
| IKBIP | chr12q23.1 | IKBKB interacting protein | 4.7551 | 5.14353 | -0.388427 | -1.30897 | 0.0035603 | 0.150674 |
| --- | --- | --- | 3.14822 | 3.48314 | -0.334914 | -1.2613 | 0.0135989 | 0.190151 |
| C1QC | chr1p36.11 | complement component 1, q  subcomponent, C chain | 2.98175 | 3.29018 | -0.308429 | -1.23836 | 0.0104529 | 0.178983 |
| A2M | chr12p13.31 | alpha-2-macroglobulin | 3.82156 | 4.11119 | -0.289636 | -1.22233 | 0.0053894 | 0.158555 |
| ARSD | chrXp22.3 | arylsulfatase D | 3.56734 | 3.88006 | -0.312727 | -1.24205 | 0.0024323 | 0.148407 |
| --- | --- | --- | 4.84887 | 5.14107 | -0.292205 | -1.22451 | 0.0102818 | 0.177873 |
| GDF15 | chr19p13.11 | growth differentiation factor 15 | 3.81446 | 4.17399 | -0.359525 | -1.283 | 0.0047377 | 0.155558 |
| SOX15 | chr17p13.1 | SRY box 15 | 3.8347 | 4.11143 | -0.276727 | -1.21144 | 0.0129058 | 0.187472 |
| CLEC1A | chr12p13.2 | C-type lectin domain family 1, member  A | 3.34702 | 3.70675 | -0.359727 | -1.28318 | 0.0012616 | 0.136585 |
| SPACA3 | chr17q11.2 | sperm acrosome associated 3 | 2.66214 | 2.94083 | -0.278684 | -1.21309 | 0.0003081 | 0.111326 |
| MTMR11 | chr1q21.2 | myotubularin related protein 11 | 5.91453 | 6.2193 | -0.30477 | -1.23522 | 0.0023896 | 0.148407 |
| --- | --- | --- | 7.59929 | 7.97941 | -0.38012 | -1.30145 | 0.0007972 | 0.128628 |
| FZD2 | chr17q21.31 | frizzled class receptor 2 | 5.57929 | 5.92093 | -0.341638 | -1.26719 | 0.0031202 | 0.148407 |

| S100A4 | chr1q21 | S100 calcium binding protein A4 | 11.1549 | 11.4313 | -0.27639 | -1.21116 | 0.0054391 | 0.158789 |
| --- | --- | --- | --- | --- | --- | --- | --- | --- |
| GLA | chrXq22 | galactosidase, alpha | 7.75553 | 8.02862 | -0.273087 | -1.20839 | 0.0048964 | 0.157154 |
| CD180 | chr5q12 | CD180 molecule | 7.51868 | 7.78723 | -0.26855 | -1.2046 | 0.0028062 | 0.148407 |
| ACVR2A | chr2q22.3 | activin A receptor type IIA | 5.68686 | 5.98319 | -0.296329 | -1.22802 | 0.0046918 | 0.155506 |
| TMEM176A | chr7q36.1 | transmembrane protein 176A | 6.32908 | 6.59633 | -0.26725 | -1.20351 | 0.0003031 | 0.111326 |
| SIPA1L1 | chr14q24.2 | signal-induced proliferation-associated  1 like 1 | 5.02438 | 5.35751 | -0.333131 | -1.25974 | 0.0001487 | 0.111326 |
| AZI2 | chr3p24.1 | 5-azacytidine induced 2 | 6.09648 | 6.42683 | -0.33035 | -1.25732 | 0.0037939 | 0.150735 |
| KANSL1L | chr2q34 | KAT8 regulatory NSL complex subunit 1  like | 3.35603 | 3.6497 | -0.293668 | -1.22575 | 0.0044741 | 0.153894 |
| CBWD2 | chr2q13 | COBW domain containing 2 | 5.51548 | 5.80939 | -0.293909 | -1.22596 | 0.0034907 | 0.150538 |
| SEH1L | chr18p11.21 | SEH1-like nucleoporin | 5.37302 | 5.66931 | -0.296283 | -1.22798 | 0.0013559 | 0.137133 |
| SPTSSA | chr14q13.1 | serine palmitoyltransferase, small  subunit A | 6.68548 | 7.00295 | -0.317462 | -1.24614 | 0.0001521 | 0.111326 |
| LYZ | chr12q15 | lysozyme | 11.9681 | 12.2438 | -0.275699 | -1.21058 | 0.0006858 | 0.128628 |
| GCLC | chr6p12 | glutamate-cysteine ligase, catalytic  subunit | 7.78571 | 8.06488 | -0.279172 | -1.2135 | 0.0031078 | 0.148407 |
| TRAFD1 | chr12q | TRAF-type zinc finger domain  containing 1 | 7.52146 | 7.79985 | -0.278389 | -1.21284 | 0.0012884 | 0.137103 |
| UTRN | chr6q24 | utrophin | 8.5882 | 8.86236 | -0.274162 | -1.20929 | 0.0011018 | 0.133875 |
| TMEM158 | chr3p21.3 | transmembrane protein 158  (gene/pseudogene) | 4.43544 | 4.71252 | -0.277077 | -1.21174 | 0.0145056 | 0.191711 |
| IFITM3 | chr11p15.5 | interferon induced transmembrane  protein 3 | 10.3523 | 10.618 | -0.265716 | -1.20223 | 0.0129175 | 0.187472 |
| ARL6IP6 | chr2q23.3 | ADP-ribosylation factor like GTPase 6  interacting protein 6 | 5.47614 | 5.78241 | -0.306273 | -1.23651 | 0.00244 | 0.148407 |
| AMN1 | chr12p11.21 | antagonist of mitotic exit network 1  homolog | 5.56218 | 5.86039 | -0.298214 | -1.22962 | 0.0028659 | 0.148407 |
| RAB20 | chr13q34 | RAB20, member RAS oncogene family | 6.94235 | 7.33884 | -0.396493 | -1.3163 | 0.0008401 | 0.128628 |
| TCF7L2 | chr10q25.3 | transcription factor 7-like 2 (T-cell  specific, HMG-box) | 5.72522 | 6.11159 | -0.386366 | -1.3071 | 0.0012405 | 0.136585 |
| HTR7 | chr10q21-q24 | 5-hydroxytryptamine (serotonin) receptor 7, adenylate cyclase-coupled | 3.18565 | 3.50813 | -0.322479 | -1.25048 | 0.0051074 | 0.157506 |
| SSFA2 | chr2q31.3 | sperm specific antigen 2 | 7.35071 | 7.63749 | -0.286782 | -1.21992 | 0.0138112 | 0.190686 |
| MKLN1 | chr7q32 | muskelin 1, intracellular mediator  containing kelch motifs | 6.86272 | 7.15704 | -0.294319 | -1.22631 | 0.0112716 | 0.182729 |
| TMEM206 | chr1q32.3 | transmembrane protein 206 | 7.29455 | 7.56215 | -0.267607 | -1.20381 | 0.0022834 | 0.148407 |
| KIAA0040 | chr1q25.1 | KIAA0040 | 6.44569 | 6.71114 | -0.265445 | -1.20201 | 0.0104176 | 0.178933 |
| FNIP2 | chr4q32.1 | folliculin interacting protein 2 | 7.82037 | 8.12629 | -0.305919 | -1.23621 | 0.0057065 | 0.158791 |
| AKIRIN2 | chr6q15 | akirin 2 | 7.83856 | 8.14123 | -0.30267 | -1.23342 | 0.00112 | 0.134537 |
| GM2A | chr5q33.1 | GM2 ganglioside activator | 5.62188 | 5.89762 | -0.275741 | -1.21062 | 0.0047373 | 0.155558 |
| OSBPL1A | chr18q11.1 | oxysterol binding protein-like 1A | 4.99916 | 5.32102 | -0.321866 | -1.24995 | 0.0031375 | 0.148505 |
| LYN | chr8q13 | LYN proto-oncogene, Src family  tyrosine kinase | 8.44845 | 8.76959 | -0.321143 | -1.24932 | 0.0050862 | 0.157506 |
| ARRB1 | chr11q13 | arrestin, beta 1 | 5.05805 | 5.34229 | -0.284248 | -1.21777 | 0.011863 | 0.185824 |
| MPP1 | chrXq28 | membrane protein, palmitoylated 1 | 8.01343 | 8.32064 | -0.307204 | -1.23731 | 0.0056794 | 0.158791 |
| RASGRP3 | chr2p25.1-p24.1 | RAS guanyl releasing protein 3 (calcium  and DAG-regulated) | 6.59848 | 6.89061 | -0.292124 | -1.22444 | 0.0089492 | 0.172413 |
| SAT1 | chrXp22.1 | spermidine/spermine N1-  acetyltransferase 1 | 11.5492 | 11.8533 | -0.304034 | -1.23459 | 0.0050921 | 0.157506 |
| FEZ2 | chr2p21 | fasciculation and elongation protein  zeta 2 (zygin II) | 7.13108 | 7.39463 | -0.263545 | -1.20042 | 0.0042304 | 0.152075 |

| RTP4 | chr3q27.3 | receptor (chemosensory) transporter  protein 4 | 6.41391 | 6.81334 | -0.39943 | -1.31899 | 0.0125159 | 0.187104 |
| --- | --- | --- | --- | --- | --- | --- | --- | --- |
| AIM2 | chr1q22 | absent in melanoma 2 | 7.96817 | 8.35966 | -0.391486 | -1.31174 | 0.0008112 | 0.128628 |
| NEXN | chr1p31.1 | nexilin (F actin binding protein) | 5.91935 | 6.27503 | -0.355681 | -1.27959 | 0.0176657 | 0.198559 |
| MS4A6A | chr11q12.1 | membrane-spanning 4-domains,  subfamily A, member 6A | 5.92175 | 6.2439 | -0.322151 | -1.25019 | 0.0164618 | 0.196619 |
| MS4A6A | chr11q12.1 | membrane-spanning 4-domains,  subfamily A, member 6A | 5.92732 | 6.35515 | -0.427826 | -1.3452 | 0.0002226 | 0.111326 |
| SFT2D2 | chr1q24.2 | SFT2 domain containing 2 | 6.22279 | 6.52525 | -0.302456 | -1.23324 | 0.0084562 | 0.170522 |
| BLVRA | chr7p13 | biliverdin reductase A | 7.16972 | 7.52596 | -0.356244 | -1.28009 | 0.0029714 | 0.148407 |
| BLVRA | chr7p13 | biliverdin reductase A | 7.23974 | 7.56415 | -0.324411 | -1.25215 | 0.0022823 | 0.148407 |
| BLVRA | chr7p13 | biliverdin reductase A | 5.85966 | 6.17251 | -0.31285 | -1.24216 | 0.0037919 | 0.150735 |
| --- | --- | --- | 5.99274 | 6.27065 | -0.277913 | -1.21244 | 0.0048598 | 0.156541 |
| ATP1B1 | chr1q24 | ATPase, Na+/K+ transporting, beta 1  polypeptide | 5.91437 | 6.25539 | -0.341011 | -1.26664 | 0.0018312 | 0.145897 |
| MRC1 | chr10p12.33 | mannose receptor, C type 1 | 2.80605 | 3.11441 | -0.308359 | -1.2383 | 0.0045377 | 0.154764 |
| SYNPO2 | chr4q26 | synaptopodin 2 | 3.00187 | 3.27556 | -0.273691 | -1.2089 | 0.0134864 | 0.189772 |
| C1QB | chr1p36.12 | complement component 1, q  subcomponent, B chain | 3.14527 | 3.57907 | -0.433795 | -1.35078 | 0.0003116 | 0.111326 |
| C22orf42 | chr22q12.3 | chromosome 22 open reading frame  42 | 3.65707 | 3.95859 | -0.301524 | -1.23245 | 0.0051572 | 0.157506 |
| TTC6 | chr14q21.1 | tetratricopeptide repeat domain 6 | 1.97073 | 2.23599 | -0.265257 | -1.20185 | 0.0099149 | 0.174971 |
| ZNF628 | chr19q13.42 | zinc finger protein 628 | 4.29211 | 4.57883 | -0.286716 | -1.21986 | 0.0030314 | 0.148407 |
| RNF5 | chr6p21.3 | ring finger protein 5, E3 ubiquitin  protein ligase | 3.53891 | 3.81146 | -0.272545 | -1.20794 | 0.001668 | 0.145076 |
| LIMS1 /// LIMS3 /// LIMS3L | chr2q12.3 /// chr2q13 | LIM and senescent cell antigen-like  domains 1 /// LIM and senescent cell antigen-like domains 3 /// LIM and senescent cell antigen-like domains 3-  like | 3.86873 | 4.21845 | -0.349716 | -1.27431 | 0.0046101 | 0.155277 |
| SLC2A13 | chr12q12 | solute carrier family 2 (facilitated  glucose transporter), member 13 | 3.03356 | 3.35692 | -0.32336 | -1.25124 | 0.0067001 | 0.164139 |
| SGOL2 | chr2q33.1 | shugoshin-like 2 (S. pombe) | 4.51122 | 5.00221 | -0.490991 | -1.40541 | 2.88E-05 | 0.103347 |
| LINC01128 | chr1p36.33 | long intergenic non-protein coding  RNA 1128 | 2.97116 | 3.30152 | -0.330364 | -1.25733 | 0.000935 | 0.128628 |
| --- | --- | --- | 4.62464 | 4.93666 | -0.312014 | -1.24144 | 0.0021367 | 0.147757 |
| ASTN2 | chr9q33.1 | astrotactin 2 | 2.44196 | 2.73595 | -0.293988 | -1.22602 | 0.0123254 | 0.187004 |
| COL25A1 | chr4q25 | collagen, type XXV, alpha 1 | 2.4787 | 2.75933 | -0.280628 | -1.21472 | 0.0096429 | 0.173245 |
| CTDSPL | chr3p21.3 | CTD small phosphatase like | 4.58044 | 4.84587 | -0.265436 | -1.202 | 0.0125734 | 0.187104 |
| SLC12A6 | chr15q13 | solute carrier family 12  (potassium/chloride transporter), member 6 | 6.18514 | 6.4571 | -0.271958 | -1.20745 | 0.0060502 | 0.161186 |
| LOC102546  226 | chr5q14.3 | uncharacterized LOC102546226 | 2.63297 | 2.90958 | -0.276603 | -1.21134 | 0.0043277 | 0.1529 |
| --- | --- | --- | 3.26261 | 3.52747 | -0.264862 | -1.20152 | 0.007678 | 0.168472 |
| BZW1 | chr2q33 | basic leucine zipper and W2 domains 1 | 5.02294 | 5.36221 | -0.339269 | -1.26512 | 0.0080186 | 0.168575 |
| MRAS | chr3q22.3 | muscle RAS oncogene homolog | 5.17835 | 5.4422 | -0.263849 | -1.20068 | 0.0039165 | 0.151256 |
| NUCB2 | chr11p15.1 | nucleobindin 2 | 8.13359 | 8.45224 | -0.318656 | -1.24717 | 0.0012665 | 0.136585 |
| CSRP3 | chr11p15.1 | cysteine and glycine-rich protein 3  (cardiac LIM protein) | 2.6261 | 2.91817 | -0.29207 | -1.2244 | 0.001636 | 0.145076 |
| STARD13 | chr13q13.1 | StAR-related lipid transfer domain  containing 13 | 3.64226 | 3.96465 | -0.322393 | -1.2504 | 0.0002486 | 0.111326 |
| BTD | chr3p25 | biotinidase | 4.56667 | 4.85152 | -0.284857 | -1.21829 | 0.0004376 | 0.112391 |
| CKS1B | chr1q21.2 | CDC28 protein kinase regulatory  subunit 1B | 7.39399 | 7.67672 | -0.282727 | -1.21649 | 0.0010561 | 0.132575 |

| GBP5 | chr1p22.2 | guanylate binding protein 5 | 7.99228 | 8.28532 | -0.293038 | -1.22522 | 0.0008376 | 0.128628 |
| --- | --- | --- | --- | --- | --- | --- | --- | --- |
| TUBB2A | chr6p25 | tubulin, beta 2A class IIa | 6.49429 | 6.772 | -0.277719 | -1.21228 | 0.0037802 | 0.150735 |
| CEP63 | chr3q22.2 | centrosomal protein 63kDa | 5.5658 | 5.8672 | -0.301395 | -1.23234 | 0.0015963 | 0.145076 |
| SETDB2 | chr13q14 | SET domain, bifurcated 2 | 6.73957 | 7.01328 | -0.273713 | -1.20892 | 0.00035 | 0.111326 |
| APOBEC3B | chr22q13.1- q13.2 | apolipoprotein B mRNA editing enzyme, catalytic polypeptide-like 3B | 3.19707 | 3.57373 | -0.376662 | -1.29833 | 0.0001913 | 0.111326 |
| SSB | chr2q31.1 | Sjogren syndrome antigen B  (autoantigen La) | 8.10819 | 8.49453 | -0.386339 | -1.30707 | 3.78E-05 | 0.103347 |
| ECT2 | chr3q26.1-q26.2 | epithelial cell transforming 2 | 4.66339 | 4.97909 | -0.315699 | -1.24461 | 0.0019116 | 0.14618 |
| BRMS1L | chr14q13.2 | breast cancer metastasis-suppressor 1-  like | 3.71645 | 3.99194 | -0.275495 | -1.21041 | 0.003118 | 0.148407 |
| CBWD1 /// CBWD2 /// CBWD3 /// CBWD4P  /// CBWD5  /// CBWD6  /// CBWD7 | chr2q13 /// chr9p12 /// chr9p24.3 /// chr9q13 /// chr9q21.11 | COBW domain containing 1 /// COBW domain containing 2 /// COBW domain containing 3 /// COBW domain containing 4 pseudogene /// COBW domain containing 5 /// COBW domain containing 6 /// COBW domain  containing 7 | 8.04001 | 8.38806 | -0.348046 | -1.27284 | 0.0001518 | 0.111326 |
| CDC42EP3 | chr2p21 | CDC42 effector protein (Rho GTPase  binding) 3 | 7.5204 | 7.85632 | -0.335919 | -1.26218 | 0.0002693 | 0.111326 |
| IFI16 | chr1q22 | interferon, gamma-inducible protein  16 | 9.82651 | 10.1527 | -0.326166 | -1.25368 | 1.437E-05 | 0.103347 |
| IFI16 | chr1q22 | interferon, gamma-inducible protein  16 | 9.53585 | 9.85986 | -0.324007 | -1.2518 | 1.222E-05 | 0.103347 |
| IL15 | chr4q31 | interleukin 15 | 7.2486 | 7.52592 | -0.277318 | -1.21194 | 0.0010404 | 0.13144 |
| RPAP3 | chr12q13.11 | RNA polymerase II associated protein 3 | 8.03427 | 8.35233 | -0.318058 | -1.24665 | 7.016E-05 | 0.111326 |
| CDC73 | chr1q25 | cell division cycle 73 | 7.22002 | 7.48981 | -0.269785 | -1.20563 | 0.001951 | 0.146411 |
| TMX1 | chr14q22.1 | thioredoxin-related transmembrane  protein 1 | 8.88885 | 9.21197 | -0.323117 | -1.25103 | 0.0001381 | 0.111326 |
| ANXA1 | chr9q21.13 | annexin A1 | 11.0451 | 11.3105 | -0.26536 | -1.20194 | 0.0002233 | 0.111326 |
| GNL3 | chr3p21.1 | guanine nucleotide binding protein-  like 3 (nucleolar) | 4.30048 | 4.59709 | -0.296609 | -1.22825 | 0.0013019 | 0.137103 |
| UQCRB | chr8q22 | ubiquinol-cytochrome c reductase  binding protein | 3.35419 | 3.62858 | -0.274382 | -1.20948 | 9.585E-06 | 0.103347 |
| --- | --- | --- | 2.01651 | 2.28023 | -0.263717 | -1.20057 | 0.0023044 | 0.148407 |
| VPS13C | chr15q22.2 | vacuolar protein sorting 13 homolog C  (S. cerevisiae) | 4.44276 | 4.76902 | -0.326259 | -1.25376 | 0.006421 | 0.163699 |
| DDX6 | chr11q23.3 | DEAD (Asp-Glu-Ala-Asp) box helicase 6 | 5.38601 | 5.71328 | -0.327263 | -1.25463 | 0.0024387 | 0.148407 |
| CCDC13 | chr3p22.1 | coiled-coil domain containing 13 | 4.50587 | 4.81065 | -0.304777 | -1.23523 | 0.0042101 | 0.151955 |
| --- | --- | --- | 5.02124 | 5.31019 | -0.288948 | -1.22175 | 0.006205 | 0.161814 |
| CTTN | chr11q13 | cortactin | 3.63173 | 3.91207 | -0.280341 | -1.21448 | 0.0106251 | 0.180552 |
| LOC101927  768 | chr6q22.1 | uncharacterized LOC101927768 | 3.06328 | 3.37294 | -0.309667 | -1.23942 | 0.002862 | 0.148407 |
| FGFR1 | chr8p11.23-  p11.22 | fibroblast growth factor receptor 1 | 4.49693 | 4.86695 | -0.370019 | -1.29237 | 0.0006083 | 0.128628 |
| CCNE2 | chr8q22.1 | cyclin E2 | 3.52258 | 3.81955 | -0.29697 | -1.22856 | 0.0056765 | 0.158791 |
| RPS10P2 | chr20p12 | ribosomal protein S10 pseudogene 2 | 5.67938 | 6.03835 | -0.358974 | -1.28251 | 0.0019026 | 0.14618 |

| RPS17P5 | chr6p12.3 | ribosomal protein S17 pseudogene 5 | 7.78139 | 8.07662 | -0.29523 | -1.22708 | 0.0053569 | 0.158323 |
| --- | --- | --- | --- | --- | --- | --- | --- | --- |
| --- | --- | --- | 6.36637 | 6.64421 | -0.277839 | -1.21238 | 0.0034045 | 0.150538 |
| PPIG | chr2q31.1 | peptidylprolyl isomerase G (cyclophilin  G) | 8.03456 | 8.31661 | -0.282048 | -1.21592 | 0.004311 | 0.152737 |
| GIMAP7 | chr7q36.1 | GTPase, IMAP family member 7 | 9.67203 | 9.93832 | -0.26629 | -1.20271 | 0.0098959 | 0.174971 |
| --- | --- | --- | 3.35188 | 3.64909 | -0.297209 | -1.22876 | 0.0036694 | 0.150735 |
| --- | --- | --- | 2.93307 | 3.20585 | -0.27278 | -1.20813 | 0.0011446 | 0.134826 |
| RECQL4 | chr8q24.3 | RecQ helicase-like 4 | 4.90135 | 5.19908 | -0.297737 | -1.22921 | 0.0013952 | 0.138618 |
| PDGFRL | chr8p22-p21.3 | platelet-derived growth factor  receptor-like | 2.97184 | 3.26873 | -0.296896 | -1.2285 | 0.0027881 | 0.148407 |
| LARP1B | chr4q28.2 | La ribonucleoprotein domain family,  member 1B | 3.15258 | 3.42369 | -0.271106 | -1.20673 | 0.0040055 | 0.151256 |
| PAX9 | chr14q13.3 | paired box 9 | 2.861 | 3.15929 | -0.298285 | -1.22968 | 0.0041371 | 0.151685 |
| CCNG2 | chr4q21.1 | cyclin G2 | 3.25597 | 3.52157 | -0.265602 | -1.20214 | 0.0109351 | 0.181825 |
| BANK1 | chr4q24 | B-cell scaffold protein with ankyrin  repeats 1 | 7.14368 | 7.44513 | -0.301455 | -1.23239 | 0.0123647 | 0.187004 |
| --- | --- | --- | 2.80976 | 3.08969 | -0.279933 | -1.21414 | 0.016899 | 0.197956 |
| --- | --- | --- | 3.9841 | 4.27165 | -0.287549 | -1.22056 | 0.0071932 | 0.167056 |
| NAT8B | chr2p13.1 | N-acetyltransferase 8B (GCN5-related, putative, gene/pseudogene) | 4.37801 | 4.65648 | -0.27847 | -1.21291 | 0.0108313 | 0.181472 |
| --- | --- | --- | 3.49866 | 3.78143 | -0.282772 | -1.21653 | 0.0089244 | 0.172413 |
| KCNIP4 | chr4p15.32 | Kv channel interacting protein 4 | 4.19201 | 4.45521 | -0.263202 | -1.20014 | 0.0036707 | 0.150735 |
| MS4A4A | chr11q12 | membrane-spanning 4-domains,  subfamily A, member 4A | 3.98008 | 4.32196 | -0.341877 | -1.2674 | 0.0165415 | 0.196619 |
| RNMT | chr18p11.21 | RNA (guanine-7-) methyltransferase | 5.4429 | 5.77211 | -0.329209 | -1.25632 | 0.0163357 | 0.196109 |
| GPAT3 | chr4q21.23 | glycerol-3-phosphate acyltransferase 3 | 4.90263 | 5.21714 | -0.314507 | -1.24359 | 0.0034254 | 0.150538 |
| ARL15 | chr5p15.2 | ADP-ribosylation factor like GTPase 15 | 3.34244 | 3.64318 | -0.300739 | -1.23178 | 0.0050646 | 0.157506 |
| CXCR2 | chr2q35 | chemokine (C-X-C motif) receptor 2 | 3.13231 | 3.39873 | -0.266426 | -1.20282 | 0.0148282 | 0.192303 |
| RNASE4 | chr14q11 | ribonuclease, RNase A family, 4 | 3.48533 | 3.7882 | -0.302874 | -1.2336 | 0.0098749 | 0.174971 |
| WBP5 | chrXq22.2 | WW domain binding protein 5 | 4.79284 | 5.17643 | -0.383594 | -1.30459 | 0.0018821 | 0.14618 |
| OPN3 | chr1q43 | opsin 3 | 4.05737 | 4.40081 | -0.343442 | -1.26878 | 0.0099813 | 0.175177 |
| CALD1 | chr7q33 | caldesmon 1 | 4.51381 | 4.82839 | -0.314586 | -1.24365 | 0.0136722 | 0.190437 |
| TRIQK | chr8q22.1 | triple QxxK/R motif containing | 4.38258 | 4.75094 | -0.368363 | -1.29089 | 0.0053822 | 0.158461 |
| ARMCX1 | chrXq22.1 | armadillo repeat containing, X-linked 1 | 4.85429 | 5.17976 | -0.325464 | -1.25307 | 0.0158372 | 0.194858 |
| SAT1 | chrXp22.1 | spermidine/spermine N1-  acetyltransferase 1 | 11.0555 | 11.4568 | -0.401243 | -1.32065 | 0.000398 | 0.111326 |
| TFEC | chr7q31.2 | transcription factor EC | 2.23207 | 2.57518 | -0.343105 | -1.26848 | 0.0077103 | 0.168472 |
| FAM45A  /// FAM45B | chr10q25 /// chrXq26.1 | family with sequence similarity 45, member A /// family with sequence similarity 45, member A pseudogene | 5.97661 | 6.29409 | -0.317483 | -1.24615 | 0.0055168 | 0.158791 |
| LOC100996  517 /// LOC100996  716 /// LOC102724  364 ///  SEC22B | chr1q21.1 | vesicle-trafficking protein SEC22b-like  /// vesicle-trafficking protein SEC22b- like /// vesicle-trafficking protein SEC22b-like /// SEC22 homolog B, vesicle trafficking protein  (gene/pseudogene) | 7.19067 | 7.49648 | -0.30581 | -1.23611 | 0.0056212 | 0.158791 |
| QKI | chr6q26 | QKI, KH domain containing, RNA  binding | 8.23948 | 8.52591 | -0.28643 | -1.21962 | 0.0168343 | 0.197836 |

| ALG14 | chr1p21.3 | ALG14, UDP-N-  acetylglucosaminyltransferase subunit | 5.37579 | 5.73917 | -0.363375 | -1.28643 | 0.0010049 | 0.129126 |
| --- | --- | --- | --- | --- | --- | --- | --- | --- |
| ME1 | chr6q12 | malic enzyme 1, NADP(+)-dependent,  cytosolic | 6.14352 | 6.40699 | -0.263463 | -1.20036 | 0.0062768 | 0.162624 |
| MALAT1 | chr11q13.1 | metastasis associated lung  adenocarcinoma transcript 1 (non- protein coding) | 11.1845 | 11.6349 | -0.450382 | -1.3664 | 0.0002326 | 0.111326 |
| BBS7 | chr4q27 | Bardet-Biedl syndrome 7 | 4.80637 | 5.11464 | -0.308273 | -1.23822 | 0.0093037 | 0.173008 |
| MRPL50 | chr9q31.1 | mitochondrial ribosomal protein L50 | 6.7055 | 7.0133 | -0.307793 | -1.23781 | 0.0133893 | 0.189772 |
| PPP1CB | chr2p23 | protein phosphatase 1, catalytic  subunit, beta isozyme | 8.02751 | 8.31965 | -0.292142 | -1.22446 | 0.011048 | 0.181975 |
| EIF1AX | chrXp22.12 | eukaryotic translation initiation factor  1A, X-linked | 6.54967 | 6.87417 | -0.324498 | -1.25223 | 0.0060222 | 0.161186 |
| EIF4E | chr4q23 | eukaryotic translation initiation factor  4E | 7.21974 | 7.5524 | -0.332662 | -1.25933 | 0.0030774 | 0.148407 |
| EIF1AY | chrYq11.223 | eukaryotic translation initiation factor  1A, Y-linked | 6.787 | 7.06804 | -0.281042 | -1.21507 | 0.0093268 | 0.173053 |
| EID1 | chr15q21.1 | EP300 interacting inhibitor of  differentiation 1 | 6.15488 | 6.45558 | -0.300693 | -1.23174 | 0.0008178 | 0.128628 |
| C3orf38 | chr3p11.1 | chromosome 3 open reading frame 38 | 7.53385 | 7.85058 | -0.316733 | -1.24551 | 0.0045865 | 0.155277 |
| ZNF92 | chr7q11.21 | zinc finger protein 92 | 7.5653 | 7.87042 | -0.305119 | -1.23552 | 0.0022074 | 0.148212 |
| DCAF4L1 | chr4p13 | DDB1 and CUL4 associated factor 4-like  1 | 2.45817 | 2.73789 | -0.279716 | -1.21396 | 0.0057169 | 0.158791 |
| PF4V1 | chr4q12-q21 | platelet factor 4 variant 1 | 5.2849 | 5.67405 | -0.389151 | -1.30962 | 0.0076366 | 0.168472 |
| ZNF292 | chr6q14.3 | zinc finger protein 292 | 7.39637 | 7.6695 | -0.273128 | -1.20842 | 0.0068203 | 0.164807 |
| BANK1 | chr4q24 | B-cell scaffold protein with ankyrin  repeats 1 | 7.56568 | 7.833 | -0.267325 | -1.20357 | 0.0080452 | 0.168682 |
| CARD16 ///  CASP1 | chr11q22 ///  chr11q23 | caspase recruitment domain family,  member 16 /// caspase 1 | 8.68102 | 9.00232 | -0.321295 | -1.24945 | 0.0010125 | 0.129598 |
| RPL31 /// TBC1D8 | chr2q11.2 | ribosomal protein L31 /// TBC1 domain family, member 8 (with GRAM domain) | 3.27727 | 3.5886 | -0.31133 | -1.24085 | 0.0016018 | 0.145076 |
| CD1D | chr1q23.1 | CD1d molecule | 4.15247 | 4.44995 | -0.297476 | -1.22899 | 0.0139475 | 0.190686 |
| PAPSS2 | chr10q24 | 3'-phosphoadenosine 5'-  phosphosulfate synthase 2 | 3.39775 | 3.69199 | -0.294232 | -1.22623 | 0.0045631 | 0.155277 |
| ANKRD28 | chr3p25.1 | ankyrin repeat domain 28 | 5.64078 | 5.95241 | -0.311623 | -1.2411 | 0.0004021 | 0.111326 |
| ATP2B1 | chr12q21.3 | ATPase, Ca++ transporting, plasma  membrane 1 | 7.51678 | 7.80992 | -0.29314 | -1.2253 | 0.0007454 | 0.128628 |
| THAP2 | chr12q21.1 | THAP domain containing, apoptosis  associated protein 2 | 3.58236 | 3.88874 | -0.306383 | -1.2366 | 0.0042982 | 0.152719 |
| NUP58 | chr13q12.13 | nucleoporin 58kDa | 4.18117 | 4.47536 | -0.294192 | -1.2262 | 0.0019881 | 0.146443 |
| ARHGAP5 | chr14q12 | Rho GTPase activating protein 5 | 5.52145 | 5.79005 | -0.268603 | -1.20464 | 0.0010261 | 0.130062 |
| GLS | chr2q32-q34 | glutaminase | 8.959 | 9.26309 | -0.304083 | -1.23463 | 0.0055318 | 0.158791 |
| ZNF131 | chr5p12 | zinc finger protein 131 | 5.74033 | 6.00733 | -0.266993 | -1.2033 | 0.0026981 | 0.148407 |
| GCA | chr2q24.2 | grancalcin, EF-hand calcium binding  protein | 6.2821 | 6.70354 | -0.421438 | -1.33926 | 2.701E-05 | 0.103347 |
| RNF219 | chr13q31.1 | ring finger protein 219 | 3.7324 | 4.11764 | -0.385241 | -1.30608 | 0.0003928 | 0.111326 |
| MARCH1 | chr4q32.2 | membrane associated ring finger 1 | 6.99692 | 7.48032 | -0.483398 | -1.39803 | 0.0004248 | 0.112391 |
| MARCH1 | chr4q32.2 | membrane associated ring finger 1 | 7.49374 | 7.97666 | -0.482913 | -1.39756 | 0.0008328 | 0.128628 |
| FAM26F | chr6q22.1 | family with sequence similarity 26,  member F | 6.63871 | 7.09968 | -0.46097 | -1.37647 | 0.0018743 | 0.14618 |
| UBE2D1 | chr10q21.1 | ubiquitin conjugating enzyme E2D 1 | 8.01949 | 8.44366 | -0.424176 | -1.34181 | 0.0005071 | 0.122884 |

| UBE2D1 | chr10q21.1 | ubiquitin conjugating enzyme E2D 1 | 5.30606 | 5.70379 | -0.397734 | -1.31744 | 0.0029906 | 0.148407 |
| --- | --- | --- | --- | --- | --- | --- | --- | --- |
| CHMP5 | chr9p13.3 | charged multivesicular body protein 5 | 8.52008 | 8.89394 | -0.373858 | -1.29581 | 0.0021906 | 0.148212 |
| BLZF1 | chr1q24 | basic leucine zipper nuclear factor 1 | 4.74121 | 5.17024 | -0.429031 | -1.34633 | 0.0009713 | 0.129126 |
| ZEB2 | chr2q22.3 | zinc finger E-box binding homeobox 2 | 8.20066 | 8.69967 | -0.499006 | -1.41324 | 7.316E-05 | 0.111326 |
| BLZF1 | chr1q24 | basic leucine zipper nuclear factor 1 | 3.47331 | 3.89931 | -0.426004 | -1.34351 | 0.0041494 | 0.151685 |
| FRMD3 | chr9q21.32 | FERM domain containing 3 | 4.16662 | 4.44648 | -0.279858 | -1.21408 | 0.0145224 | 0.191711 |
| ZBED6 | chr1q32.1 | zinc finger, BED-type containing 6 | 6.53346 | 6.91007 | -0.376609 | -1.29829 | 0.0026118 | 0.148407 |
| DENND1B | chr1q31.3 | DENN/MADD domain containing 1B | 6.24312 | 6.64721 | -0.404086 | -1.32325 | 0.0002511 | 0.111326 |
| STAG3L4 | chr7p11.2-q11.2 | stromal antigen 3-like 4 (pseudogene) | 3.90902 | 4.20461 | -0.29559 | -1.22739 | 0.0020653 | 0.147757 |
| FAM72A  /// FAM72B  /// FAM72C  /// FAM72D | chr1p11.2 /// chr1q21.1 /// chr1q32.1 | family with sequence similarity 72, member A /// family with sequence similarity 72, member B /// family with sequence similarity 72, member C /// family with sequence similarity 72,  member D | 5.3013 | 5.72448 | -0.423176 | -1.34088 | 0.0016114 | 0.145076 |
| MAP3K7CL | chr21q22.3 | MAP3K7 C-terminal like | 6.91958 | 7.32899 | -0.409408 | -1.32814 | 0.0024759 | 0.148407 |
| SGOL2 | chr2q33.1 | shugoshin-like 2 (S. pombe) | 5.29869 | 5.76514 | -0.466455 | -1.38171 | 0.0001972 | 0.111326 |
| CLECL1 | chr12p13.31 | C-type lectin-like 1 | 5.44972 | 5.92824 | -0.478521 | -1.39331 | 0.0002793 | 0.111326 |
| FAM26F | chr6q22.1 | family with sequence similarity 26,  member F | 6.48787 | 6.92305 | -0.435177 | -1.35208 | 0.0004695 | 0.118618 |
| NXT2 | chrXq23 | nuclear transport factor 2-like export  factor 2 | 6.12226 | 6.5022 | -0.379945 | -1.30129 | 0.0036578 | 0.150674 |
| CLIC2 | chrXq28 | chloride intracellular channel 2 | 5.35378 | 5.74219 | -0.388412 | -1.30895 | 0.0058184 | 0.160158 |
| HIST1H2BC | chr6p22.1 | histone cluster 1, H2bc | 4.30581 | 4.65102 | -0.345218 | -1.27034 | 0.0042111 | 0.151955 |
| BCL2A1 | chr15q24.3 | BCL2-related protein A1 | 7.80383 | 8.14692 | -0.343086 | -1.26847 | 0.00798 | 0.168566 |
| LINC00537 | chr9q21.11 | long intergenic non-protein coding  RNA 537 | 5.68351 | 5.98134 | -0.297826 | -1.22929 | 0.0076725 | 0.168472 |
| RNASEH2C | chr11q13.1 | ribonuclease H2, subunit C | 6.9499 | 7.24348 | -0.293579 | -1.22568 | 0.0078137 | 0.168472 |
| GPR183 | chr13q32.3 | G protein-coupled receptor 183 | 9.78047 | 10.078 | -0.297546 | -1.22905 | 0.0040076 | 0.151256 |
| PSMA4 | chr15q25.1 | proteasome subunit alpha 4 | 9.78099 | 10.0651 | -0.284079 | -1.21763 | 0.0046076 | 0.155277 |
| --- | --- | --- | 2.53821 | 2.84006 | -0.301857 | -1.23273 | 0.0070937 | 0.166748 |
| PTRH2 | chr17q23.1 | peptidyl-tRNA hydrolase 2 | 7.3712 | 7.69966 | -0.328455 | -1.25567 | 0.0036829 | 0.150735 |
| TXNDC17 | chr17p13.1 | thioredoxin domain containing 17 | 7.04219 | 7.31932 | -0.27713 | -1.21178 | 0.0095187 | 0.173123 |
| INHBA | chr7p15-p13 | inhibin beta A | 3.51225 | 3.89114 | -0.378884 | -1.30034 | 0.0025818 | 0.148407 |
| KBTBD8 | chr3p14 | kelch repeat and BTB (POZ) domain  containing 8 | 6.60294 | 6.94208 | -0.339138 | -1.265 | 0.0031975 | 0.149592 |
| LOC728715  /// OVOS  /// OVOS2 | chr12p11.21 /// chr12p13 /// chr12p13.31 | ovostatin homolog 2 /// ovostatin /// ovostatin 2 | 6.38758 | 6.69197 | -0.30439 | -1.2349 | 0.0099144 | 0.174971 |
| DPH3 | chr3p25.1 | diphthamide biosynthesis 3 | 7.06891 | 7.34699 | -0.278076 | -1.21258 | 0.0132693 | 0.189533 |
| LOC105371  967 | --- | uncharacterized LOC105371967 | 3.7029 | 3.97621 | -0.273311 | -1.20858 | 0.0113171 | 0.182872 |
| STXBP5 | chr6q24.3 | syntaxin binding protein 5 (tomosyn) | 4.59075 | 4.874 | -0.283247 | -1.21693 | 0.0114804 | 0.18387 |
| --- | --- | --- | 3.31085 | 3.61489 | -0.304041 | -1.2346 | 0.000266 | 0.111326 |
| MYLK | chr3q21 | myosin light chain kinase | 5.22303 | 5.49797 | -0.27494 | -1.20994 | 0.0150919 | 0.193396 |

| --- | --- | --- | 4.4988 | 4.76325 | -0.264446 | -1.20117 | 0.0160139 | 0.195587 |
| --- | --- | --- | --- | --- | --- | --- | --- | --- |
| IPO11 ///  LRRC70 | chr5q12.1 | importin 11 /// leucine rich repeat  containing 70 | 3.11932 | 3.42958 | -0.31026 | -1.23993 | 0.0056483 | 0.158791 |
| GUCY1B3 | chr4q31.3-q33 | guanylate cyclase 1, soluble, beta 3 | 5.01332 | 5.31715 | -0.303834 | -1.23442 | 0.0067678 | 0.164602 |
| PLEK | chr2p13.3 | pleckstrin | 8.01542 | 8.2834 | -0.267979 | -1.20412 | 0.0030956 | 0.148407 |
| KIAA1524 | chr3q13.13 | KIAA1524 | 3.24181 | 3.52127 | -0.279451 | -1.21373 | 0.0021727 | 0.147979 |
| UBR3 | chr2q31.1 | ubiquitin protein ligase E3 component  n-recognin 3 (putative) | 2.7831 | 3.05505 | -0.271946 | -1.20744 | 0.0017587 | 0.145076 |
| TAL1 | chr1p32 | T-cell acute lymphocytic leukemia 1 | 4.71991 | 5.02722 | -0.30731 | -1.2374 | 0.0019818 | 0.146411 |
| ZHX1 | chr8q24.13 | zinc fingers and homeoboxes 1 | 5.8066 | 6.09157 | -0.284969 | -1.21838 | 0.002745 | 0.148407 |
| CACNA1A | chr19p13 | calcium channel, voltage-dependent, P/Q type, alpha 1A subunit | 5.1365 | 5.40408 | -0.26758 | -1.20379 | 0.0019977 | 0.146595 |
| LINC00550 | chr13q21.33 | long intergenic non-protein coding  RNA 550 | 2.16456 | 2.43415 | -0.269587 | -1.20546 | 0.0135613 | 0.189966 |
| LOC101927  402 | --- | uncharacterized proline-rich protein-  like | 3.22589 | 3.51927 | -0.293374 | -1.2255 | 0.0019836 | 0.146411 |
| NDC80 | chr18p11.32 | NDC80 kinetochore complex  component | 4.62874 | 4.92618 | -0.297448 | -1.22897 | 0.0017336 | 0.145076 |
| CYSLTR1 | chrXq13.2-q21.1 | cysteinyl leukotriene receptor 1 | 5.79303 | 6.07574 | -0.282706 | -1.21647 | 0.0085557 | 0.170624 |
| HDAC2 | chr6q21 | histone deacetylase 2 | 8.30059 | 8.63301 | -0.332421 | -1.25912 | 4.5E-05 | 0.103347 |
| HCFC2 | chr12q23.3 | host cell factor C2 | 6.80516 | 7.0891 | -0.283942 | -1.21752 | 1.975E-05 | 0.103347 |
| GLIPR1 | chr12q21.2 | GLI pathogenesis-related 1 | 6.90305 | 7.18679 | -0.283738 | -1.21734 | 0.0001341 | 0.111326 |
| TCF4 | chr18q21.1 | transcription factor 4 | 6.9552 | 7.22448 | -0.26928 | -1.20521 | 0.0008251 | 0.128628 |
| PRPF4B | chr6p25.2 | pre-mRNA processing factor 4B | 8.91149 | 9.18261 | -0.271122 | -1.20675 | 0.0003611 | 0.111326 |
| HSP90AA1 | chr14q32.33 | heat shock protein 90kDa alpha  (cytosolic), class A member 1 | 11.2772 | 11.546 | -0.268809 | -1.20481 | 0.0012622 | 0.136585 |
| NUDCD1 | chr8q23 | NudC domain containing 1 | 6.65949 | 6.92724 | -0.267747 | -1.20393 | 0.0103275 | 0.178244 |
| PNPLA8 | chr7q31 | patatin-like phospholipase domain  containing 8 | 8.92527 | 9.19843 | -0.273156 | -1.20845 | 0.0024559 | 0.148407 |
| STAP1 | chr4q13.2 | signal transducing adaptor family  member 1 | 6.66354 | 6.92734 | -0.263791 | -1.20063 | 0.0056679 | 0.158791 |
| TCEB1 | chr8q21.11 | transcription elongation factor B (SIII), polypeptide 1 (15kDa, elongin C) | 6.00278 | 6.28886 | -0.286078 | -1.21932 | 0.0093243 | 0.173053 |
| USP38 | --- | ubiquitin specific peptidase 38 | 4.50035 | 4.78166 | -0.281314 | -1.2153 | 0.0133248 | 0.189716 |
| ASRGL1 | chr11q12.3 | asparaginase like 1 | 4.21824 | 4.48132 | -0.263082 | -1.20004 | 0.0178112 | 0.198662 |
| EIF4G2 | chr11p15 | eukaryotic translation initiation factor  4 gamma, 2 | 4.0183 | 3.69711 | 0.321189 | 1.24936 | 0.0118401 | 0.185824 |
| --- | --- | --- | 3.31364 | 2.91742 | 0.396216 | 1.31605 | 0.0144595 | 0.191711 |
| GPN2 | chr1p36.11 | GPN-loop GTPase 2 | 6.32502 | 5.97749 | 0.347531 | 1.27238 | 0.0134697 | 0.189772 |
| --- | --- | --- | 4.15932 | 3.79704 | 0.362274 | 1.28545 | 0.0120586 | 0.186203 |
| ABLIM1 | chr10q25 | actin binding LIM protein 1 | 8.72763 | 8.37883 | 0.348796 | 1.2735 | 0.0118645 | 0.185824 |
| ZBTB20 | chr3q13.2 | zinc finger and BTB domain containing  20 | 4.93136 | 4.47463 | 0.456725 | 1.37242 | 0.006473 | 0.163699 |
| DUSP4 | chr8p12-p11 | dual specificity phosphatase 4 | 6.82605 | 6.47022 | 0.355831 | 1.27972 | 0.0111194 | 0.182294 |
| DUSP4 | chr8p12-p11 | dual specificity phosphatase 4 | 7.47838 | 7.10195 | 0.376429 | 1.29812 | 0.0068308 | 0.164807 |
| NMT2 | chr10p13 | N-myristoyltransferase 2 | 5.47546 | 5.10593 | 0.369532 | 1.29193 | 0.006545 | 0.163699 |
| PLPP6 | chr9p24.1 | phospholipid phosphatase 6 | 5.37856 | 5.00322 | 0.37534 | 1.29715 | 0.0086306 | 0.170754 |
| --- | --- | --- | 5.56807 | 5.28512 | 0.282944 | 1.21668 | 0.0044667 | 0.153894 |
| SORL1 | chr11q23.2-  q24.2 | sortilin-related receptor, L(DLR class) A  repeats containing | 10.3323 | 10.0609 | 0.271462 | 1.20703 | 0.0112707 | 0.182729 |

| PIK3IP1 | chr22q12.2 | phosphoinositide-3-kinase interacting  protein 1 | 10.1455 | 9.86462 | 0.280876 | 1.21493 | 0.0055534 | 0.158791 |
| --- | --- | --- | --- | --- | --- | --- | --- | --- |
| MRPS31P5  /// THSD1 | chr13q /// chr13q14.3 | mitochondrial ribosomal protein S31 pseudogene 5 /// thrombospondin type 1 domain containing 1 | 6.55705 | 6.26778 | 0.289275 | 1.22203 | 0.0173088 | 0.198289 |
| RAP1A | chr1p13.3 | RAP1A, member of RAS oncogene  family | 6.40155 | 6.10512 | 0.296422 | 1.22809 | 0.0054584 | 0.158789 |
| OSGEPL1 | chr2q32.2 | O-sialoglycoprotein endopeptidase-like  1 | 5.45827 | 5.1592 | 0.299076 | 1.23036 | 0.0173665 | 0.1983 |
| --- | --- | --- | 5.68704 | 5.28762 | 0.399416 | 1.31897 | 0.0033644 | 0.150398 |
| COG7 | chr16p12.2 | component of oligomeric golgi  complex 7 | 5.49327 | 5.22876 | 0.264505 | 1.20122 | 0.0131123 | 0.188616 |
| NTMT1 | chr9q34.11 | N-terminal Xaa-Pro-Lys N-  methyltransferase 1 | 4.57387 | 4.3048 | 0.269071 | 1.20503 | 0.0138209 | 0.190686 |
| SUGP2 | chr19p12 | SURP and G-patch domain containing 2 | 5.85887 | 5.52388 | 0.334986 | 1.26136 | 0.0106452 | 0.180552 |
| FMNL1 | chr17q21 | formin like 1 | 7.16588 | 6.89845 | 0.267431 | 1.20366 | 0.0166139 | 0.196733 |
| LONP2 | chr16q12.1 | lon peptidase 2, peroxisomal | 6.19362 | 5.84973 | 0.343892 | 1.26918 | 0.0114175 | 0.183507 |
| LONP2 | chr16q12.1 | lon peptidase 2, peroxisomal | 6.61432 | 6.25518 | 0.359135 | 1.28266 | 0.0054624 | 0.158789 |
| KBTBD6 | chr13q14.11 | kelch repeat and BTB (POZ) domain  containing 6 | 4.35087 | 4.04165 | 0.309226 | 1.23904 | 0.0116331 | 0.185171 |
| NMT2 | chr10p13 | N-myristoyltransferase 2 | 5.96658 | 5.59077 | 0.375803 | 1.29756 | 0.0022437 | 0.148212 |
| SSH2 | chr17q11.2 | slingshot protein phosphatase 2 | 4.50947 | 4.13112 | 0.378351 | 1.29985 | 0.0012204 | 0.136585 |
| --- | --- | --- | 4.70315 | 4.41117 | 0.291979 | 1.22432 | 0.0150708 | 0.193396 |
| --- | --- | --- | 6.70347 | 6.41148 | 0.291986 | 1.22432 | 0.0116092 | 0.185171 |
| SS18 | chr18q11.2 | synovial sarcoma translocation,  chromosome 18 | 7.9023 | 7.58558 | 0.316723 | 1.2455 | 0.0175095 | 0.198559 |
| UVSSA | chr4p16.3 | UV stimulated scaffold protein A | 6.90032 | 6.61298 | 0.287338 | 1.22039 | 0.0129482 | 0.18773 |
| PPA2 | chr4q25 | pyrophosphatase (inorganic) 2 | 5.90924 | 5.61755 | 0.291694 | 1.22408 | 0.0077978 | 0.168472 |
| MRPS11 | chr15q25 | mitochondrial ribosomal protein S11 | 4.7595 | 4.478 | 0.2815 | 1.21546 | 0.0041427 | 0.151685 |
| --- | --- | --- | 5.99521 | 5.70218 | 0.293024 | 1.22521 | 0.007591 | 0.168472 |
| LOC729732 | chr8p23.1 | uncharacterized LOC729732 | 6.46871 | 6.10933 | 0.359377 | 1.28287 | 0.0006647 | 0.128628 |
| --- | --- | --- | 6.63353 | 6.26687 | 0.366659 | 1.28936 | 0.0005537 | 0.124545 |
| ZBTB20 | chr3q13.2 | zinc finger and BTB domain containing  20 | 7.16264 | 6.78933 | 0.373308 | 1.29532 | 0.0028526 | 0.148407 |
| MTERF4 | chr2q37.3 | mitochondrial transcription  termination factor 4 | 7.45829 | 7.18404 | 0.274247 | 1.20936 | 0.00838 | 0.170128 |
| MLLT3 | chr9p22 | myeloid/lymphoid or mixed-lineage leukemia; translocated to, 3 | 6.68666 | 6.41161 | 0.275051 | 1.21004 | 0.003367 | 0.150398 |
| LCP2 | chr5q35.1 | lymphocyte cytosolic protein 2 | 6.7404 | 6.43529 | 0.305105 | 1.23551 | 0.0030029 | 0.148407 |
| TSEN2 | chr3p25.2 | TSEN2 tRNA splicing endonuclease  subunit | 4.8051 | 4.51858 | 0.286516 | 1.21969 | 0.0045971 | 0.155277 |
| CYB561D2 | chr3p21.3 | cytochrome b561 family, member D2 | 5.20757 | 4.86295 | 0.344623 | 1.26982 | 0.0025194 | 0.148407 |
| MZF1 | chr19q13.4 | myeloid zinc finger 1 | 4.84656 | 4.57989 | 0.266676 | 1.20303 | 0.0086969 | 0.17096 |
| ZNF37A | chr10p11.2 | zinc finger protein 37A | 4.21888 | 3.94422 | 0.274657 | 1.20971 | 0.0050334 | 0.157506 |
| --- | --- | --- | 5.06802 | 4.77185 | 0.296172 | 1.22788 | 0.0140304 | 0.190686 |
| --- | --- | --- | 6.5791 | 6.23707 | 0.342026 | 1.26754 | 0.0037215 | 0.150735 |
| CRYZL1 | chr21q21.3 | crystallin zeta like 1 | 5.95883 | 5.67968 | 0.279144 | 1.21347 | 0.0075486 | 0.168472 |
| CEP68 | chr2p14 | centrosomal protein 68kDa | 5.5642 | 5.22114 | 0.343064 | 1.26845 | 0.0028968 | 0.148407 |
| ZNF641 | chr12q13.11 | zinc finger protein 641 | 6.40999 | 6.14683 | 0.263166 | 1.20011 | 0.000249 | 0.111326 |
| --- | --- | --- | 5.8448 | 5.54508 | 0.299724 | 1.23091 | 0.000692 | 0.128628 |

| ZBTB20 | chr3q13.2 | zinc finger and BTB domain containing  20 | 7.90046 | 7.62363 | 0.276831 | 1.21153 | 0.0037257 | 0.150735 |
| --- | --- | --- | --- | --- | --- | --- | --- | --- |
| ZBTB20 | chr3q13.2 | zinc finger and BTB domain containing  20 | 6.82126 | 6.48559 | 0.335671 | 1.26196 | 0.0069573 | 0.165836 |
| GALNT10 | chr5q33.2 | polypeptide N- acetylgalactosaminyltransferase 10 | 6.80428 | 6.45126 | 0.353016 | 1.27723 | 0.0039892 | 0.151256 |
| CCDC18-  AS1 | chr1p22.1 | CCDC18 antisense RNA 1 | 7.42118 | 7.05575 | 0.365429 | 1.28826 | 0.0019517 | 0.146411 |
| EML4 | chr2p21 | echinoderm microtubule associated  protein like 4 | 7.51891 | 7.12861 | 0.390294 | 1.31066 | 0.0004369 | 0.112391 |
| LOC339988 | chr4p16.1 | uncharacterized LOC339988 | 6.45912 | 6.17143 | 0.287694 | 1.22069 | 0.0057228 | 0.158791 |
| IL2RB | chr22q13.1 | interleukin 2 receptor, beta | 8.75797 | 8.46321 | 0.294756 | 1.22668 | 0.0176182 | 0.198559 |
| TBC1D22A | chr22q13.3 | TBC1 domain family, member 22A | 6.52896 | 6.21232 | 0.316639 | 1.24543 | 0.0039862 | 0.151256 |
| CD27 | chr12p13 | CD27 molecule | 8.46946 | 8.19444 | 0.275018 | 1.21001 | 0.0097846 | 0.174094 |
| SPOCK2 | chr10pter-q25.3 | sparc/osteonectin, cwcv and kazal-like domains proteoglycan (testican) 2 | 9.33168 | 9.04307 | 0.288607 | 1.22146 | 0.0147685 | 0.192002 |
| SEPT9 | chr17q25 | septin 9 | 10.3391 | 10.0148 | 0.324205 | 1.25197 | 0.0025365 | 0.148407 |
| SRM | chr1p36-p22 | spermidine synthase | 6.85487 | 6.59013 | 0.264749 | 1.20143 | 0.0146907 | 0.191916 |
| UBTF | chr17q21.31 | upstream binding transcription factor,  RNA polymerase I | 6.04318 | 5.77178 | 0.271398 | 1.20698 | 0.0049809 | 0.157506 |
| PRR5L | chr11p13-p12 | proline rich 5 like | 7.06482 | 6.77471 | 0.290108 | 1.22273 | 0.0088513 | 0.172413 |
| LOC285812 | chr6p23 | uncharacterized LOC285812 | 7.09372 | 6.77815 | 0.315577 | 1.24451 | 0.0046959 | 0.155506 |
| KIAA1147 | chr7q34 | KIAA1147 | 6.93732 | 6.67236 | 0.264962 | 1.2016 | 0.0036519 | 0.150674 |
| SPTBN1 | chr2p21 | spectrin, beta, non-erythrocytic 1 | 6.63296 | 6.35359 | 0.279367 | 1.21366 | 0.0006783 | 0.128628 |
| SSH1 | chr12q24.11 | slingshot protein phosphatase 1 | 6.12619 | 5.83621 | 0.289974 | 1.22262 | 0.0059012 | 0.160245 |
| TLE3 | chr15q22 | transducin-like enhancer of split 3 | 7.28048 | 6.95439 | 0.326097 | 1.25362 | 0.0002981 | 0.111326 |
| NKAP | chrXq24 | NFKB activating protein | 4.34548 | 4.02034 | 0.325143 | 1.25279 | 0.0136921 | 0.190461 |
| TNFRSF25 | chr1p36.2 | tumor necrosis factor receptor  superfamily, member 25 | 9.01862 | 8.71115 | 0.30747 | 1.23754 | 0.0145447 | 0.191711 |
| BCOR | chrXp11.4 | BCL6 corepressor | 5.78298 | 5.42693 | 0.356052 | 1.27992 | 0.0021379 | 0.147757 |
| CBX7 | chr22q13.1 | chromobox homolog 7 | 8.42318 | 8.05915 | 0.364036 | 1.28702 | 0.011209 | 0.182674 |
| TBCD | chr17q25.3 | tubulin folding cofactor D | 8.24937 | 7.81248 | 0.436888 | 1.35368 | 0.0035897 | 0.150674 |
| SQLE | chr8q24.1 | squalene epoxidase | 5.2273 | 4.93362 | 0.293682 | 1.22576 | 0.0034893 | 0.150538 |
| BCOR | chrXp11.4 | BCL6 corepressor | 7.94052 | 7.64549 | 0.295035 | 1.22691 | 0.0075664 | 0.168472 |
| ABHD15 | chr17q11.2 | abhydrolase domain containing 15 | 5.03261 | 4.71383 | 0.318783 | 1.24728 | 0.0056494 | 0.158791 |
| GPCPD1 | chr20p12.3 | glycerophosphocholine  phosphodiesterase 1 | 7.82898 | 7.55806 | 0.270922 | 1.20658 | 0.0156634 | 0.194435 |
| IL21R | chr16p11 | interleukin 21 receptor | 5.92289 | 5.59326 | 0.329629 | 1.25669 | 0.0034821 | 0.150538 |
| --- | --- | --- | 5.69182 | 5.36159 | 0.330231 | 1.25721 | 0.0082324 | 0.169323 |
| IL21R | chr16p11 | interleukin 21 receptor | 6.88872 | 6.55234 | 0.336378 | 1.26258 | 0.0059107 | 0.160245 |
| LDLRAD4 | chr18p11.21 | low density lipoprotein receptor class  A domain containing 4 | 6.98365 | 6.67068 | 0.312976 | 1.24227 | 0.0135724 | 0.190036 |
| PMEPA1 | chr20q13.31-  q13.33 | prostate transmembrane protein,  androgen induced 1 | 8.651 | 8.33169 | 0.319319 | 1.24774 | 0.0099658 | 0.175097 |
| CXCR3 | chrXq13 | chemokine (C-X-C motif) receptor 3 | 6.94107 | 6.63909 | 0.301984 | 1.23284 | 0.0129545 | 0.18773 |
| AUTS2 | chr7q11.22 | autism susceptibility candidate 2 | 9.57271 | 9.22786 | 0.344854 | 1.27002 | 0.0028821 | 0.148407 |
| PMEPA1 | chr20q13.31-  q13.33 | prostate transmembrane protein,  androgen induced 1 | 8.14474 | 7.7798 | 0.364945 | 1.28783 | 0.0033227 | 0.149592 |
| DLG5 | chr10q23 | discs, large homolog 5 (Drosophila) | 5.40245 | 5.01151 | 0.390944 | 1.31125 | 0.0033198 | 0.149592 |
| TMEM173 | chr5q31.2 | transmembrane protein 173 | 5.91423 | 5.6025 | 0.311733 | 1.2412 | 0.0161219 | 0.195587 |

| FAM219B | chr15q24.1 | family with sequence similarity 219,  member B | 6.13524 | 5.81733 | 0.317913 | 1.24653 | 0.0147112 | 0.192002 |
| --- | --- | --- | --- | --- | --- | --- | --- | --- |
| SLC7A5 | chr16q24.3 | solute carrier family 7 (amino acid  transporter light chain, L system), member 5 | 8.47579 | 8.11699 | 0.358802 | 1.28236 | 0.0068355 | 0.164807 |
| --- | --- | --- | 5.60564 | 5.16315 | 0.442482 | 1.35894 | 0.000555 | 0.124545 |
| NHSL2 | chrXq13.1 | NHS-like 2 | 5.60048 | 5.21831 | 0.38217 | 1.3033 | 0.011258 | 0.182674 |
| TM6SF1 | chr15q24-q26 | transmembrane 6 superfamily member  1 | 7.61923 | 7.25359 | 0.365639 | 1.28845 | 0.0040396 | 0.15147 |
| TGFBI | chr5q31 | transforming growth factor, beta-  induced, 68kDa | 7.84451 | 7.36996 | 0.474554 | 1.38949 | 0.002754 | 0.148407 |
| DACT1 | chr14q23.1 | dishevelled-binding antagonist of beta-  catenin 1 | 3.36854 | 3.09761 | 0.270929 | 1.20658 | 0.0171035 | 0.198257 |
| --- | --- | --- | 4.33487 | 4.06831 | 0.266561 | 1.20294 | 0.011023 | 0.181825 |
| KATNB1 | chr16q21 | katanin p80 (WD repeat containing)  subunit B 1 | 4.89619 | 4.61152 | 0.284672 | 1.21813 | 0.0084317 | 0.170307 |
| ZNF551 | chr19q13.43 | zinc finger protein 551 | 2.84875 | 2.54627 | 0.302481 | 1.23326 | 0.0147413 | 0.192002 |
| MRPS31 | chr13q14.11 | mitochondrial ribosomal protein S31 | 3.88354 | 3.59477 | 0.288773 | 1.2216 | 0.0052996 | 0.157883 |
| NCS1 | chr9q34 | neuronal calcium sensor 1 | 5.0489 | 4.71693 | 0.331977 | 1.25874 | 0.0121381 | 0.186382 |
| --- | --- | --- | 5.5774 | 5.26507 | 0.312331 | 1.24171 | 0.0034495 | 0.150538 |
| --- | --- | --- | 3.93 | 3.58628 | 0.343717 | 1.26902 | 0.0077717 | 0.168472 |
| ORAI2 | chr7q22.1 | ORAI calcium release-activated calcium  modulator 2 | 7.3973 | 7.12721 | 0.270092 | 1.20588 | 0.0080176 | 0.168575 |
| RGS12 | chr4p16.3 | regulator of G-protein signaling 12 | 5.73336 | 5.45551 | 0.277849 | 1.21239 | 0.0094574 | 0.173123 |
| ERGIC1 | chr5q35.1 | endoplasmic reticulum-golgi  intermediate compartment 1 | 6.91476 | 6.63229 | 0.282478 | 1.21628 | 0.0024736 | 0.148407 |
| PPIF | chr10q22.3 | peptidylprolyl isomerase F | 8.93246 | 8.62534 | 0.307116 | 1.23723 | 0.0068719 | 0.1654 |
| PTAFR | chr1p35-p34.3 | platelet-activating factor receptor | 7.20797 | 6.89156 | 0.316403 | 1.24522 | 0.015133 | 0.193396 |
| --- | --- | --- | 5.29073 | 4.89834 | 0.392394 | 1.31257 | 0.003212 | 0.149592 |
| MAP3K3 | chr17q23.3 | mitogen-activated protein kinase  kinase kinase 3 | 4.73097 | 4.38866 | 0.342301 | 1.26778 | 0.007169 | 0.166896 |
| --- | --- | --- | 4.77005 | 4.42587 | 0.344179 | 1.26943 | 0.0026187 | 0.148407 |
| CLN8 | chr8p23 | ceroid-lipofuscinosis, neuronal 8 | 6.82344 | 6.41986 | 0.403587 | 1.32279 | 0.0009858 | 0.129126 |
| TCAIM | chr3p21.31 | T cell activation inhibitor,  mitochondrial | 4.14585 | 3.80894 | 0.336913 | 1.26305 | 0.0064781 | 0.163699 |
| COG3 | chr13q14.13 | component of oligomeric golgi  complex 3 | 5.3227 | 5.04375 | 0.278946 | 1.21331 | 0.0096049 | 0.173173 |
| KCTD20 | chr6p21.31 | potassium channel tetramerization domain containing 20 | 5.55347 | 5.24529 | 0.30818 | 1.23814 | 0.0007183 | 0.128628 |
| MFSD14C | chr9q22.33 | major facilitator superfamily domain  containing 14C | 7.42366 | 7.15043 | 0.273231 | 1.20851 | 0.003538 | 0.150674 |
| CLASP2 | chr3p22.3 | cytoplasmic linker associated protein 2 | 6.21736 | 5.89359 | 0.323777 | 1.2516 | 0.0007645 | 0.128628 |
| --- | --- | --- | 5.67996 | 5.36551 | 0.314449 | 1.24354 | 0.0049966 | 0.157506 |
| ZBTB18 | chr1q44 | zinc finger and BTB domain containing  18 | 4.7128 | 4.36122 | 0.351586 | 1.27596 | 0.0055415 | 0.158791 |
| BROX | chr1q41 | BRO1 domain and CAAX motif  containing | 5.99262 | 5.59001 | 0.402607 | 1.32189 | 0.0014216 | 0.139801 |
| AKT3 | chr1q44 | v-akt murine thymoma viral oncogene  homolog 3 | 4.88929 | 4.59303 | 0.296265 | 1.22796 | 0.0060318 | 0.161186 |
| GRPEL2 | chr5q32 | GrpE-like 2, mitochondrial (E. coli) | 3.58026 | 3.13209 | 0.448176 | 1.36431 | 0.0003481 | 0.111326 |
| PHLDB2 | chr3q13.2 | pleckstrin homology-like domain,  family B, member 2 | 3.87434 | 3.61023 | 0.264105 | 1.20089 | 0.0001736 | 0.111326 |
| TRGV5 | chr7p14 | T cell receptor gamma variable 5 | 5.4114 | 5.14802 | 0.263378 | 1.20029 | 0.0062159 | 0.161814 |

| NBL1 | chr1p36.13 | neuroblastoma 1, DAN family BMP  antagonist | 5.91622 | 5.62121 | 0.295012 | 1.22689 | 0.0001219 | 0.111326 |
| --- | --- | --- | --- | --- | --- | --- | --- | --- |
| C12orf66 | chr12q14.2 | chromosome 12 open reading frame  66 | 3.40966 | 3.14569 | 0.26397 | 1.20078 | 0.0085034 | 0.170624 |
| LSM14B | chr20q13.33 | LSM family member 14B | 3.50962 | 3.21085 | 0.298775 | 1.2301 | 0.0027131 | 0.148407 |
| HOXB-AS1 | --- | HOXB cluster antisense RNA 1 | 4.2713 | 3.966 | 0.305298 | 1.23567 | 0.0109291 | 0.181825 |
| CLIC5 | chr6p12.3 | chloride intracellular channel 5 | 5.81314 | 5.48888 | 0.324259 | 1.25202 | 0.0109337 | 0.181825 |
| SLC39A13 | chr11p11.2 | solute carrier family 39 (zinc  transporter), member 13 | 5.79755 | 5.52241 | 0.275144 | 1.21012 | 0.0159367 | 0.195343 |
| EML2 | chr19q13.32 | echinoderm microtubule associated  protein like 2 | 6.0704 | 5.77759 | 0.292803 | 1.22502 | 0.0025705 | 0.148407 |
| SLC1A4 | chr2p15-p13 | solute carrier family 1  (glutamate/neutral amino acid transporter), member 4 | 5.59925 | 5.28717 | 0.312079 | 1.2415 | 0.0096162 | 0.173243 |
| ZNF777 | chr7q36.1 | zinc finger protein 777 | 3.72722 | 3.35602 | 0.371192 | 1.29342 | 0.0032678 | 0.149592 |
| NCR1 | chr19q13.42 | natural cytotoxicity triggering receptor  1 | 4.22221 | 3.8722 | 0.350019 | 1.27458 | 0.0061614 | 0.161641 |
| TOP3A | chr17p11.2 | topoisomerase (DNA) III alpha | 3.33013 | 2.94448 | 0.385651 | 1.30645 | 0.0032739 | 0.149592 |
| STAT5B | chr17q11.2 | signal transducer and activator of  transcription 5B | 5.97199 | 5.70198 | 0.270008 | 1.20581 | 0.0107668 | 0.18109 |
| METTL6 | chr3p25.1 | methyltransferase like 6 | 2.77215 | 2.48484 | 0.287307 | 1.22036 | 0.0126804 | 0.187417 |
| KCNAB2 | chr1p36.3 | potassium channel, voltage gated subfamily A regulatory beta subunit 2 | 5.01129 | 4.72987 | 0.28142 | 1.21539 | 0.00647 | 0.163699 |
| LEKR1 | chr3q25.31 | leucine, glutamate and lysine rich 1 | 3.94592 | 3.63541 | 0.310507 | 1.24014 | 0.0013401 | 0.137103 |
| --- | --- | --- | 3.28391 | 2.97289 | 0.311022 | 1.24059 | 0.0072844 | 0.167406 |
| CASK | chrXp11.4 | calcium/calmodulin-dependent serine protein kinase (MAGUK family) | 4.94487 | 4.67555 | 0.269322 | 1.20524 | 0.0009277 | 0.128628 |
| SPON1 | chr11p15.2 | spondin 1, extracellular matrix protein | 4.33465 | 4.06162 | 0.27303 | 1.20834 | 0.0030314 | 0.148407 |
| RPAP2 | chr1p22.1 | RNA polymerase II associated protein 2 | 4.91261 | 4.62808 | 0.284528 | 1.21801 | 0.0013667 | 0.137515 |
| ZC3H18 | chr16q24.2 | zinc finger CCCH-type containing 18 | 4.39702 | 4.13236 | 0.26466 | 1.20135 | 0.0008043 | 0.128628 |
| MECR | chr1p35.3 | mitochondrial trans-2-enoyl-CoA  reductase | 4.14097 | 3.84806 | 0.292906 | 1.22511 | 0.0013955 | 0.138618 |
| TTC17 | chr11p11.2 | tetratricopeptide repeat domain 17 | 2.9899 | 2.69165 | 0.298252 | 1.22965 | 0.0051152 | 0.157506 |
| IPP | chr1p34-p32 | intracisternal A particle-promoted  polypeptide | 4.10761 | 3.80342 | 0.304192 | 1.23473 | 0.0040594 | 0.151685 |
| RPRD1A | chr18q12.2 | regulation of nuclear pre-mRNA  domain containing 1A | 5.6101 | 5.34394 | 0.266158 | 1.2026 | 0.004899 | 0.157154 |
| SOS2 | chr14q21 | SOS Ras/Rho guanine nucleotide  exchange factor 2 | 5.77084 | 5.44037 | 0.33047 | 1.25742 | 0.0030733 | 0.148407 |
| KDELR1 | chr19q13.3 | KDEL (Lys-Asp-Glu-Leu) endoplasmic reticulum protein retention receptor 1 | 6.18169 | 5.8616 | 0.320095 | 1.24841 | 0.0062637 | 0.162624 |
| LOC101927  055 /// TTN- AS1 | --- | uncharacterized LOC101927055 /// TTN antisense RNA 1 | 4.56268 | 4.28179 | 0.280891 | 1.21494 | 0.0171061 | 0.198257 |
| --- | --- | --- | 2.60901 | 2.29365 | 0.315357 | 1.24432 | 0.0128962 | 0.187472 |
| CHP1 | chr15q13.3 | calcineurin-like EF-hand protein 1 | 5.15365 | 4.75091 | 0.402739 | 1.32202 | 0.0032602 | 0.149592 |
| SPATC1L | chr21q22.3 | spermatogenesis and centriole  associated 1-like | 4.79371 | 4.44032 | 0.353398 | 1.27757 | 0.014215 | 0.191429 |
| KLHL22 | chr22q11.21 | kelch-like family member 22 | 4.91336 | 4.56217 | 0.351193 | 1.27562 | 0.0017011 | 0.145076 |

| BTN3A1 | chr6p22.1 | butyrophilin, subfamily 3, member A1 | 7.91623 | 7.53552 | 0.380711 | 1.30198 | 0.0066256 | 0.16393 |
| --- | --- | --- | --- | --- | --- | --- | --- | --- |
| RAD51L3-  RFFL /// RFFL | chr17q /// chr17q12 | RAD51L3-RFFL readthrough /// ring finger and FYVE-like domain containing E3 ubiquitin protein ligase | 5.26341 | 4.87974 | 0.383661 | 1.30465 | 0.0089942 | 0.172413 |
| TCAF2 | chr7q35 | TRPM8 channel-associated factor 2 | 5.91196 | 5.53989 | 0.372078 | 1.29422 | 0.0003183 | 0.111326 |
| SLC7A6 | chr16q22.1 | solute carrier family 7 (amino acid  transporter light chain, y+L system), member 6 | 7.23779 | 6.97426 | 0.263526 | 1.20041 | 0.0072131 | 0.167221 |
| BTN3A2 | chr6p22.1 | butyrophilin, subfamily 3, member A2 | 9.32774 | 9.05445 | 0.273288 | 1.20856 | 0.0127943 | 0.187426 |
| PARP6 | chr15q23 | poly(ADP-ribose) polymerase family  member 6 | 7.2666 | 6.98339 | 0.283209 | 1.2169 | 0.0126466 | 0.187372 |
| MBP | chr18q23 | myelin basic protein | 8.48897 | 8.20733 | 0.281634 | 1.21557 | 0.0026037 | 0.148407 |
| PAFAH2 | chr1p36 | platelet-activating factor  acetylhydrolase 2 | 6.22174 | 5.92891 | 0.29283 | 1.22504 | 0.0026579 | 0.148407 |
| AKR7A2 | chr1p36.13 | aldo-keto reductase family 7, member  A2 | 7.07538 | 6.798 | 0.277385 | 1.212 | 0.0070731 | 0.166748 |
| PMS2P3 | chr7q11.23 | PMS1 homolog 2, mismatch repair  system component pseudogene 3 | 7.35656 | 7.0487 | 0.307863 | 1.23787 | 0.0034829 | 0.150538 |
| PIGC | chr1q23-q25 | phosphatidylinositol glycan anchor  biosynthesis class C | 6.70372 | 6.37417 | 0.329555 | 1.25663 | 0.0058256 | 0.160158 |
| EML4 | chr2p21 | echinoderm microtubule associated  protein like 4 | 8.37903 | 8.0288 | 0.350226 | 1.27476 | 0.00204 | 0.147757 |
| LSP1 | chr11p15.5 | lymphocyte-specific protein 1 | 7.88508 | 7.53161 | 0.353462 | 1.27762 | 0.0026639 | 0.148407 |
| HPS3 | chr3q24 | Hermansky-Pudlak syndrome 3 | 5.59889 | 5.32259 | 0.276295 | 1.21108 | 0.0121181 | 0.186382 |
| --- | --- | --- | 5.35164 | 5.05018 | 0.30146 | 1.23239 | 0.0018843 | 0.14618 |
| EPB41 | chr1p33-p32 | erythrocyte membrane protein band  4.1 | 6.14267 | 5.77841 | 0.36426 | 1.28722 | 0.0007487 | 0.128628 |
| PIGX | chr3q29 | phosphatidylinositol glycan anchor  biosynthesis class X | 7.57811 | 7.19426 | 0.383843 | 1.30481 | 0.0025865 | 0.148407 |
| RBM48 | chr7q21.2 | RNA binding motif protein 48 | 6.33261 | 5.93721 | 0.395397 | 1.3153 | 0.0013486 | 0.137103 |
| PLAU | chr10q22.2 | plasminogen activator, urokinase | 3.77766 | 3.39284 | 0.384818 | 1.3057 | 0.0020653 | 0.147757 |
| KIAA0430 | chr16p13.11 | KIAA0430 | 4.23946 | 3.82477 | 0.414691 | 1.33301 | 0.000881 | 0.128628 |
| TFRC | chr3q29 | transferrin receptor | 4.39311 | 4.04335 | 0.349756 | 1.27435 | 0.0144869 | 0.191711 |
| SS18 | chr18q11.2 | synovial sarcoma translocation,  chromosome 18 | 5.94639 | 5.60463 | 0.34176 | 1.2673 | 0.0092179 | 0.172989 |
| CBFB | chr16q22.1 | core-binding factor, beta subunit | 7.67475 | 7.2164 | 0.458354 | 1.37397 | 0.0054798 | 0.158791 |
| HSPA4 | chr5q31.1 | heat shock 70kDa protein 4 | 7.13349 | 6.86967 | 0.263821 | 1.20065 | 0.0124337 | 0.187004 |
| BICD2 | chr9q22.31 | bicaudal D homolog 2 (Drosophila) | 6.78183 | 6.48767 | 0.294158 | 1.22617 | 0.0020915 | 0.147757 |
| DAGLB | chr7p22.1 | diacylglycerol lipase, beta | 5.87898 | 5.57388 | 0.305104 | 1.23551 | 0.0113383 | 0.183076 |
| GNB5 | chr15q21.2 | guanine nucleotide binding protein (G  protein), beta 5 | 6.33641 | 6.0545 | 0.281909 | 1.2158 | 0.0037621 | 0.150735 |
| SEC22C | chr3p22.1 | SEC22 homolog C, vesicle trafficking  protein | 5.47781 | 5.1595 | 0.318306 | 1.24687 | 0.0012264 | 0.136585 |
| CHD1L | chr1q12 | chromodomain helicase DNA binding  protein 1-like | 4.78677 | 4.45973 | 0.32704 | 1.25444 | 0.002168 | 0.147979 |
| G3BP1 | chr5q33.1 | GTPase activating protein (SH3  domain) binding protein 1 | 5.85107 | 5.52833 | 0.322738 | 1.2507 | 0.0125902 | 0.187104 |
| ARL4C | chr2q37.1 | ADP-ribosylation factor like GTPase 4C | 9.11661 | 8.79502 | 0.321586 | 1.2497 | 0.0089083 | 0.172413 |
| DOCK8 | chr9p24.3 | dedicator of cytokinesis 8 | 7.80624 | 7.46986 | 0.336374 | 1.26258 | 0.0061538 | 0.161641 |
| USP28 | chr11q23 | ubiquitin specific peptidase 28 | 6.08245 | 5.77007 | 0.312382 | 1.24176 | 0.0128177 | 0.187472 |

| TBRG1 | chr11q24.2 | transforming growth factor beta  regulator 1 | 7.45885 | 7.15774 | 0.30111 | 1.23209 | 0.0173526 | 0.198289 |
| --- | --- | --- | --- | --- | --- | --- | --- | --- |
| UBE2G2 | chr21q22.3 | ubiquitin conjugating enzyme E2G 2 | 6.45833 | 6.11057 | 0.347763 | 1.27259 | 0.0160623 | 0.195587 |
| RAB5A | chr3p24.3 | RAB5A, member RAS oncogene family | 7.73251 | 7.4309 | 0.301608 | 1.23252 | 0.0139374 | 0.190686 |
| YWHAZ | chr8q23.1 | tyrosine 3-  monooxygenase/tryptophan 5- monooxygenase activation protein,  zeta | 10.8687 | 10.5543 | 0.314369 | 1.24347 | 0.0070834 | 0.166748 |
| PICALM | chr11q14 | phosphatidylinositol binding clathrin  assembly protein | 7.44738 | 7.0961 | 0.351279 | 1.27569 | 0.0123719 | 0.187004 |
| SRPR | chr11q24.2 | signal recognition particle receptor  (docking protein) | 6.74705 | 6.3779 | 0.369144 | 1.29159 | 0.0097883 | 0.174094 |
| GSR | chr8p21.1 | glutathione reductase | 6.79936 | 6.51637 | 0.282992 | 1.21672 | 0.0087468 | 0.17126 |
| MBNL2 | chr13q32.1 | muscleblind-like splicing regulator 2 | 5.13925 | 4.84345 | 0.295806 | 1.22757 | 0.0148892 | 0.19252 |
| MDM4 | chr1q32 | MDM4, p53 regulator | 5.64095 | 5.375 | 0.265952 | 1.20243 | 0.0164646 | 0.196619 |
| RARS2 | chr6q16.1 | arginyl-tRNA synthetase 2,  mitochondrial | 4.39264 | 4.09814 | 0.294505 | 1.22646 | 0.0045744 | 0.155277 |
| CBFA2T2 | chr20q11 | core-binding factor, runt domain,  alpha subunit 2; translocated to, 2 | 5.13903 | 4.75865 | 0.380381 | 1.30169 | 0.0003412 | 0.111326 |
| RBBP9 | chr20p11.2 | retinoblastoma binding protein 9 | 3.36822 | 3.06063 | 0.307588 | 1.23764 | 0.0180864 | 0.198866 |
| FANCF | chr11p15 | Fanconi anemia complementation  group F | 5.60061 | 5.31094 | 0.289674 | 1.22236 | 0.0029356 | 0.148407 |
| RHOF | chr12q24.31 | ras homolog family member F (in  filopodia) | 6.25501 | 5.95744 | 0.297573 | 1.22908 | 0.0015856 | 0.145076 |
| ALAD | chr9q33.1 | aminolevulinate dehydratase | 4.39072 | 4.06167 | 0.329047 | 1.25618 | 0.002062 | 0.147757 |
| AMPD3 | chr11p15 | adenosine monophosphate deaminase  3 | 4.31173 | 4.01964 | 0.292094 | 1.22442 | 0.0086128 | 0.170682 |
| R3HCC1L | chr10q24.2 | R3H domain and coiled-coil containing  1-like | 5.66812 | 5.29999 | 0.368135 | 1.29068 | 0.0110097 | 0.181825 |
| PTBP3 | chr9q32 | polypyrimidine tract binding protein 3 | 5.77168 | 5.46538 | 0.306304 | 1.23654 | 0.0179205 | 0.198662 |
| GLE1 | chr9q34.11 | GLE1 RNA export mediator | 6.27986 | 5.963 | 0.316859 | 1.24562 | 0.0123087 | 0.186978 |
| PDLIM5 | chr4q22 | PDZ and LIM domain 5 | 5.33121 | 4.91189 | 0.419323 | 1.3373 | 0.0035625 | 0.150674 |
| CHM | chrXq21.2 | choroideremia (Rab escort protein 1) | 4.4731 | 4.14287 | 0.330231 | 1.25721 | 0.0180386 | 0.198822 |
| CLASP1 | chr2q14.2-q14.3 | cytoplasmic linker associated protein 1 | 4.65831 | 4.29976 | 0.358553 | 1.28214 | 0.0164972 | 0.196619 |
| SOCS1 | chr16p13.13 | suppressor of cytokine signaling 1 | 5.15098 | 4.67831 | 0.472662 | 1.38767 | 0.0003809 | 0.111326 |
| --- | --- | --- | 5.16411 | 4.86776 | 0.296345 | 1.22803 | 0.0122226 | 0.186495 |
| METTL15 | chr11p14.1 | methyltransferase like 15 | 3.25031 | 2.91538 | 0.334937 | 1.26132 | 0.0113925 | 0.183408 |
| PIGM | chr1q23.2 | phosphatidylinositol glycan anchor  biosynthesis class M | 6.19158 | 5.90481 | 0.286777 | 1.21991 | 0.0149656 | 0.192697 |
| FBXO32 | chr8q24.13 | F-box protein 32 | 4.41297 | 4.13559 | 0.27738 | 1.21199 | 0.0071014 | 0.166748 |
| --- | --- | --- | 2.45739 | 2.16993 | 0.287465 | 1.22049 | 0.0164572 | 0.196619 |
| LARP4B | chr10p15.3 | La ribonucleoprotein domain family,  member 4B | 4.53449 | 4.17537 | 0.35912 | 1.28264 | 0.006909 | 0.16574 |
| FNTB | chr14q23.3 | farnesyltransferase, CAAX box, beta | 4.51964 | 4.14511 | 0.374526 | 1.29641 | 0.0022203 | 0.148212 |
| FAM105A | chr5p15.2 | family with sequence similarity 105,  member A | 4.60867 | 4.31345 | 0.295223 | 1.22707 | 0.0056607 | 0.158791 |
| NKD1 | chr16q12.1 | naked cuticle homolog 1 (Drosophila) | 6.05625 | 5.67826 | 0.377989 | 1.29953 | 0.0038592 | 0.150786 |
| --- | --- | --- | 6.73619 | 6.44397 | 0.292223 | 1.22453 | 0.0023378 | 0.148407 |
| --- | --- | --- | 5.37374 | 5.07754 | 0.296198 | 1.2279 | 0.0044495 | 0.153634 |

| WDR60 | chr7q36.3 | WD repeat domain 60 | 5.24835 | 4.9836 | 0.264748 | 1.20143 | 0.0009825 | 0.129126 |
| --- | --- | --- | --- | --- | --- | --- | --- | --- |
| SLC25A51 | chr9p13.3-p12 | solute carrier family 25, member 51 | 4.81066 | 4.54281 | 0.267855 | 1.20402 | 0.0029425 | 0.148407 |
| ZNF416 | chr19q13.4 | zinc finger protein 416 | 3.59899 | 3.32061 | 0.27838 | 1.21283 | 0.0023876 | 0.148407 |
| --- | --- | --- | 5.84833 | 5.57547 | 0.272861 | 1.2082 | 0.0029558 | 0.148407 |
| EXO5 | chr1p34.2 | exonuclease 5 | 2.86067 | 2.59164 | 0.269031 | 1.205 | 0.0005942 | 0.128154 |
| --- | --- | --- | 4.05596 | 3.73374 | 0.322229 | 1.25026 | 0.0001043 | 0.111326 |
| --- | --- | --- | 3.42973 | 3.15706 | 0.272666 | 1.20804 | 0.0009525 | 0.128628 |
| FOXO3 ///  FOXO3B | chr17p11.2 ///  chr6q21 | forkhead box O3 /// forkhead box O3B  pseudogene | 6.63365 | 6.34567 | 0.28798 | 1.22093 | 0.001181 | 0.134826 |
| COX15 | chr10q24 | cytochrome c oxidase assembly  homolog 15 (yeast) | 5.2335 | 4.87306 | 0.360443 | 1.28382 | 8.466E-05 | 0.111326 |
| QTRT1 | chr19p13.3 | queuine tRNA-ribosyltransferase 1 | 5.8996 | 5.63637 | 0.263228 | 1.20016 | 0.0103441 | 0.178293 |
| --- | --- | --- | 5.32164 | 5.05437 | 0.26727 | 1.20353 | 0.0058146 | 0.160158 |
| LOC101930  105 /// LOC389906  ///  LOC441528 | chrXp22.33 | zinc finger protein 839-like /// zinc finger protein 839 pseudogene /// zinc  finger protein 839-like | 4.29085 | 4.00748 | 0.283376 | 1.21704 | 0.0095916 | 0.173173 |
| ARSG | chr17q24.2 | arylsulfatase G | 3.92441 | 3.59456 | 0.329854 | 1.25689 | 0.0064624 | 0.163699 |
| --- | --- | --- | 5.65119 | 5.35086 | 0.300329 | 1.23143 | 0.0080997 | 0.169089 |
| LRCH3 | chr3q29 | leucine-rich repeats and calponin homology (CH) domain containing 3 | 6.23397 | 5.89855 | 0.335428 | 1.26175 | 0.0069854 | 0.165923 |
| RALGAPA1 | chr14q13.2 | Ral GTPase activating protein, alpha  subunit 1 (catalytic) | 6.56163 | 6.2599 | 0.301731 | 1.23262 | 0.0146464 | 0.191814 |
| --- | --- | --- | 6.77329 | 6.41619 | 0.357095 | 1.28084 | 0.0016744 | 0.145076 |
| --- | --- | --- | 4.96064 | 4.63316 | 0.327476 | 1.25482 | 0.0106615 | 0.180552 |
| --- | --- | --- | 5.62364 | 5.2425 | 0.381136 | 1.30237 | 0.0021644 | 0.147979 |
| NUP50 | chr22q13.31 | nucleoporin 50kDa | 3.21183 | 2.92758 | 0.284249 | 1.21778 | 0.0005162 | 0.122884 |
| SDCCAG8 | chr1q43 | serologically defined colon cancer  antigen 8 | 5.61322 | 5.32346 | 0.289764 | 1.22244 | 0.007384 | 0.167706 |
| RAB6A | chr11q13.3 | RAB6A, member RAS oncogene family | 4.23728 | 3.92588 | 0.311398 | 1.24091 | 0.0005685 | 0.125351 |
| B4GALT1 | chr9p13 | UDP-Gal:betaGlcNAc beta 1,4-  galactosyltransferase, polypeptide 1 | 4.38447 | 4.12118 | 0.263291 | 1.20021 | 0.0132859 | 0.189533 |
| --- | --- | --- | 3.13606 | 2.8713 | 0.264765 | 1.20144 | 0.016071 | 0.195587 |
| RALGAPA1 | chr14q13.2 | Ral GTPase activating protein, alpha  subunit 1 (catalytic) | 5.15202 | 4.86837 | 0.283655 | 1.21728 | 0.016024 | 0.195587 |
| AAK1 | chr2p14 | AP2 associated kinase 1 | 3.92718 | 3.65902 | 0.268157 | 1.20427 | 0.0040379 | 0.15147 |
| COX15 | chr10q24 | cytochrome c oxidase assembly  homolog 15 (yeast) | 5.89075 | 5.57234 | 0.318411 | 1.24696 | 4.967E-05 | 0.107736 |
| CALM1 | chr14q32.11 | calmodulin 1 (phosphorylase kinase,  delta) | 6.1678 | 5.9032 | 0.264602 | 1.2013 | 0.0139325 | 0.190686 |
| BRF1 | chr14q | BRF1, RNA polymerase III transcription initiation factor 90 kDa subunit | 4.22193 | 3.89353 | 0.328406 | 1.25563 | 0.0026982 | 0.148407 |
| SIGMAR1 | chr9p13.3 | sigma non-opioid intracellular receptor  1 | 5.48457 | 5.20084 | 0.283724 | 1.21733 | 0.0023508 | 0.148407 |
| FLT3LG | chr19q13.3 | fms-related tyrosine kinase 3 ligand | 5.80964 | 5.49997 | 0.309672 | 1.23943 | 0.0096255 | 0.173245 |
| ATP2A3 | chr17p13.3 | ATPase, Ca++ transporting, ubiquitous | 4.35732 | 4.02692 | 0.3304 | 1.25736 | 0.0027581 | 0.148407 |
| FMNL3 | chr12q13.12 | formin like 3 | 5.52033 | 5.22929 | 0.291041 | 1.22352 | 0.0084788 | 0.170624 |
| --- | --- | --- | 4.94201 | 4.6767 | 0.26531 | 1.20189 | 0.002151 | 0.147979 |

| PATZ1 | chr22q12.2 | POZ (BTB) and AT hook containing zinc  finger 1 | 5.13527 | 4.83862 | 0.296648 | 1.22829 | 0.0038519 | 0.150786 |
| --- | --- | --- | --- | --- | --- | --- | --- | --- |
| --- | --- | --- | 4.91938 | 4.58754 | 0.331838 | 1.25862 | 0.0018962 | 0.14618 |
| ALDH5A1 | chr6p22 | aldehyde dehydrogenase 5 family,  member A1 | 3.48403 | 3.21408 | 0.269952 | 1.20577 | 0.0041741 | 0.151901 |
| SEPT2 | chr2q37 | septin 2 | 5.98022 | 5.65973 | 0.320494 | 1.24876 | 0.0019083 | 0.14618 |
| --- | --- | --- | 6.61759 | 6.35214 | 0.26546 | 1.20202 | 0.0061505 | 0.161641 |
| --- | --- | --- | 6.41884 | 6.13386 | 0.284974 | 1.21839 | 0.0006203 | 0.128628 |
| ATF6B | chr6p21.3 | activating transcription factor 6 beta | 5.64471 | 5.33022 | 0.314488 | 1.24357 | 0.0019114 | 0.14618 |
| --- | --- | --- | 5.96453 | 5.63565 | 0.328877 | 1.25604 | 0.0025114 | 0.148407 |
| --- | --- | --- | 6.34439 | 5.99572 | 0.348672 | 1.27339 | 0.0003781 | 0.111326 |
| NGLY1 | chr3p24.2 | N-glycanase 1 | 6.62899 | 6.22232 | 0.406673 | 1.32563 | 0.0013328 | 0.137103 |
| CDC14A | chr1p21 | cell division cycle 14A | 5.07305 | 4.78848 | 0.284569 | 1.21805 | 0.0148453 | 0.192417 |
| --- | --- | --- | 5.76753 | 5.4783 | 0.289227 | 1.22199 | 0.0125545 | 0.187104 |
| ZNF818P | chr19q13.42 | zinc finger protein 818, pseudogene | 3.27957 | 2.97683 | 0.302739 | 1.23348 | 0.0057073 | 0.158791 |
| GTF2IRD2  /// GTF2IRD2B | chr7q11.23 | GTF2I repeat domain containing 2 /// GTF2I repeat domain containing 2B | 4.70867 | 4.41635 | 0.292321 | 1.22461 | 0.0024152 | 0.148407 |
| KLHL42 | chr12p11.22 | kelch-like family member 42 | 5.01605 | 4.70146 | 0.314586 | 1.24365 | 0.0055766 | 0.158791 |
| --- | --- | --- | 4.65235 | 4.33341 | 0.318937 | 1.24741 | 0.000612 | 0.128628 |
| ERN1 | chr17q24.2 | endoplasmic reticulum to nucleus  signaling 1 | 7.33223 | 6.99278 | 0.339452 | 1.26528 | 0.0018711 | 0.14618 |
| --- | --- | --- | 4.93176 | 4.50538 | 0.426384 | 1.34386 | 0.0001824 | 0.111326 |
| DLST | chr14q24.3 | dihydrolipoamide S-succinyltransferase (E2 component of 2-oxo-glutarate complex) | 7.97465 | 7.65902 | 0.315624 | 1.24455 | 0.0046272 | 0.155277 |
| ERGIC1 | chr5q35.1 | endoplasmic reticulum-golgi  intermediate compartment 1 | 6.48598 | 6.18818 | 0.297795 | 1.22926 | 0.0080738 | 0.169033 |
| PLXNC1 | chr12q23.3 | plexin C1 | 3.99454 | 3.67525 | 0.319289 | 1.24772 | 0.0028527 | 0.148407 |
| SKIL | chr3q26 | SKI-like proto-oncogene | 5.50819 | 5.22744 | 0.280752 | 1.21483 | 0.0071623 | 0.166896 |
| CDKN1C | chr11p15.5 | cyclin-dependent kinase inhibitor 1C  (p57, Kip2) | 5.48336 | 5.2158 | 0.26756 | 1.20377 | 0.0016266 | 0.145076 |
| --- | --- | --- | 4.86084 | 4.58603 | 0.274815 | 1.20984 | 0.0037279 | 0.150735 |
| SRSF1 | chr17q22 | serine/arginine-rich splicing factor 1 | 4.16418 | 3.88095 | 0.283232 | 1.21692 | 0.0018783 | 0.14618 |
| --- | --- | --- | 4.13051 | 3.80267 | 0.327837 | 1.25513 | 0.0017904 | 0.145076 |
| NPHP3 | chr3q22.1 | nephronophthisis 3 (adolescent) | 4.19182 | 3.84218 | 0.349644 | 1.27425 | 0.0053061 | 0.157883 |
| RNASEL | chr1q25 | ribonuclease L (2',5'-oligoisoadenylate synthetase-dependent) | 5.70255 | 5.4313 | 0.27125 | 1.20685 | 0.0092225 | 0.172989 |
| --- | --- | --- | 3.83596 | 3.55803 | 0.277931 | 1.21246 | 0.0146171 | 0.191814 |
| XRRA1 | chr11q13.4 | X-ray radiation resistance associated 1 | 3.75133 | 3.48365 | 0.267686 | 1.20388 | 0.0075715 | 0.168472 |
| TAF8 | chr6p21.1 | TATA box binding protein associated  factor 8 | 5.60958 | 5.34146 | 0.268121 | 1.20424 | 0.0180046 | 0.198662 |
| --- | --- | --- | 4.7344 | 4.43332 | 0.301081 | 1.23207 | 0.0075486 | 0.168472 |
| --- | --- | --- | 6.47623 | 6.1892 | 0.287032 | 1.22013 | 0.011401 | 0.183421 |
| --- | --- | --- | 4.80338 | 4.49976 | 0.303621 | 1.23424 | 0.0148715 | 0.19252 |
| PRMT5-AS1 | chr14q11.2 | PRMT5 antisense RNA 1 | 3.07371 | 2.8029 | 0.270813 | 1.20649 | 0.0043756 | 0.152986 |
| LOC100289  058 | chr13q14.11 | uncharacterized LOC100289058 | 4.79175 | 4.46723 | 0.324517 | 1.25224 | 0.0069612 | 0.165836 |

| SYNRG | chr17q12 | synergin, gamma | 5.23196 | 4.94038 | 0.291576 | 1.22398 | 0.0079996 | 0.168566 |
| --- | --- | --- | --- | --- | --- | --- | --- | --- |
| AGO4 | chr1p34 | argonaute RISC catalytic component 4 | 3.10756 | 2.78321 | 0.324352 | 1.2521 | 0.0021432 | 0.147831 |
| --- | --- | --- | 3.74673 | 3.41499 | 0.331734 | 1.25853 | 0.0016388 | 0.145076 |
| --- | --- | --- | 4.3075 | 4.00248 | 0.305017 | 1.23543 | 0.0109399 | 0.181825 |
| COPB1 | chr11p15.2 | coatomer protein complex subunit  beta 1 | 4.80074 | 4.45447 | 0.346268 | 1.27127 | 0.0137215 | 0.190636 |
| RBM26 | chr13q31.1 | RNA binding motif protein 26 | 4.38854 | 4.00818 | 0.38036 | 1.30167 | 0.0016396 | 0.145076 |
| SMCR8 | chr17p11.2 | Smith-Magenis syndrome chromosome  region, candidate 8 | 4.62494 | 4.26412 | 0.36082 | 1.28416 | 0.0061651 | 0.161641 |
| HIST1H3I | chr6p22.1 | histone cluster 1, H3i | 5.00227 | 4.51548 | 0.486788 | 1.40132 | 0.0007887 | 0.128628 |
| JAM3 | chr11q25 | junctional adhesion molecule 3 | 4.26454 | 3.99955 | 0.264985 | 1.20162 | 0.0068182 | 0.164807 |
| MORN3 | chr12q24.31 | MORN repeat containing 3 | 4.94708 | 4.67248 | 0.2746 | 1.20966 | 0.0024222 | 0.148407 |
| UBE2W | chr8q21.11 | ubiquitin-conjugating enzyme E2W  (putative) | 3.29417 | 3.00375 | 0.290421 | 1.223 | 0.0125222 | 0.187104 |
| VPS53 | chr17p13.3 | vacuolar protein sorting 53 homolog  (S. cerevisiae) | 3.48034 | 3.18581 | 0.294524 | 1.22648 | 0.0036204 | 0.150674 |
| --- | --- | --- | 4.3887 | 4.08674 | 0.301963 | 1.23282 | 0.0007956 | 0.128628 |
| SELM | chr22q12.2 | selenoprotein M | 6.02099 | 5.73757 | 0.283413 | 1.21707 | 0.0127845 | 0.187417 |
| GPR55 | chr2q37 | G protein-coupled receptor 55 | 4.35351 | 4.06419 | 0.289317 | 1.22206 | 0.0088214 | 0.172107 |
| APBB1IP | chr10p12.1 | amyloid beta (A4) precursor protein-  binding, family B, member 1 interacting protein | 6.42793 | 6.14536 | 0.282574 | 1.21636 | 0.0085495 | 0.170624 |
| PMS2P1 | chr7q22.1 | PMS1 homolog 2, mismatch repair  system component pseudogene 1 | 7.15562 | 6.8884 | 0.267219 | 1.20349 | 0.0115069 | 0.18411 |
| YPEL1 | chr22q11.2 | yippee like 1 | 6.17192 | 5.8748 | 0.297118 | 1.22869 | 0.0011672 | 0.134826 |
| LIAS | chr4p14 | lipoic acid synthetase | 6.00581 | 5.72362 | 0.282187 | 1.21604 | 0.0036461 | 0.150674 |
| ZNF276 | chr16q24.3 | zinc finger protein 276 | 6.26673 | 5.98637 | 0.280359 | 1.2145 | 0.0007144 | 0.128628 |
| PMS2P3 | chr7q11.23 | PMS1 homolog 2, mismatch repair  system component pseudogene 3 | 8.3756 | 8.06781 | 0.307788 | 1.23781 | 0.002597 | 0.148407 |
| PVRIG | chr7q22.1 | poliovirus receptor related immunoglobulin domain containing | 7.80157 | 7.53117 | 0.270401 | 1.20614 | 0.001882 | 0.14618 |
| DHRS7 | chr14q23.1 | dehydrogenase/reductase (SDR family)  member 7 | 8.60144 | 8.31763 | 0.283812 | 1.21741 | 0.0020462 | 0.147757 |
| LINC00667 | chr18p11.31 | long intergenic non-protein coding  RNA 667 | 6.40785 | 6.0915 | 0.316352 | 1.24518 | 0.0004442 | 0.113339 |
| SH2D1B | chr1q23.3 | SH2 domain containing 1B | 8.85129 | 8.57998 | 0.271308 | 1.2069 | 0.012902 | 0.187472 |
| HIVEP3 | chr1p34 | human immunodeficiency virus type I  enhancer binding protein 3 | 6.43177 | 6.15445 | 0.277315 | 1.21194 | 0.0072417 | 0.167288 |
| VASH1 | chr14q24.3 | vasohibin 1 | 5.92917 | 5.54061 | 0.388568 | 1.30909 | 0.0012597 | 0.136585 |
| SEPT9 | chr17q25 | septin 9 | 4.71699 | 4.38702 | 0.329971 | 1.25699 | 0.0157921 | 0.194682 |
| EPRS | chr1q41 | glutamyl-prolyl-tRNA synthetase | 7.32646 | 6.95793 | 0.368536 | 1.29104 | 0.0071893 | 0.167056 |
| MEX3C | chr18q21.2 | mex-3 RNA binding family member C | 5.99424 | 5.62528 | 0.368958 | 1.29142 | 0.0051083 | 0.157506 |
| MSANTD4 | chr11q22 | Myb/SANT-like DNA-binding domain containing 4 with coiled-coils | 5.37407 | 5.05899 | 0.315074 | 1.24408 | 0.0050058 | 0.157506 |
| ICE2 | chr15q22.2 | interactor of little elongation complex  ELL subunit 2 | 5.55271 | 5.07165 | 0.481059 | 1.39577 | 0.0003641 | 0.111326 |
| NFATC2 | chr20q13.2 | nuclear factor of activated T-cells, cytoplasmic, calcineurin-dependent 2 | 5.12922 | 4.75144 | 0.377776 | 1.29934 | 0.0030576 | 0.148407 |
| HERC1 | chr15q22 | HECT and RLD domain containing E3  ubiquitin protein ligase family member 1 | 5.07102 | 4.65922 | 0.411798 | 1.33034 | 0.0053337 | 0.158115 |

| C1orf174 | chr1p36.32 | chromosome 1 open reading frame  174 | 7.18846 | 6.75259 | 0.43587 | 1.35273 | 0.002763 | 0.148407 |
| --- | --- | --- | --- | --- | --- | --- | --- | --- |
| ZNF652 | chr17q21.32 | zinc finger protein 652 | 5.24358 | 4.7263 | 0.517285 | 1.43126 | 0.001932 | 0.146411 |
| RBM25 | chr14q24.3 | RNA binding motif protein 25 | 5.75421 | 5.43035 | 0.323864 | 1.25168 | 0.0180129 | 0.198662 |
| PIK3R1 | chr5q13.1 | phosphoinositide-3-kinase, regulatory  subunit 1 (alpha) | 8.65392 | 8.36996 | 0.283959 | 1.21753 | 0.0126424 | 0.187372 |
| INPP5B | chr1p34 | inositol polyphosphate-5-phosphatase  B | 6.36268 | 6.07883 | 0.283848 | 1.21744 | 0.0126453 | 0.187372 |
| SKP2 | chr5p13 | S-phase kinase-associated protein 2,  E3 ubiquitin protein ligase | 3.52866 | 3.26173 | 0.266935 | 1.20325 | 0.0060119 | 0.161186 |
| DBT | chr1p31 | dihydrolipoamide branched chain  transacylase E2 | 5.77614 | 5.48686 | 0.289281 | 1.22203 | 0.0045348 | 0.154764 |
| IRAK4 | chr12q12 | interleukin 1 receptor associated  kinase 4 | 6.70844 | 6.44066 | 0.267772 | 1.20395 | 0.0058978 | 0.160245 |
| N4BP2 | chr4p14 | NEDD4 binding protein 2 | 4.68601 | 4.3954 | 0.290613 | 1.22316 | 0.0024766 | 0.148407 |
| ORMDL3 | chr17q12 | ORMDL sphingolipid biosynthesis  regulator 3 | 4.40419 | 4.0572 | 0.346989 | 1.2719 | 0.0109049 | 0.181825 |
| TTC5 | chr14q11.2 | tetratricopeptide repeat domain 5 | 6.17025 | 5.89836 | 0.27189 | 1.20739 | 0.0020753 | 0.147757 |
| VSIG1 | chrXq22.3 | V-set and immunoglobulin domain  containing 1 | 4.00004 | 3.64833 | 0.351708 | 1.27607 | 0.00304 | 0.148407 |
| HIST1H3A | chr6p22.2 | histone cluster 1, H3a | 5.35843 | 5.03725 | 0.321177 | 1.24935 | 0.0135399 | 0.189802 |
| CYCS | chr7p15.3 | cytochrome c, somatic | 5.32117 | 4.99186 | 0.329305 | 1.25641 | 0.012019 | 0.186078 |
| PRIM2 | chr6p12-p11.1 | primase, DNA, polypeptide 2 (58kDa) | 3.65318 | 3.3605 | 0.292674 | 1.22491 | 0.0085507 | 0.170624 |
| CELF1 | chr11p11 | CUGBP, Elav-like family member 1 | 5.86002 | 5.53471 | 0.325312 | 1.25294 | 0.0167873 | 0.197728 |
| CASP2 | chr7q34-q35 | caspase 2 | 4.74502 | 4.42669 | 0.318336 | 1.24689 | 0.0144994 | 0.191711 |
| --- | --- | --- | 6.54081 | 6.15839 | 0.382427 | 1.30353 | 0.0019935 | 0.146569 |
| --- | --- | --- | 4.9828 | 4.57258 | 0.410226 | 1.32889 | 0.0065456 | 0.163699 |
| DHX35 | chr20q11.22-q12 | DEAH (Asp-Glu-Ala-His) box  polypeptide 35 | 4.64268 | 4.23919 | 0.403493 | 1.32271 | 0.0073974 | 0.167706 |
| NUMA1 | chr11q13 | nuclear mitotic apparatus protein 1 | 4.66145 | 4.22336 | 0.438097 | 1.35482 | 0.006106 | 0.161446 |
| BCL2L11 | chr2q13 | BCL2-like 11 (apoptosis facilitator) | 4.99707 | 4.5546 | 0.442462 | 1.35892 | 0.0022668 | 0.148407 |
| --- | --- | --- | 5.36468 | 4.97819 | 0.386495 | 1.30721 | 0.0136551 | 0.190437 |
| NDUFS1 | chr2q33-q34 | NADH dehydrogenase (ubiquinone) Fe-  S protein 1, 75kDa (NADH-coenzyme Q reductase) | 4.84541 | 4.44323 | 0.402185 | 1.32151 | 0.0085157 | 0.170624 |
| CAPZA1 | chr1p13.2 | capping protein (actin filament) muscle  Z-line, alpha 1 | 4.80238 | 4.343 | 0.459383 | 1.37495 | 0.0056113 | 0.158791 |
| C16orf72 | chr16p13.2 | chromosome 16 open reading frame  72 | 4.17605 | 3.75248 | 0.423571 | 1.34124 | 0.0046708 | 0.155506 |
| --- | --- | --- | 4.70125 | 4.12106 | 0.580194 | 1.49505 | 0.0012186 | 0.136585 |
| INPP5A | chr10q26.3 | inositol polyphosphate-5-phosphatase  A | 5.61748 | 5.00214 | 0.615345 | 1.53192 | 0.0015011 | 0.142242 |
| SERPINE1 | chr7q22.1 | serpin peptidase inhibitor, clade E  (nexin, plasminogen activator inhibitor type 1), member 1 | 6.41595 | 5.79649 | 0.619452 | 1.53629 | 0.001665 | 0.145076 |
| TGFB1 | chr19q13.1 | transforming growth factor beta 1 | 8.4978 | 8.06239 | 0.435401 | 1.35229 | 0.0179789 | 0.198662 |
| SLA2 | chr20q11.23 | Src-like-adaptor 2 | 6.4498 | 5.96508 | 0.484728 | 1.39932 | 0.0139576 | 0.190686 |
| PREP | chr6q22 | prolyl endopeptidase | 6.07519 | 5.79157 | 0.283619 | 1.21724 | 0.0043762 | 0.152986 |
| YIPF5 | chr5q31.3 | Yip1 domain family member 5 | 7.92808 | 7.60213 | 0.325944 | 1.25348 | 0.0041496 | 0.151685 |
| BHLHE40 | chr3p26 | basic helix-loop-helix family, member  e40 | 7.77456 | 7.4286 | 0.345964 | 1.271 | 0.0081825 | 0.169323 |
| SNRPN /// SNURF | chr15q11.2 /// chr15q12 | small nuclear ribonucleoprotein  polypeptide N /// SNRPN upstream reading frame | 8.73244 | 8.43919 | 0.29326 | 1.22541 | 0.0080959 | 0.169089 |

| NKG7 | chr19q13.41 | natural killer cell granule protein 7 | 10.0263 | 9.71482 | 0.311519 | 1.24101 | 0.0172682 | 0.198289 |
| --- | --- | --- | --- | --- | --- | --- | --- | --- |
| PBXIP1 | chr1q21.3 | pre-B-cell leukemia homeobox  interacting protein 1 | 7.98138 | 7.65857 | 0.322812 | 1.25077 | 0.0095725 | 0.173173 |
| TNFRSF25 | chr1p36.2 | tumor necrosis factor receptor  superfamily, member 25 | 6.83014 | 6.45069 | 0.379446 | 1.30084 | 0.0090851 | 0.172989 |
| DNPH1 | chr6p21.1 | 2'-deoxynucleoside 5'-phosphate N-  hydrolase 1 | 7.71174 | 7.31757 | 0.394168 | 1.31418 | 0.0070029 | 0.165923 |
| ABL1 | chr9q34.1 | ABL proto-oncogene 1, non-receptor  tyrosine kinase | 7.19594 | 6.89578 | 0.300156 | 1.23128 | 0.0061329 | 0.161558 |
| KLF13 | chr15q12 | Kruppel-like factor 13 | 8.33443 | 8.02413 | 0.310303 | 1.23997 | 0.0026055 | 0.148407 |
| ABHD17A | chr19p13.3 | abhydrolase domain containing 17A | 9.52708 | 9.21585 | 0.311236 | 1.24077 | 0.0072303 | 0.167222 |
| IZUMO4 | chr19p13.3 | IZUMO family member 4 | 4.31154 | 3.94242 | 0.369119 | 1.29156 | 0.0025063 | 0.148407 |
| GGA2 | chr16p12 | golgi-associated, gamma adaptin ear containing, ARF binding protein 2 | 8.5953 | 8.2629 | 0.332396 | 1.2591 | 0.0104234 | 0.17894 |
| TNFRSF18 | chr1p36.3 | tumor necrosis factor receptor  superfamily, member 18 | 6.80942 | 6.46081 | 0.348607 | 1.27333 | 0.0169485 | 0.197956 |
| PEX16 | chr11p11.2 | peroxisomal biogenesis factor 16 | 6.58205 | 6.24685 | 0.335192 | 1.26155 | 0.0139617 | 0.190686 |
| GGA2 | chr16p12 | golgi-associated, gamma adaptin ear containing, ARF binding protein 2 | 8.07602 | 7.72388 | 0.352148 | 1.27646 | 0.0172295 | 0.198289 |
| TNFSF14 | chr19p13.3 | tumor necrosis factor (ligand)  superfamily, member 14 | 6.776 | 6.33103 | 0.444966 | 1.36128 | 0.0061825 | 0.161803 |
| IER5L | chr9q34.11 | immediate early response 5-like | 5.80939 | 5.43665 | 0.372743 | 1.29481 | 0.0128373 | 0.187472 |
| --- | --- | --- | 5.19511 | 4.74324 | 0.451868 | 1.36781 | 0.0037336 | 0.150735 |
| MAPK14 | chr6p21.3-p21.2 | mitogen-activated protein kinase 14 | 5.52043 | 5.21235 | 0.308081 | 1.23806 | 0.0164181 | 0.196619 |
| PDE7A | chr8q13 | phosphodiesterase 7A | 7.18202 | 6.86706 | 0.31496 | 1.24398 | 0.0084745 | 0.170624 |
| RAP2A /// RAP2B | chr13q34 /// chr3q25.2 | RAP2A, member of RAS oncogene  family /// RAP2B, member of RAS oncogene family | 6.65087 | 6.31541 | 0.335464 | 1.26178 | 0.0073977 | 0.167706 |
| HAVCR2 | chr5q33.3 | hepatitis A virus cellular receptor 2 | 6.42095 | 6.07998 | 0.340972 | 1.26661 | 0.0111862 | 0.182647 |
| NUDT3 ///  RPS10- NUDT3 | chr6p21 /// chr6p21.2 | nudix hydrolase 3 /// RPS10-NUDT3 readthrough | 6.78422 | 6.48881 | 0.295404 | 1.22723 | 0.0153559 | 0.193809 |
| MOB3A | chr19p13.3 | MOB kinase activator 3A | 6.43293 | 6.07689 | 0.356031 | 1.2799 | 0.0112925 | 0.182785 |
| ORAI2 | chr7q22.1 | ORAI calcium release-activated calcium  modulator 2 | 6.65547 | 6.23393 | 0.421541 | 1.33936 | 0.0033991 | 0.150538 |
| --- | --- | --- | 3.83123 | 3.39022 | 0.441015 | 1.35756 | 0.0046297 | 0.155277 |
| FXYD5 | chr19q13.12 | FXYD domain containing ion transport  regulator 5 | 9.28839 | 8.95881 | 0.329571 | 1.25664 | 0.0180628 | 0.198864 |
| FXYD5 | chr19q13.12 | FXYD domain containing ion transport  regulator 5 | 9.84615 | 9.47412 | 0.372032 | 1.29417 | 0.0140833 | 0.190686 |
| DDI2 | chr1p36.21 | DNA-damage inducible 1 homolog 2 | 8.10032 | 7.66352 | 0.4368 | 1.3536 | 0.0062744 | 0.162624 |
| LAMP1 | chr13q34 | lysosomal-associated membrane  protein 1 | 7.56115 | 7.1019 | 0.459251 | 1.37483 | 0.002552 | 0.148407 |
| TBCD | chr17q25.3 | tubulin folding cofactor D | 4.30383 | 3.89686 | 0.40697 | 1.3259 | 0.0173217 | 0.198289 |
| CIRBP | chr19p13.3 | cold inducible RNA binding protein | 7.11785 | 6.65937 | 0.458476 | 1.37409 | 0.0123866 | 0.187004 |
| SMARCA2 | chr9p22.3 | SWI/SNF related, matrix associated, actin dependent regulator of chromatin, subfamily a, member 2 | 6.40053 | 6.01824 | 0.382283 | 1.3034 | 0.0110274 | 0.181825 |

| ZBTB20 | chr3q13.2 | zinc finger and BTB domain containing  20 | 6.89961 | 6.4072 | 0.492416 | 1.4068 | 0.0041132 | 0.151685 |
| --- | --- | --- | --- | --- | --- | --- | --- | --- |
| SKI | chr1p36.33 | SKI proto-oncogene | 8.13859 | 7.68447 | 0.454116 | 1.36994 | 0.0003921 | 0.111326 |
| ADGRG3 | chr16q21 | adhesion G protein-coupled receptor  G3 | 4.20656 | 3.74195 | 0.464611 | 1.37995 | 0.0009552 | 0.128628 |
| RCAN3 | chr1p36.11 | RCAN family member 3 | 5.06755 | 4.56253 | 0.505015 | 1.41914 | 0.0021014 | 0.147757 |
| CD7 | chr17q25.2-  q25.3 | CD7 molecule | 6.13473 | 5.51388 | 0.620846 | 1.53778 | 0.007714 | 0.168472 |
| CNOT6L | chr4q13.3 | CCR4-NOT transcription complex  subunit 6-like | 5.99635 | 5.53729 | 0.459056 | 1.37464 | 0.0162533 | 0.196072 |
| SERPINB9 | chr6p25 | serpin peptidase inhibitor, clade B  (ovalbumin), member 9 | 6.14916 | 5.54594 | 0.603217 | 1.5191 | 0.006376 | 0.163547 |
| COPA | chr1q23.2 | coatomer protein complex subunit  alpha | 6.28473 | 5.87824 | 0.406489 | 1.32546 | 0.0054309 | 0.158699 |
| FAF1 | chr1p33 | Fas (TNFRSF6) associated factor 1 | 5.06114 | 4.63823 | 0.422917 | 1.34064 | 0.0174725 | 0.198559 |
| LIX1L | chr1q21.1 | limb and CNS expressed 1 like | 5.44465 | 4.93081 | 0.513843 | 1.42785 | 0.0036404 | 0.150674 |
| GLYR1 | chr16p13.3 | glyoxylate reductase 1 homolog  (Arabidopsis) | 4.90609 | 4.38871 | 0.517378 | 1.43135 | 0.0015211 | 0.143188 |
| MGEA5 | chr10q24.1-  q24.3 | meningioma expressed antigen 5  (hyaluronidase) | 5.42094 | 4.90159 | 0.519351 | 1.43331 | 0.0089846 | 0.172413 |
| CASP2 | chr7q34-q35 | caspase 2 | 5.64238 | 5.18647 | 0.455913 | 1.37165 | 0.0065693 | 0.163699 |
| STAG3L1  /// STAG3L2  ///  STAG3L3 | chr7q11.23 | stromal antigen 3-like 1 (pseudogene)  /// stromal antigen 3-like 2 (pseudogene) /// stromal antigen 3- like 3 (pseudogene) | 5.62359 | 5.0977 | 0.525885 | 1.43982 | 0.0053114 | 0.157883 |
| CASP2 | chr7q34-q35 | caspase 2 | 5.8718 | 5.45211 | 0.419684 | 1.33763 | 0.0165782 | 0.196661 |
| CRKL | chr22q11.21 | v-crk avian sarcoma virus CT10  oncogene homolog-like | 5.37685 | 4.79133 | 0.585518 | 1.50058 | 0.0034449 | 0.150538 |
| ANKH | chr5p15.1 | ANKH inorganic pyrophosphate  transport regulator | 6.14619 | 5.70546 | 0.440735 | 1.3573 | 0.0172865 | 0.198289 |
| RETSAT | chr2p11.2 | retinol saturase (all-trans-retinol 13,14-  reductase) | 5.00194 | 4.55153 | 0.450411 | 1.36643 | 0.0175253 | 0.198559 |
| GTF2I | chr7q11.23 | general transcription factor IIi | 6.26193 | 5.78547 | 0.476459 | 1.39132 | 0.0180588 | 0.198864 |
| RASA3 | chr13q34 | RAS p21 protein activator 3 | 6.663 | 6.18622 | 0.476781 | 1.39163 | 0.0064507 | 0.163699 |
| TMEM259 | chr19p13.3 | transmembrane protein 259 | 6.67434 | 6.18373 | 0.490602 | 1.40503 | 0.0167858 | 0.197728 |
| TGFBR2 | chr3p22 | transforming growth factor beta  receptor II | 6.50072 | 6.03273 | 0.467999 | 1.38319 | 0.0116775 | 0.185171 |
| PRKAR1A | chr17q24.2 | protein kinase, cAMP-dependent,  regulatory, type I, alpha | 8.61438 | 8.11823 | 0.496157 | 1.41045 | 0.0158573 | 0.195007 |
| TRAV12-3 | chr14q11 | T cell receptor alpha variable 12-3 | 4.70687 | 4.31382 | 0.393057 | 1.31317 | 0.0152711 | 0.193632 |
| MSI2 | chr17q22 | musashi RNA binding protein 2 | 4.50274 | 4.11751 | 0.385233 | 1.30607 | 0.0171017 | 0.198257 |
| CASP8 | chr2q33-q34 | caspase 8, apoptosis-related cysteine  peptidase | 7.69485 | 7.29714 | 0.397718 | 1.31742 | 0.0092328 | 0.172989 |
| PDCD4 | chr10q24 | programmed cell death 4 (neoplastic transformation inhibitor) | 9.54146 | 9.12836 | 0.4131 | 1.33154 | 0.0153989 | 0.193979 |
| TTC37 | chr5q15 | tetratricopeptide repeat domain 37 | 5.05602 | 4.63464 | 0.421378 | 1.33921 | 0.0064596 | 0.163699 |
| LOC202181 | chr5q35.3 | SUMO-interacting motifs containing 1  pseudogene | 7.04278 | 6.60408 | 0.438699 | 1.35538 | 0.0128434 | 0.187472 |
| TNRC6C | chr17q25.3 | trinucleotide repeat containing 6C | 4.94728 | 4.44782 | 0.499453 | 1.41368 | 0.0099754 | 0.175177 |
| PITHD1 | chr1p36.11 | PITH (C-terminal proteasome-  interacting domain of thioredoxin-like) domain containing 1 | 5.31978 | 4.81772 | 0.502067 | 1.41624 | 0.0065848 | 0.163766 |
| NDRG3 | chr20q11.21-  q11.23 | NDRG family member 3 | 5.98665 | 5.52713 | 0.45952 | 1.37508 | 0.0134998 | 0.189772 |

| TCP11L2 | chr12q23.3 | t-complex 11, testis-specific-like 2 | 5.19745 | 4.71332 | 0.484132 | 1.39874 | 0.0075802 | 0.168472 |
| --- | --- | --- | --- | --- | --- | --- | --- | --- |
| TCP11L2 | chr12q23.3 | t-complex 11, testis-specific-like 2 | 5.89525 | 5.39137 | 0.503883 | 1.41803 | 0.0037974 | 0.150735 |
| NKTR | chr3p22.1 | natural killer cell triggering receptor | 6.36197 | 5.84848 | 0.51349 | 1.4275 | 0.0080718 | 0.169033 |
| ARHGEF7 | chr13q34 | Rho guanine nucleotide exchange  factor 7 | 6.10225 | 5.58513 | 0.517123 | 1.4311 | 0.0091944 | 0.172989 |
| GLCCI1 | chr7p21.3 | glucocorticoid induced 1 | 6.89849 | 6.36563 | 0.532862 | 1.4468 | 0.0023364 | 0.148407 |
| GRK5 | chr10q26.11 | G protein-coupled receptor kinase 5 | 7.00747 | 6.58365 | 0.423822 | 1.34148 | 0.0173847 | 0.198392 |
| GPR132 | chr14q32.3 | G protein-coupled receptor 132 | 6.67081 | 6.1431 | 0.527715 | 1.44164 | 0.0027004 | 0.148407 |
| PLEKHA2 | chr8p11.22 | pleckstrin homology domain containing, family A (phosphoinositide binding specific) member 2 | 7.10768 | 6.57309 | 0.53459 | 1.44853 | 0.0089001 | 0.172413 |
| PIK3IP1 | chr22q12.2 | phosphoinositide-3-kinase interacting  protein 1 | 10.1357 | 9.57307 | 0.562622 | 1.47695 | 0.014752 | 0.192002 |
| LOC102724  229 /// RASA4 /// RASA4B ///  RASA4CP | chr7p13 ///  chr7q22 /// chr7q22.1 | uncharacterized LOC102724229 /// RAS p21 protein activator 4 /// RAS p21 protein activator 4B /// RAS p21  protein activator 4C, pseudogene | 5.71971 | 5.22918 | 0.490528 | 1.40496 | 0.0176033 | 0.198559 |
| IKBKB | chr8p11.2 | inhibitor of kappa light polypeptide gene enhancer in B-cells, kinase beta | 5.92002 | 5.35635 | 0.563671 | 1.47803 | 0.0154898 | 0.194281 |
| --- | --- | --- | 6.22294 | 5.58476 | 0.638184 | 1.55637 | 0.0042752 | 0.152719 |
| WDR6 | chr3p21.31 | WD repeat domain 6 | 4.68454 | 4.22729 | 0.457251 | 1.37292 | 0.0139708 | 0.190686 |
| ZNF549 | chr19q13.43 | zinc finger protein 549 | 3.21281 | 2.76319 | 0.449624 | 1.36568 | 0.0085466 | 0.170624 |
| SESN3 | chr11q21 | sestrin 3 | 4.93605 | 4.45726 | 0.478787 | 1.39357 | 0.0073401 | 0.167706 |
| --- | --- | --- | 4.31046 | 3.6788 | 0.631656 | 1.54934 | 0.0075641 | 0.168472 |
| RBM14 | chr11q13.2 | RNA binding motif protein 14 | 3.87852 | 3.23393 | 0.644593 | 1.5633 | 0.0060265 | 0.161186 |
| TMEM259 | chr19p13.3 | transmembrane protein 259 | 5.67073 | 4.97386 | 0.696871 | 1.62098 | 0.0046698 | 0.155506 |
| --- | --- | --- | 5.86687 | 5.44125 | 0.425625 | 1.34315 | 0.0129053 | 0.187472 |
| LOC105371  804 /// LRRC37A4P | chr17q21.31 | leucine-rich repeat-containing protein  37A3 pseudogene /// leucine rich repeat containing 37, member A4, pseudogene | 4.39233 | 4.0296 | 0.362739 | 1.28586 | 0.0141086 | 0.190686 |
| RDH13 | chr19q13.42 | retinol dehydrogenase 13 (all-trans/9-  cis) | 4.28872 | 3.88846 | 0.400258 | 1.31974 | 0.0164258 | 0.196619 |
| --- | --- | --- | 6.71749 | 6.34147 | 0.376016 | 1.29775 | 0.0182751 | 0.199849 |
| --- | --- | --- | 4.59748 | 4.08054 | 0.51694 | 1.43092 | 0.0051883 | 0.157506 |
| FBXO32 | chr8q24.13 | F-box protein 32 | 4.62852 | 4.26677 | 0.361752 | 1.28499 | 0.0092815 | 0.172989 |
| --- | --- | --- | 5.83346 | 5.45782 | 0.375641 | 1.29742 | 0.0140926 | 0.190686 |
| --- | --- | --- | 5.28439 | 4.7639 | 0.520488 | 1.43444 | 0.0007226 | 0.128628 |
| --- | --- | --- | 6.50955 | 5.83824 | 0.671305 | 1.59251 | 0.0014213 | 0.139801 |
| --- | --- | --- | 4.88235 | 4.38202 | 0.500329 | 1.41454 | 0.0023979 | 0.148407 |
| LOC102724  985 ///  PDXDC1 | chr16p13.11 | pyridoxal-dependent decarboxylase domain-containing protein 1 /// pyridoxal-dependent decarboxylase  domain containing 1 | 4.73197 | 4.30151 | 0.430464 | 1.34767 | 0.0037859 | 0.150735 |
| CLASP2 | chr3p22.3 | cytoplasmic linker associated protein 2 | 4.65955 | 4.15993 | 0.49962 | 1.41384 | 0.0013028 | 0.137103 |
| ARHGEF7 | chr13q34 | Rho guanine nucleotide exchange  factor 7 | 3.22702 | 2.87295 | 0.354069 | 1.27816 | 0.0180028 | 0.198662 |
| --- | --- | --- | 9.02336 | 8.67481 | 0.348552 | 1.27328 | 0.0073156 | 0.167509 |

| --- | --- | --- | 6.85065 | 6.49377 | 0.356884 | 1.28066 | 0.0176739 | 0.198559 |
| --- | --- | --- | --- | --- | --- | --- | --- | --- |
| HIST1H2BG | chr6p22.2 | histone cluster 1, H2bg | 7.62349 | 7.26052 | 0.362966 | 1.28607 | 0.0044237 | 0.153634 |
| --- | --- | --- | 8.46649 | 8.07888 | 0.387608 | 1.30822 | 0.003807 | 0.150735 |
| PRKD3 | chr2p21 | protein kinase D3 | 5.24973 | 4.90253 | 0.347196 | 1.27209 | 0.0144188 | 0.191711 |
| --- | --- | --- | 6.05807 | 5.70804 | 0.350035 | 1.27459 | 0.0123951 | 0.187004 |
| --- | --- | --- | 6.61937 | 6.22092 | 0.398449 | 1.31809 | 0.0029852 | 0.148407 |
| --- | --- | --- | 5.30722 | 4.93571 | 0.37151 | 1.29371 | 0.014167 | 0.191112 |
| SPDYE2 | chr7q22.1 | speedy/RINGO cell cycle regulator  family member E2 | 6.20491 | 5.77263 | 0.432283 | 1.34937 | 0.0124544 | 0.187036 |
| CCZ1B | chr7p22.1 | CCZ1 homolog B, vacuolar protein trafficking and biogenesis associated | 6.22126 | 5.7792 | 0.44206 | 1.35854 | 0.0130331 | 0.18817 |
| PLEKHA2 | chr8p11.22 | pleckstrin homology domain containing, family A (phosphoinositide binding specific) member 2 | 7.69978 | 7.1983 | 0.501481 | 1.41567 | 0.0080444 | 0.168682 |
| EXOC4 | chr7q31 | exocyst complex component 4 | 5.96919 | 5.4707 | 0.498493 | 1.41274 | 0.0038789 | 0.151023 |
| --- | --- | --- | 7.19617 | 6.73047 | 0.465703 | 1.38099 | 0.0080926 | 0.169089 |
| LOC100996  741 /// LOC105371  224 /// LOC105371  420 ///  LOC388692 | chr1q21.2 | uncharacterized LOC100996741 /// uncharacterized LOC105371224 /// uncharacterized LOC105371420 /// uncharacterized LOC388692 | 5.85519 | 5.35667 | 0.498519 | 1.41276 | 0.0076729 | 0.168472 |
| NDRG3 | chr20q11.21-  q11.23 | NDRG family member 3 | 5.93363 | 5.42958 | 0.504042 | 1.41818 | 0.0036453 | 0.150674 |
| FXYD5 ///  LOC100127 972 | chr19q13.12 | FXYD domain containing ion transport  regulator 5 /// uncharacterized LOC100127972 | 6.04679 | 5.5186 | 0.528186 | 1.44211 | 0.0145105 | 0.191711 |
| KATNBL1 | chr15q14 | katanin p80 subunit B-like 1 | 5.52323 | 4.98542 | 0.537813 | 1.45177 | 0.0064359 | 0.163699 |
| --- | --- | --- | 4.70458 | 4.16568 | 0.538904 | 1.45287 | 0.0028272 | 0.148407 |
| LRCH3 | chr3q29 | leucine-rich repeats and calponin homology (CH) domain containing 3 | 7.01354 | 6.46073 | 0.552802 | 1.46693 | 0.0013799 | 0.138491 |
| CBFA2T2 | chr20q11 | core-binding factor, runt domain,  alpha subunit 2; translocated to, 2 | 5.81222 | 5.25012 | 0.562104 | 1.47642 | 0.0021565 | 0.147979 |
| TMEM165 | chr4q12 | transmembrane protein 165 | 8.66561 | 8.28417 | 0.38144 | 1.30264 | 0.0169551 | 0.197956 |
| DLEU2 | chr13q14.3 | deleted in lymphocytic leukemia 2  (non-protein coding) | 4.26868 | 3.86156 | 0.407119 | 1.32604 | 0.0175853 | 0.198559 |
| LINC-PINT | chr7q32.3 | long intergenic non-protein coding  RNA, p53 induced transcript | 5.48296 | 5.01219 | 0.47077 | 1.38585 | 0.0031115 | 0.148407 |
| CASP2 | chr7q34-q35 | caspase 2 | 5.93101 | 5.45181 | 0.4792 | 1.39397 | 0.0047004 | 0.155506 |
| PIK3IP1 | chr22q12.2 | phosphoinositide-3-kinase interacting  protein 1 | 4.99979 | 4.51995 | 0.479837 | 1.39459 | 0.0097177 | 0.173861 |
| --- | --- | --- | 7.22902 | 6.82025 | 0.40877 | 1.32755 | 0.0041885 | 0.151955 |
| --- | --- | --- | 5.67913 | 5.22306 | 0.456074 | 1.3718 | 0.0036535 | 0.150674 |
| VHL | chr3p25.3 | von Hippel-Lindau tumor suppressor, E3 ubiquitin protein ligase | 6.11792 | 5.68973 | 0.428185 | 1.34554 | 0.0068253 | 0.164807 |
| --- | --- | --- | 7.35667 | 6.85295 | 0.503718 | 1.41786 | 0.0028462 | 0.148407 |
| --- | --- | --- | 6.00808 | 5.57049 | 0.437596 | 1.35435 | 0.013418 | 0.189772 |
| ZNF264 | chr19q13.4 | zinc finger protein 264 | 6.60121 | 6.11186 | 0.489352 | 1.40381 | 0.0071645 | 0.166896 |

| ANKRD10  /// ANKRD10- IT1 | chr13q34 | ankyrin repeat domain 10 /// ANKRD10 intronic transcript 1 | 7.91802 | 7.45705 | 0.460969 | 1.37647 | 0.0089264 | 0.172413 |
| --- | --- | --- | --- | --- | --- | --- | --- | --- |
| --- | --- | --- | 5.26003 | 4.68979 | 0.57024 | 1.48477 | 0.0056309 | 0.158791 |
| OGDH | chr7p14-p13 | oxoglutarate (alpha-ketoglutarate)  dehydrogenase (lipoamide) | 4.62099 | 4.16387 | 0.457123 | 1.3728 | 0.004983 | 0.157506 |
| --- | --- | --- | 4.06868 | 3.51834 | 0.550338 | 1.46443 | 0.0017728 | 0.145076 |
| DCAF7 | chr17q23.3 | DDB1 and CUL4 associated factor 7 | 5.11716 | 4.75771 | 0.359445 | 1.28293 | 0.0071161 | 0.166748 |
| --- | --- | --- | 5.24345 | 4.82096 | 0.422486 | 1.34023 | 0.0115769 | 0.184851 |
| CAMKK2 | chr12q24.2 | calcium/calmodulin-dependent protein  kinase kinase 2, beta | 5.0431 | 4.7507 | 0.292409 | 1.22468 | 0.0139577 | 0.190686 |
| PRRC2B | chr9q34.13 | proline-rich coiled-coil 2B | 5.31885 | 4.97396 | 0.344895 | 1.27006 | 0.0065694 | 0.163699 |
| METTL12 | chr11q12.3 | methyltransferase like 12 | 5.89248 | 5.51386 | 0.378624 | 1.3001 | 0.0016143 | 0.145076 |
| RBL1 | chr20q11.2 | retinoblastoma-like 1 | 3.75929 | 3.35767 | 0.401622 | 1.32099 | 0.0037373 | 0.150735 |
| DLG1 | chr3q29 | discs, large homolog 1 (Drosophila) | 5.92567 | 5.45022 | 0.475457 | 1.39036 | 0.0050348 | 0.157506 |
| LOC101927  451 | --- | uncharacterized LOC101927451 | 5.93171 | 5.55186 | 0.37985 | 1.30121 | 0.0159644 | 0.195587 |
| --- | --- | --- | 3.9928 | 3.61867 | 0.374128 | 1.29606 | 0.017924 | 0.198662 |
| --- | --- | --- | 5.33578 | 4.92595 | 0.409826 | 1.32853 | 0.0176854 | 0.198559 |
| --- | --- | --- | 5.73917 | 5.33703 | 0.402137 | 1.32146 | 0.0160481 | 0.195587 |
| MSI2 | chr17q22 | musashi RNA binding protein 2 | 5.89007 | 5.46112 | 0.42895 | 1.34625 | 0.0162834 | 0.196072 |
| --- | --- | --- | 3.68817 | 3.27087 | 0.417305 | 1.33543 | 0.0108266 | 0.181472 |
| --- | --- | --- | 5.92562 | 5.57418 | 0.351445 | 1.27584 | 0.0043028 | 0.152719 |
| BMS1P5 /// LOC102724 455 | chr10q11.22 | BMS1 ribosome biogenesis factor pseudogene 5 /// uncharacterized LOC102724455 | 5.7893 | 5.39488 | 0.394418 | 1.31441 | 0.0065628 | 0.163699 |
| --- | --- | --- | 6.89227 | 6.49908 | 0.393187 | 1.31329 | 0.003941 | 0.151256 |
| --- | --- | --- | 5.29918 | 4.87367 | 0.4255 | 1.34304 | 0.0059835 | 0.160989 |
| --- | --- | --- | 4.79384 | 4.42817 | 0.365666 | 1.28848 | 0.0179282 | 0.198662 |
| --- | --- | --- | 4.10121 | 3.722 | 0.379212 | 1.30063 | 0.0098608 | 0.174971 |
| LYST | chr1q42.1-q42.2 | lysosomal trafficking regulator | 5.4147 | 5.06691 | 0.347789 | 1.27261 | 0.0113165 | 0.182872 |
| GOLGA8A  /// GOLGA8B | chr15q11.2 /// chr15q14 | golgin A8 family, member A /// golgin A8 family, member B | 8.03708 | 7.67734 | 0.35974 | 1.28319 | 0.0075854 | 0.168472 |
| --- | --- | --- | 4.93295 | 4.47127 | 0.461677 | 1.37714 | 0.0047961 | 0.156034 |
| STAG3L1  /// STAG3L3 | chr7q11.23 | stromal antigen 3-like 1 (pseudogene)  /// stromal antigen 3-like 3 (pseudogene) | 6.17463 | 5.78942 | 0.385207 | 1.30605 | 0.0121058 | 0.186382 |
| ABHD18 | chr4q28.2 | abhydrolase domain containing 18 | 5.68286 | 5.24478 | 0.438078 | 1.3548 | 0.0102615 | 0.177837 |
| --- | --- | --- | 5.24127 | 4.80555 | 0.435725 | 1.35259 | 0.006108 | 0.161446 |
| --- | --- | --- | 5.96774 | 5.50079 | 0.466955 | 1.38219 | 0.0031128 | 0.148407 |
| --- | --- | --- | 4.7902 | 4.30422 | 0.485975 | 1.40053 | 0.005071 | 0.157506 |
| CCDC91 | chr12p11.22 | coiled-coil domain containing 91 | 5.95234 | 5.45167 | 0.500668 | 1.41487 | 0.005928 | 0.160604 |
| --- | --- | --- | 4.04913 | 3.73726 | 0.311869 | 1.24132 | 0.0166182 | 0.196733 |
| TIAM1 | chr21q22.11 | T-cell lymphoma invasion and  metastasis 1 | 5.22821 | 4.80968 | 0.418529 | 1.33656 | 0.0038361 | 0.150786 |
| RAB35 | chr12q24.31 | RAB35, member RAS oncogene family | 4.71644 | 4.2952 | 0.421241 | 1.33908 | 0.0048491 | 0.156541 |
| --- | --- | --- | 3.54216 | 3.19401 | 0.348152 | 1.27293 | 0.0118863 | 0.185824 |
| --- | --- | --- | 4.05151 | 3.61185 | 0.439656 | 1.35628 | 0.0052607 | 0.157676 |

| SMDT1 | chr22q13.2 | single-pass membrane protein with  aspartate-rich tail 1 | 3.91103 | 3.58367 | 0.327367 | 1.25472 | 0.0151352 | 0.193396 |
| --- | --- | --- | --- | --- | --- | --- | --- | --- |
| POLH | chr6p21.1 | polymerase (DNA directed), eta | 4.58973 | 4.19501 | 0.39472 | 1.31469 | 0.009223 | 0.172989 |
| TGDS | chr13q32.1 | TDP-glucose 4,6-dehydratase | 3.45438 | 3.12058 | 0.333807 | 1.26034 | 0.0072659 | 0.167291 |
| --- | --- | --- | 5.44543 | 5.07629 | 0.369141 | 1.29158 | 0.0085591 | 0.170624 |
| SEC63 | chr6q21 | SEC63 homolog, protein translocation  regulator | 3.23885 | 2.81419 | 0.424667 | 1.34226 | 0.0007768 | 0.128628 |
| APBB1IP | chr10p12.1 | amyloid beta (A4) precursor protein-  binding, family B, member 1  interacting protein | 4.79555 | 4.33557 | 0.459976 | 1.37552 | 0.005416 | 0.158699 |
| FAM159A | chr1p32.3 | family with sequence similarity 159,  member A | 4.01391 | 3.64627 | 0.367632 | 1.29023 | 0.0160149 | 0.195587 |
| --- | --- | --- | 3.66091 | 3.25949 | 0.401425 | 1.32081 | 0.0066897 | 0.164139 |
| RBM14-  RBM4 /// RBM4 | chr11q /// chr11q13 | RBM14-RBM4 readthrough /// RNA binding motif protein 4 | 3.43344 | 3.05566 | 0.377783 | 1.29934 | 0.0093063 | 0.173008 |
| PHC3 | chr3q26.2 | polyhomeotic homolog 3 (Drosophila) | 5.05647 | 4.57174 | 0.484727 | 1.39932 | 0.0036086 | 0.150674 |
| --- | --- | --- | 3.44101 | 2.98048 | 0.460527 | 1.37604 | 0.0007515 | 0.128628 |
| ZNF451 | chr6p12.1 | zinc finger protein 451 | 7.45388 | 6.9443 | 0.509578 | 1.42363 | 0.0003699 | 0.111326 |
| HCG11 | chr6p22.2 | HLA complex group 11 (non-protein  coding) | 2.72732 | 2.42296 | 0.304358 | 1.23487 | 0.0156246 | 0.194408 |
| SNTB1 | chr8q23-q24 | syntrophin, beta 1 (dystrophin-  associated protein A1, 59kDa, basic component 1) | 4.42041 | 4.15365 | 0.266755 | 1.2031 | 0.0171627 | 0.198289 |
| --- | --- | --- | 5.64747 | 5.35476 | 0.292705 | 1.22493 | 0.0115355 | 0.184417 |
| --- | --- | --- | 5.08704 | 4.78133 | 0.305707 | 1.23602 | 0.0031111 | 0.148407 |
| --- | --- | --- | 3.11278 | 2.83649 | 0.276293 | 1.21108 | 0.0008343 | 0.128628 |
| RBSN | chr3p25.1 | rabenosyn, RAB effector | 5.52475 | 5.22936 | 0.295383 | 1.22721 | 0.0007001 | 0.128628 |
| BMS1P5 | chr10q11.22 | BMS1 ribosome biogenesis factor  pseudogene 5 | 3.05134 | 2.73949 | 0.311843 | 1.24129 | 0.0044391 | 0.153634 |
| --- | --- | --- | 3.00765 | 2.67767 | 0.329979 | 1.257 | 0.0004103 | 0.112085 |
| --- | --- | --- | 3.09325 | 2.76115 | 0.332104 | 1.25885 | 0.0037258 | 0.150735 |
| --- | --- | --- | 5.66996 | 5.35904 | 0.310919 | 1.2405 | 0.0077244 | 0.168472 |
| LOC401320 | chr7p15.1 | uncharacterized LOC401320 | 5.70568 | 5.3965 | 0.309176 | 1.239 | 0.0117345 | 0.185472 |
| --- | --- | --- | 4.78592 | 4.42375 | 0.362169 | 1.28536 | 0.0092574 | 0.172989 |
| DLD | chr7q31-q32 | dihydrolipoamide dehydrogenase | 6.17569 | 5.77812 | 0.397569 | 1.31729 | 0.0021833 | 0.147979 |
| C18orf15 | chr18p11.21 | chromosome 18 open reading frame  15 | 3.18479 | 2.90534 | 0.279457 | 1.21374 | 0.0022004 | 0.148212 |
| --- | --- | --- | 6.3415 | 6.00323 | 0.338272 | 1.26424 | 0.0055544 | 0.158791 |
| --- | --- | --- | 3.53246 | 3.26296 | 0.269499 | 1.20539 | 0.0165441 | 0.196619 |
| --- | --- | --- | 6.6584 | 6.38763 | 0.270763 | 1.20645 | 0.0044905 | 0.154031 |
| CDS2 | chr20p13 | CDP-diacylglycerol synthase 2 | 5.29104 | 5.00621 | 0.284823 | 1.21826 | 0.0120622 | 0.186203 |
| LRPPRC | chr2p21 | leucine-rich pentatricopeptide repeat  containing | 6.6458 | 6.3693 | 0.276502 | 1.21125 | 0.0099307 | 0.174971 |
| USP34 | chr2p15 | ubiquitin specific peptidase 34 | 4.96598 | 4.65257 | 0.313404 | 1.24264 | 0.0081621 | 0.169323 |
| HECTD1 | chr14q12 | HECT domain containing E3 ubiquitin  protein ligase 1 | 5.84651 | 5.58159 | 0.264919 | 1.20157 | 0.0046572 | 0.155506 |

| LOC105369  244 /// LOC105369  248 /// NPIPB3 /// NPIPB4 /// NPIPB5 /// SMG1P1  ///  SMG1P3 | chr16p12.2 | putative uncharacterized protein LOC641298 /// nuclear pore complex- interacting protein family member B5- like /// nuclear pore complex interacting protein family, member B3  /// nuclear pore complex interacting protein family, member B4 /// nuclear pore complex interacting protein family, member B5 /// SMG1  pseudogene 1 /// SMG1 pseudogene 3 | 9.25479 | 8.97244 | 0.282351 | 1.21618 | 0.0027547 | 0.148407 |
| --- | --- | --- | --- | --- | --- | --- | --- | --- |
| EXOC7 | chr17q25.1 | exocyst complex component 7 | 5.46311 | 5.16121 | 0.301902 | 1.23277 | 0.0014714 | 0.141488 |
| ADAT1 | chr16q23.1 | adenosine deaminase, tRNA-specific 1 | 6.52922 | 6.20777 | 0.321445 | 1.24958 | 0.0051814 | 0.157506 |
| ACTR2 | chr2p14 | ARP2 actin-related protein 2 homolog  (yeast) | 6.06947 | 5.80281 | 0.266659 | 1.20302 | 0.0104004 | 0.17879 |
| --- | --- | --- | 6.20848 | 5.87312 | 0.335356 | 1.26169 | 0.0079381 | 0.168472 |
| HP08777  /// SLC16A1- AS1 | chr1p13.2 | uncharacterized LOC105378912 /// SLC16A1 antisense RNA 1 | 3.88734 | 3.59179 | 0.295552 | 1.22735 | 0.0083023 | 0.169437 |
| CDC42-IT1 | chr1p36.12 | CDC42 intronic transcript 1 | 5.72675 | 5.37483 | 0.351925 | 1.27626 | 0.0053522 | 0.158323 |
| ZNF514 | chr2q11.1 | zinc finger protein 514 | 4.9524 | 4.57448 | 0.377917 | 1.29946 | 0.0028162 | 0.148407 |
| ZNF345 | chr19q13.12 | zinc finger protein 345 | 4.80731 | 4.43256 | 0.374748 | 1.29661 | 0.0005222 | 0.123548 |
| --- | --- | --- | 5.21275 | 4.77582 | 0.436929 | 1.35372 | 0.0005715 | 0.125351 |
| --- | --- | --- | 2.32176 | 2.0395 | 0.282254 | 1.21609 | 0.0054518 | 0.158789 |
| CLN8 | chr8p23 | ceroid-lipofuscinosis, neuronal 8 | 5.83876 | 5.52168 | 0.317083 | 1.24581 | 0.0039328 | 0.151256 |
| --- | --- | --- | 4.15469 | 3.77916 | 0.375526 | 1.29731 | 0.0009354 | 0.128628 |
| --- | --- | --- | 3.36913 | 2.97457 | 0.394561 | 1.31454 | 0.0003161 | 0.111326 |
| --- | --- | --- | 4.91412 | 4.50263 | 0.411489 | 1.33006 | 0.0001765 | 0.111326 |
| RANBP9 | chr6p23 | RAN binding protein 9 | 5.47072 | 5.14759 | 0.32313 | 1.25104 | 0.0005615 | 0.124545 |
| UBXN7 | chr3q29 | UBX domain protein 7 | 7.39573 | 6.96839 | 0.427337 | 1.34475 | 0.0002937 | 0.111326 |
| HGSNAT | chr8p11.1 | heparan-alpha-glucosaminide N-  acetyltransferase | 4.95684 | 4.59815 | 0.358691 | 1.28226 | 0.0025378 | 0.148407 |
| SUZ12 | chr17q11.2 | SUZ12 polycomb repressive complex 2  subunit | 4.20318 | 3.7737 | 0.429476 | 1.34674 | 0.0009087 | 0.128628 |
| MDM2 | chr12q14.3-q15 | MDM2 proto-oncogene, E3 ubiquitin  protein ligase | 5.39294 | 4.894 | 0.498945 | 1.41318 | 0.0001413 | 0.111326 |
| BMP2K | chr4q21.21 | BMP2 inducible kinase | 3.78836 | 3.39796 | 0.390397 | 1.31075 | 0.0021787 | 0.147979 |
| BCAS3 | chr17q23 | breast carcinoma amplified sequence 3 | 5.04447 | 4.75088 | 0.293588 | 1.22569 | 0.0045312 | 0.154764 |
| ABCC1 | chr16p13.1 | ATP binding cassette subfamily C  member 1 | 6.3552 | 6.06637 | 0.288825 | 1.22165 | 0.0007204 | 0.128628 |
| DDX19A | chr16q22.1 | DEAD (Asp-Glu-Ala-Asp) box  polypeptide 19A | 6.47359 | 6.17003 | 0.303566 | 1.23419 | 0.0007409 | 0.128628 |
| PPP1R3B | chr8p23.1 | protein phosphatase 1, regulatory  subunit 3B | 4.11765 | 3.79445 | 0.323209 | 1.25111 | 0.0002922 | 0.111326 |
| AFG3L1P | chr16q24.3 | AFG3-like AAA ATPase 1, pseudogene | 4.49961 | 4.21273 | 0.28688 | 1.22 | 0.0036383 | 0.150674 |
| KLHL36 | chr16q24.1 | kelch-like family member 36 | 6.72932 | 6.43286 | 0.296457 | 1.22812 | 0.006289 | 0.162624 |
| ZNF790-  AS1 | chr19q13.13 | ZNF790 antisense RNA 1 | 3.69663 | 3.36207 | 0.334562 | 1.26099 | 0.000283 | 0.111326 |
| TSC2 | chr16p13.3 | tuberous sclerosis 2 | 3.72566 | 3.45813 | 0.267531 | 1.20375 | 0.0109012 | 0.181825 |
| --- | --- | --- | 5.88443 | 5.5759 | 0.308533 | 1.23845 | 0.0029356 | 0.148407 |
| PDLIM7 | chr5q35.3 | PDZ and LIM domain 7 (enigma) | 5.18213 | 4.82355 | 0.358576 | 1.28216 | 0.0009411 | 0.128628 |

| --- | --- | --- | 6.66201 | 6.38751 | 0.2745 | 1.20957 | 0.014777 | 0.192002 |
| --- | --- | --- | --- | --- | --- | --- | --- | --- |
| SLC1A4 | chr2p15-p13 | solute carrier family 1  (glutamate/neutral amino acid transporter), member 4 | 5.81425 | 5.54926 | 0.264989 | 1.20163 | 0.0034742 | 0.150538 |
| ANKRD46  /// GAPDHP62 | chr8q22.2 | ankyrin repeat domain 46 /// glyceraldehyde 3 phosphate  dehydrogenase pseudogene 62 | 6.37815 | 6.10149 | 0.276656 | 1.21138 | 0.0026614 | 0.148407 |
| FLT1 | chr13q12 | fms-related tyrosine kinase 1 | 7.40627 | 7.1086 | 0.297669 | 1.22916 | 0.0101331 | 0.176684 |
| --- | --- | --- | 6.76488 | 6.46097 | 0.303907 | 1.23448 | 0.0025842 | 0.148407 |
| CCDC90B | chr11q14.1 | coiled-coil domain containing 90B | 5.86446 | 5.53505 | 0.329416 | 1.2565 | 0.0016809 | 0.145076 |
| NAPEPLD | chr7q22.1 | N-acyl phosphatidylethanolamine  phospholipase D | 3.65026 | 3.36409 | 0.286165 | 1.21939 | 0.0166292 | 0.196733 |
| ACSL4 | chrXq22.3-q23 | acyl-CoA synthetase long-chain family  member 4 | 4.61346 | 4.17613 | 0.43733 | 1.3541 | 0.0008481 | 0.128628 |
| MSI2 | chr17q22 | musashi RNA binding protein 2 | 3.38975 | 2.97663 | 0.413124 | 1.33157 | 0.001174 | 0.134826 |
| --- | --- | --- | 4.44292 | 4.03093 | 0.411993 | 1.33052 | 0.0019057 | 0.14618 |
| CASK | chrXp11.4 | calcium/calmodulin-dependent serine protein kinase (MAGUK family) | 3.90554 | 3.44436 | 0.461177 | 1.37666 | 0.0006546 | 0.128628 |
| MAVS | chr20p13 | mitochondrial antiviral signaling  protein | 4.47587 | 4.16628 | 0.309593 | 1.23936 | 0.0150372 | 0.193368 |
| --- | --- | --- | 4.96116 | 4.69684 | 0.264316 | 1.20107 | 0.0170118 | 0.19809 |
| CHD9 | chr16q12.2 | chromodomain helicase DNA binding  protein 9 | 5.6958 | 5.36218 | 0.333624 | 1.26018 | 0.0051836 | 0.157506 |
| --- | --- | --- | 4.09806 | 3.75608 | 0.341988 | 1.2675 | 0.0138064 | 0.190686 |
| PCMTD2 | chr20q13.33 | protein-L-isoaspartate (D-aspartate) O-  methyltransferase domain containing 2 | 4.7187 | 4.37642 | 0.342277 | 1.26776 | 0.0157579 | 0.194682 |
| --- | --- | --- | 3.18967 | 2.92245 | 0.267224 | 1.20349 | 0.0152489 | 0.193632 |
| --- | --- | --- | 5.46267 | 5.14164 | 0.321029 | 1.24922 | 0.0075652 | 0.168472 |
| --- | --- | --- | 4.00812 | 3.6526 | 0.355516 | 1.27944 | 0.0020632 | 0.147757 |
| MAP3K2 | chr2q14.3 | mitogen-activated protein kinase  kinase kinase 2 | 3.1463 | 2.8006 | 0.345701 | 1.27077 | 0.0078973 | 0.168472 |
| SPG11 | chr15q14 | spastic paraplegia 11 (autosomal  recessive) | 2.84014 | 2.56719 | 0.272947 | 1.20827 | 0.01654 | 0.196619 |
| PCBD2 | chr5q31.1 | pterin-4 alpha-carbinolamine  dehydratase/dimerization cofactor of hepatocyte nuclear factor 1 alpha (TCF1) 2 | 3.82306 | 3.46132 | 0.361741 | 1.28498 | 0.0123229 | 0.187004 |
| MKL1 | chr22q13 | megakaryoblastic leukemia  (translocation) 1 | 5.6124 | 5.29173 | 0.320664 | 1.24891 | 0.0071103 | 0.166748 |
| RDH11 | chr14q24.1 | retinol dehydrogenase 11 (all-trans/9-  cis/11-cis) | 4.1146 | 3.77141 | 0.343186 | 1.26856 | 0.0028749 | 0.148407 |
| CLN8 | chr8p23 | ceroid-lipofuscinosis, neuronal 8 | 5.7792 | 5.32013 | 0.459063 | 1.37465 | 0.0009023 | 0.128628 |
| AKAP11 | chr13q14.11 | A kinase (PRKA) anchor protein 11 | 6.19178 | 5.75454 | 0.437244 | 1.35401 | 0.000106 | 0.111326 |
| SKIL | chr3q26 | SKI-like proto-oncogene | 8.64913 | 8.14545 | 0.503675 | 1.41782 | 0.0001313 | 0.111326 |
| CHD1L | chr1q12 | chromodomain helicase DNA binding  protein 1-like | 5.94076 | 5.64642 | 0.294343 | 1.22633 | 0.0073995 | 0.167706 |
| WDFY2 | chr13q14.3 | WD repeat and FYVE domain  containing 2 | 5.70987 | 5.39006 | 0.319807 | 1.24816 | 0.0050089 | 0.157506 |
| TSPAN31 | chr12q13.3 | tetraspanin 31 | 6.41184 | 6.14426 | 0.267583 | 1.20379 | 0.0079241 | 0.168472 |
| SLC35E1 | chr19p13.11 | solute carrier family 35, member E1 | 6.73762 | 6.46317 | 0.274448 | 1.20953 | 0.001318 | 0.137103 |
| CA5BP1 | chrXp22.2 | carbonic anhydrase VB pseudogene 1 | 5.42006 | 5.10511 | 0.314956 | 1.24397 | 0.0127264 | 0.187417 |
| NHLRC3 | chr13q13.3 | NHL repeat containing 3 | 5.46512 | 5.18818 | 0.27694 | 1.21162 | 0.009255 | 0.172989 |

| --- | --- | --- | 5.02668 | 4.71747 | 0.309211 | 1.23903 | 0.0038229 | 0.150786 |
| --- | --- | --- | --- | --- | --- | --- | --- | --- |
| HM13 | chr20q11.21 | histocompatibility (minor) 13 | 5.25422 | 4.91752 | 0.336703 | 1.26287 | 0.0104505 | 0.178983 |
| KATNBL1 | chr15q14 | katanin p80 subunit B-like 1 | 4.77137 | 4.47155 | 0.299819 | 1.23099 | 0.010495 | 0.179155 |
| AGAP9 /// BMS1P5 /// LOC102724 455 | chr10q11.22 | ArfGAP with GTPase domain, ankyrin  repeat and PH domain 9 /// BMS1 ribosome biogenesis factor pseudogene 5 /// uncharacterized LOC102724455 | 4.72466 | 4.33715 | 0.387517 | 1.30814 | 0.0043487 | 0.152986 |
| --- | --- | --- | 5.28609 | 4.96155 | 0.324544 | 1.25227 | 0.0146648 | 0.191883 |
| --- | --- | --- | 4.85485 | 4.43329 | 0.421559 | 1.33937 | 0.0021055 | 0.147757 |
| LOC105373  495 | --- | uncharacterized LOC105373495 | 3.98016 | 3.64222 | 0.337947 | 1.26396 | 0.0151469 | 0.193396 |
| --- | --- | --- | 4.4577 | 4.15025 | 0.307451 | 1.23752 | 0.0111382 | 0.182397 |
| --- | --- | --- | 4.05504 | 3.7043 | 0.350748 | 1.27522 | 0.0044736 | 0.153894 |
| C1orf43 | chr1q21.2 | chromosome 1 open reading frame 43 | 4.10999 | 3.62985 | 0.480138 | 1.39488 | 0.001944 | 0.146411 |
| --- | --- | --- | 5.36096 | 4.76711 | 0.593845 | 1.50926 | 0.0001694 | 0.111326 |
| RABL2A /// RABL2B | chr22q13.33 /// chr2q13 | RAB, member of RAS oncogene family-  like 2A /// RAB, member of RAS  oncogene family-like 2B | 5.24701 | 4.72016 | 0.526849 | 1.44078 | 0.0011554 | 0.134826 |
| --- | --- | --- | 5.67958 | 5.0987 | 0.580879 | 1.49576 | 0.0028626 | 0.148407 |
| --- | --- | --- | 5.45396 | 4.97339 | 0.480561 | 1.39529 | 0.0114453 | 0.183744 |
| --- | --- | --- | 5.96445 | 5.36749 | 0.596959 | 1.51252 | 0.0038111 | 0.150746 |
| --- | --- | --- | 5.34265 | 4.68294 | 0.659708 | 1.57976 | 0.0032242 | 0.149592 |
| --- | --- | --- | 6.45053 | 5.90355 | 0.546986 | 1.46103 | 0.005056 | 0.157506 |
| SNRK | chr3p22.1 | SNF related kinase | 5.65774 | 5.04775 | 0.609991 | 1.52625 | 0.0032097 | 0.149592 |
| CUL4A | chr13q34 | cullin 4A | 5.99227 | 5.38953 | 0.602738 | 1.5186 | 0.0050345 | 0.157506 |
| NFATC3 | chr16q22.2 | nuclear factor of activated T-cells, cytoplasmic, calcineurin-dependent 3 | 5.86346 | 5.24077 | 0.622684 | 1.53974 | 0.0034256 | 0.150538 |
| ZNF207 | chr17q11.2 | zinc finger protein 207 | 8.58248 | 8.00735 | 0.575126 | 1.48981 | 0.016451 | 0.196619 |
| --- | --- | --- | 6.34745 | 5.68994 | 0.657509 | 1.57736 | 0.0052191 | 0.157641 |
| ZCCHC11 | chr1p32.3 | zinc finger, CCHC domain containing 11 | 4.66001 | 4.04029 | 0.619716 | 1.53657 | 0.0042019 | 0.151955 |
| --- | --- | --- | 4.32901 | 3.66408 | 0.66493 | 1.58549 | 0.0082192 | 0.169323 |
| PCSK7 | chr11q23-q24 | proprotein convertase subtilisin/kexin  type 7 | 6.97811 | 6.54772 | 0.430388 | 1.3476 | 0.0086791 | 0.170916 |
| CIRBP | chr19p13.3 | cold inducible RNA binding protein | 7.19153 | 6.74233 | 0.449196 | 1.36528 | 0.0089802 | 0.172413 |
| CD6 | chr11q13 | CD6 molecule | 6.64551 | 6.17607 | 0.469434 | 1.38457 | 0.0047738 | 0.155828 |
| SRSF3 | chr6p21 | serine/arginine-rich splicing factor 3 | 5.71193 | 5.24111 | 0.470822 | 1.3859 | 0.0036034 | 0.150674 |
| --- | --- | --- | 4.68892 | 4.28326 | 0.405665 | 1.3247 | 0.0112929 | 0.182785 |
| EHBP1L1 | chr11q13.1 | EH domain binding protein 1 like 1 | 6.30743 | 5.8377 | 0.46973 | 1.38485 | 0.0092297 | 0.172989 |
| MGEA5 | chr10q24.1-  q24.3 | meningioma expressed antigen 5  (hyaluronidase) | 6.01191 | 5.61162 | 0.400291 | 1.31977 | 0.0060962 | 0.161446 |
| --- | --- | --- | 6.30565 | 5.89593 | 0.409718 | 1.32843 | 0.0091979 | 0.172989 |
| CRYZL1 | chr21q21.3 | crystallin zeta like 1 | 6.77153 | 6.33398 | 0.437551 | 1.3543 | 0.0065722 | 0.163699 |
| --- | --- | --- | 6.48204 | 6.02621 | 0.455827 | 1.37157 | 0.0020106 | 0.147268 |
| FDFT1 | chr8p23.1-p22 | farnesyl-diphosphate  farnesyltransferase 1 | 6.15131 | 5.72334 | 0.427973 | 1.34534 | 0.0053061 | 0.157883 |
| --- | --- | --- | 5.55578 | 5.10813 | 0.44765 | 1.36382 | 0.0058399 | 0.160158 |
| REPS1 | chr6q24.1 | RALBP1 associated Eps domain  containing 1 | 5.52129 | 5.11819 | 0.403104 | 1.32235 | 0.0112551 | 0.182674 |

| GTF2IRD2  /// GTF2IRD2B | chr7q11.23 | GTF2I repeat domain containing 2 /// GTF2I repeat domain containing 2B | 5.73454 | 5.29464 | 0.439904 | 1.35651 | 0.0061511 | 0.161641 |
| --- | --- | --- | --- | --- | --- | --- | --- | --- |
| --- | --- | --- | 6.86817 | 6.4346 | 0.433574 | 1.35058 | 0.0051874 | 0.157506 |
| GOLGA4 | chr3p22-p21.3 | golgin A4 | 7.193 | 6.80039 | 0.392614 | 1.31277 | 0.0040965 | 0.151685 |
| --- | --- | --- | 7.57218 | 7.11211 | 0.460062 | 1.3756 | 0.0016993 | 0.145076 |
| --- | --- | --- | 5.46338 | 5.02203 | 0.441352 | 1.35788 | 0.0041788 | 0.151901 |
| --- | --- | --- | 7.30675 | 6.81522 | 0.491522 | 1.40593 | 0.0032438 | 0.149592 |
| PLXND1 | chr3q22.1 | plexin D1 | 6.38815 | 5.99296 | 0.395191 | 1.31512 | 0.0134812 | 0.189772 |
| LINC01000 | chr7q32.1 | long intergenic non-protein coding  RNA 1000 | 7.86243 | 7.44445 | 0.417978 | 1.33605 | 0.0102156 | 0.177725 |
| --- | --- | --- | 6.18905 | 5.69647 | 0.49258 | 1.40696 | 0.0016749 | 0.145076 |
| MIAT | chr22q12.1 | myocardial infarction associated  transcript (non-protein coding) | 8.37197 | 7.90857 | 0.463405 | 1.37879 | 0.0176863 | 0.198559 |
| C2orf68 | chr2p11.2 | chromosome 2 open reading frame 68 | 7.02067 | 6.5734 | 0.447276 | 1.36346 | 0.0116684 | 0.185171 |
| --- | --- | --- | 8.85411 | 8.37524 | 0.478875 | 1.39366 | 0.0065696 | 0.163699 |
| ZFP90 | chr16q22.1 | ZFP90 zinc finger protein | 6.26059 | 5.84198 | 0.418607 | 1.33664 | 0.0171307 | 0.198289 |
| --- | --- | --- | 5.80997 | 5.31114 | 0.498832 | 1.41307 | 0.0013291 | 0.137103 |
| --- | --- | --- | 3.96605 | 3.61303 | 0.353014 | 1.27723 | 0.0125892 | 0.187104 |
| DNASE1 | chr16p13.3 | deoxyribonuclease I | 5.82139 | 5.45081 | 0.370585 | 1.29288 | 0.0095468 | 0.173132 |
| --- | --- | --- | 4.41208 | 3.98734 | 0.424749 | 1.34234 | 0.0047213 | 0.155506 |
| SLC38A10 | chr17q25.3 | solute carrier family 38, member 10 | 4.04766 | 3.60156 | 0.446104 | 1.36236 | 0.0027178 | 0.148407 |
| AKT3 | chr1q44 | v-akt murine thymoma viral oncogene  homolog 3 | 5.4615 | 5.12194 | 0.339552 | 1.26536 | 0.0140834 | 0.190686 |
| --- | --- | --- | 7.33057 | 6.97429 | 0.356282 | 1.28012 | 0.009118 | 0.172989 |
| --- | --- | --- | 7.30015 | 6.93194 | 0.368205 | 1.29075 | 0.0176787 | 0.198559 |
| --- | --- | --- | 4.65955 | 4.30491 | 0.354638 | 1.27867 | 0.0150243 | 0.193368 |
| C6orf89 | chr6p21.2 | chromosome 6 open reading frame 89 | 7.5388 | 7.17291 | 0.36589 | 1.28868 | 0.011668 | 0.185171 |
| EML3 | chr11q12.3 | echinoderm microtubule associated  protein like 3 | 6.33997 | 6.02723 | 0.312735 | 1.24206 | 0.0165869 | 0.196705 |
| PTCD3 | chr2p11.2 | pentatricopeptide repeat domain 3 | 7.32792 | 6.94635 | 0.381577 | 1.30277 | 0.0079903 | 0.168566 |
| FYN | chr6q21 | FYN proto-oncogene, Src family  tyrosine kinase | 8.62545 | 8.26507 | 0.360377 | 1.28376 | 0.0077876 | 0.168472 |
| TBRG1 | chr11q24.2 | transforming growth factor beta  regulator 1 | 5.31063 | 5.02108 | 0.289545 | 1.22225 | 0.0072676 | 0.167291 |
| TNRC6B | chr22q13.1 | trinucleotide repeat containing 6B | 8.28541 | 8.00438 | 0.281031 | 1.21506 | 0.0121994 | 0.186495 |
| HNRNPA3 | chr2q31.2 | heterogeneous nuclear  ribonucleoprotein A3 | 9.43839 | 9.13079 | 0.307604 | 1.23765 | 0.0077571 | 0.168472 |
| HSPB11 | chr1p32 | heat shock protein family B (small),  member 11 | 5.24176 | 4.91279 | 0.32897 | 1.25612 | 0.0047165 | 0.155506 |
| --- | --- | --- | 5.86016 | 5.52493 | 0.335225 | 1.26157 | 0.0181036 | 0.198976 |
| ZBTB20 | chr3q13.2 | zinc finger and BTB domain containing  20 | 8.55772 | 8.25052 | 0.307206 | 1.23731 | 0.0103438 | 0.178293 |
| ZBTB20 | chr3q13.2 | zinc finger and BTB domain containing  20 | 7.42571 | 7.04044 | 0.38528 | 1.30611 | 0.0039959 | 0.151256 |
| --- | --- | --- | 5.87771 | 5.55927 | 0.318444 | 1.24698 | 0.0174064 | 0.198524 |
| --- | --- | --- | 5.64104 | 5.34496 | 0.296082 | 1.22781 | 0.0178902 | 0.198662 |
| ZNF274 | chr19qter | zinc finger protein 274 | 6.61146 | 6.31021 | 0.301247 | 1.23221 | 0.0128295 | 0.187472 |
| --- | --- | --- | 6.0625 | 5.69721 | 0.365283 | 1.28813 | 0.0071127 | 0.166748 |
| PLXND1 | chr3q22.1 | plexin D1 | 7.1834 | 6.86234 | 0.321065 | 1.24925 | 0.0082707 | 0.169323 |
| --- | --- | --- | 6.15156 | 5.76559 | 0.385972 | 1.30674 | 0.0045768 | 0.155277 |

| NME6 | chr3p21 | NME/NM23 nucleoside diphosphate  kinase 6 | 5.88454 | 5.53838 | 0.34616 | 1.27117 | 0.0084224 | 0.170281 |
| --- | --- | --- | --- | --- | --- | --- | --- | --- |
| LRCH3 | chr3q29 | leucine-rich repeats and calponin homology (CH) domain containing 3 | 6.52232 | 6.19539 | 0.326927 | 1.25434 | 0.0139834 | 0.190686 |
| ANKH | chr5p15.1 | ANKH inorganic pyrophosphate  transport regulator | 6.66381 | 6.30236 | 0.361453 | 1.28472 | 0.0059657 | 0.160924 |
| FBXO9 | chr6p12.3-p11.2 | F-box protein 9 | 5.88573 | 5.50819 | 0.377535 | 1.29912 | 0.0143325 | 0.191711 |
| CEP68 | chr2p14 | centrosomal protein 68kDa | 6.76691 | 6.38886 | 0.378047 | 1.29958 | 0.0050147 | 0.157506 |
| BCL11B | chr14q32.2 | B-cell CLL/lymphoma 11B (zinc finger  protein) | 9.98421 | 9.61457 | 0.369642 | 1.29203 | 0.0087116 | 0.17099 |
| CTC1 | chr17p13.1 | CTS telomere maintenance complex  component 1 | 5.96511 | 5.59432 | 0.370788 | 1.29306 | 0.0171481 | 0.198289 |
| THEM4 | chr1q21 | thioesterase superfamily member 4 | 6.76176 | 6.37333 | 0.388426 | 1.30896 | 0.0131993 | 0.189124 |
| KIAA1671 | chr22q11.23 | KIAA1671 | 6.73202 | 6.31517 | 0.416851 | 1.33501 | 0.0125263 | 0.187104 |
| --- | --- | --- | 5.71067 | 5.35533 | 0.35534 | 1.27929 | 0.0155331 | 0.194293 |
| TRAF3IP3 | chr1q32 | TRAF3 interacting protein 3 | 6.36421 | 5.96112 | 0.403082 | 1.32233 | 0.0139058 | 0.190686 |
| --- | --- | --- | 5.34244 | 5.01065 | 0.331784 | 1.25857 | 0.0133691 | 0.189772 |
| SFXN1 | chr5q35.3 | sideroflexin 1 | 6.82232 | 6.46746 | 0.35486 | 1.27886 | 0.0134435 | 0.189772 |
| UBE2I | chr16p13.3 | ubiquitin conjugating enzyme E2I | 6.52902 | 6.23988 | 0.28914 | 1.22191 | 0.0106444 | 0.180552 |
| ANKH | chr5p15.1 | ANKH inorganic pyrophosphate  transport regulator | 9.24671 | 8.95158 | 0.295134 | 1.227 | 0.0124193 | 0.187004 |
| LOC101928  673 | chr1q25.1 | uncharacterized LOC101928673 | 5.31495 | 5.01061 | 0.304344 | 1.23486 | 0.0103903 | 0.17876 |
| MIAT | chr22q12.1 | myocardial infarction associated  transcript (non-protein coding) | 6.07414 | 5.70095 | 0.37319 | 1.29521 | 0.0150374 | 0.193368 |
| --- | --- | --- | 5.28627 | 4.97285 | 0.313428 | 1.24266 | 0.0149227 | 0.192633 |
| RUNX1-IT1 | chr21q22.12 | RUNX1 intronic transcript 1 | 7.11794 | 6.76471 | 0.353231 | 1.27742 | 0.0120508 | 0.186203 |
| NLRC3 | chr16p13.3 | NLR family, CARD domain containing 3 | 9.19292 | 8.83688 | 0.35604 | 1.27991 | 0.0169738 | 0.197956 |
| GLCCI1 | chr7p21.3 | glucocorticoid induced 1 | 8.70222 | 8.28293 | 0.419284 | 1.33726 | 0.0076636 | 0.168472 |
| ATP2A3 | chr17p13.3 | ATPase, Ca++ transporting, ubiquitous | 4.18415 | 3.87036 | 0.313784 | 1.24296 | 0.010482 | 0.179106 |
| GTF3C2 | chr2p23.3 | general transcription factor IIIC subunit  2 | 5.43675 | 5.11011 | 0.326642 | 1.25409 | 0.0128817 | 0.187472 |
| MAVS | chr20p13 | mitochondrial antiviral signaling  protein | 7.29828 | 6.99471 | 0.30357 | 1.23419 | 0.0127797 | 0.187417 |
| AAK1 | chr2p14 | AP2 associated kinase 1 | 9.35269 | 9.02008 | 0.332606 | 1.25929 | 0.0136755 | 0.190437 |
| EML3 | chr11q12.3 | echinoderm microtubule associated  protein like 3 | 5.97032 | 5.62309 | 0.347237 | 1.27212 | 0.0024169 | 0.148407 |
| --- | --- | --- | 5.00365 | 4.64494 | 0.358707 | 1.28228 | 0.0108033 | 0.181402 |
| --- | --- | --- | 3.22933 | 2.92521 | 0.304115 | 1.23466 | 0.0125293 | 0.187104 |
| ORMDL1 | chr2q32 | ORMDL sphingolipid biosynthesis  regulator 1 | 7.83263 | 7.51707 | 0.315553 | 1.24449 | 0.0077926 | 0.168472 |
| --- | --- | --- | 5.64896 | 5.27603 | 0.372929 | 1.29498 | 0.0108224 | 0.181472 |
| --- | --- | --- | 6.66404 | 6.30146 | 0.362585 | 1.28573 | 0.0063921 | 0.163699 |
| --- | --- | --- | 6.70686 | 6.3149 | 0.391962 | 1.31218 | 0.0067186 | 0.164139 |
| --- | --- | --- | 5.93136 | 5.59273 | 0.338632 | 1.26456 | 0.0124841 | 0.187104 |
| TMEM245 | chr9q31 | transmembrane protein 245 | 7.20297 | 6.80292 | 0.400042 | 1.31955 | 0.008011 | 0.168575 |
| MUM1 | chr19p13.3 | melanoma associated antigen  (mutated) 1 | 5.64752 | 5.32753 | 0.319992 | 1.24832 | 0.0097663 | 0.17394 |
| ZNF397 | chr18q12.2 | zinc finger protein 397 | 6.59923 | 6.27525 | 0.32398 | 1.25178 | 0.0045484 | 0.154948 |
| --- | --- | --- | 3.58687 | 3.28663 | 0.300245 | 1.23135 | 0.0128322 | 0.187472 |

| --- | --- | --- | 5.57925 | 5.25323 | 0.326018 | 1.25355 | 0.0104615 | 0.179052 |
| --- | --- | --- | --- | --- | --- | --- | --- | --- |
| THUMPD3-  AS1 | chr3p25.3 | THUMPD3 antisense RNA 1 | 6.62452 | 6.29904 | 0.325487 | 1.25309 | 0.0033866 | 0.150538 |
| THUMPD3-  AS1 | chr3p25.3 | THUMPD3 antisense RNA 1 | 6.84278 | 6.50173 | 0.341047 | 1.26668 | 0.0041266 | 0.151685 |
| SYNJ2BP | chr14q24.2 | synaptojanin 2 binding protein | 4.5622 | 4.22571 | 0.336491 | 1.26268 | 0.0053665 | 0.158323 |
| SFSWAP | chr12q24.33 | splicing factor, suppressor of white-  apricot family | 4.45768 | 4.08946 | 0.368216 | 1.29076 | 0.0059041 | 0.160245 |
| --- | --- | --- | 5.46081 | 5.1085 | 0.352312 | 1.27661 | 0.0094804 | 0.173123 |
| FAN1 | chr15q13.2-  q13.3 | FANCD2/FANCI-associated nuclease 1 | 4.63635 | 4.26002 | 0.376326 | 1.29803 | 0.0020311 | 0.147757 |
| MYL12A | chr18p11.31 | myosin light chain 12A | 3.86833 | 3.54278 | 0.325544 | 1.25314 | 0.0046096 | 0.155277 |
| CMTM7 | chr3p22.3 | CKLF-like MARVEL transmembrane  domain containing 7 | 6.60994 | 6.27873 | 0.331216 | 1.25807 | 0.0103183 | 0.178164 |
| ATP9B | chr18q23 | ATPase, class II, type 9B | 5.17534 | 4.82587 | 0.349476 | 1.2741 | 0.0006877 | 0.128628 |
| --- | --- | --- | 4.83144 | 4.45321 | 0.378227 | 1.29974 | 0.0017912 | 0.145076 |
| --- | --- | --- | 6.24046 | 5.83005 | 0.410404 | 1.32906 | 0.0082306 | 0.169323 |
| --- | --- | --- | 5.42598 | 5.12388 | 0.3021 | 1.23294 | 0.0180516 | 0.198864 |
| TRAPPC10 | chr21q22.3 | trafficking protein particle complex 10 | 8.11712 | 7.72913 | 0.387993 | 1.30857 | 0.0065859 | 0.163766 |
| --- | --- | --- | 5.24681 | 4.92544 | 0.32137 | 1.24952 | 0.0175979 | 0.198559 |
| POLR2J4 | chr7p13 | polymerase (RNA) II (DNA directed)  polypeptide J4, pseudogene | 7.42397 | 7.10032 | 0.323648 | 1.25149 | 0.0156332 | 0.194408 |
| --- | --- | --- | 4.81596 | 4.42657 | 0.389391 | 1.30984 | 0.0122837 | 0.186743 |
| ITFG2 ///  LOC100507 424 | chr12p13.33 | integrin alpha FG-GAP repeat  containing 2 /// uncharacterized LOC100507424 | 5.34122 | 4.99217 | 0.349047 | 1.27372 | 0.0089546 | 0.172413 |
| NFATC2IP | chr16p11.2 | nuclear factor of activated T-cells,  cytoplasmic, calcineurin-dependent 2  interacting protein | 6.9213 | 6.49868 | 0.422622 | 1.34036 | 0.0057021 | 0.158791 |
| URGCP | chr7p13 | upregulator of cell proliferation | 6.19409 | 5.90231 | 0.291781 | 1.22415 | 0.0177452 | 0.198662 |
| GLYR1 | chr16p13.3 | glyoxylate reductase 1 homolog  (Arabidopsis) | 5.42965 | 5.03456 | 0.39509 | 1.31502 | 0.0019574 | 0.146411 |
| --- | --- | --- | 5.53294 | 5.24253 | 0.290407 | 1.22299 | 0.0168878 | 0.197956 |
| FBXO9 | chr6p12.3-p11.2 | F-box protein 9 | 6.07765 | 5.729 | 0.34865 | 1.27337 | 0.0114511 | 0.183744 |
| --- | --- | --- | 5.66431 | 5.24032 | 0.423993 | 1.34164 | 0.0052667 | 0.157676 |
| YBEY | chr21q22.3 | ybeY metallopeptidase (putative) | 5.62832 | 5.27874 | 0.349589 | 1.2742 | 0.0160425 | 0.195587 |
| --- | --- | --- | 6.72522 | 6.44199 | 0.283233 | 1.21692 | 0.0176281 | 0.198559 |
| FAM160B2 | chr8p21.3 | family with sequence similarity 160,  member B2 | 7.10694 | 6.80249 | 0.304452 | 1.23495 | 0.0052212 | 0.157641 |
| UBE2G2 | chr21q22.3 | ubiquitin conjugating enzyme E2G 2 | 8.00765 | 7.72249 | 0.28516 | 1.21855 | 0.015928 | 0.195334 |
| KIAA1919 | chr6q22 | KIAA1919 | 5.10842 | 4.75719 | 0.351225 | 1.27564 | 0.0092503 | 0.172989 |
| CEP68 | chr2p14 | centrosomal protein 68kDa | 5.66195 | 5.32134 | 0.34061 | 1.26629 | 0.01084 | 0.181472 |
| ZKSCAN1 | chr7q22 | zinc finger with KRAB and SCAN  domains 1 | 7.18616 | 6.80346 | 0.3827 | 1.30378 | 0.0096568 | 0.173413 |
| --- | --- | --- | 4.66937 | 4.26972 | 0.399651 | 1.31919 | 0.011715 | 0.185453 |
| SPG7 | chr16q24.3 | spastic paraplegia 7 (pure and  complicated autosomal recessive) | 7.14505 | 6.77612 | 0.368928 | 1.29139 | 0.0110985 | 0.182206 |
| --- | --- | --- | 6.94854 | 6.58005 | 0.368496 | 1.29101 | 0.017148 | 0.198289 |
| NFATC2IP | chr16p11.2 | nuclear factor of activated T-cells,  cytoplasmic, calcineurin-dependent 2 interacting protein | 8.0684 | 7.69378 | 0.374615 | 1.29649 | 0.0110909 | 0.182158 |
| EVL | chr14q32.2 | Enah/Vasp-like | 8.34036 | 7.98701 | 0.353345 | 1.27752 | 0.0147946 | 0.192079 |
| --- | --- | --- | 7.39078 | 7.02473 | 0.366052 | 1.28882 | 0.0081764 | 0.169323 |

| CBFA2T2 | chr20q11 | core-binding factor, runt domain,  alpha subunit 2; translocated to, 2 | 7.71628 | 7.38403 | 0.332249 | 1.25897 | 0.0127842 | 0.187417 |
| --- | --- | --- | --- | --- | --- | --- | --- | --- |
| --- | --- | --- | 6.67117 | 6.29878 | 0.372388 | 1.29449 | 0.0077486 | 0.168472 |
| --- | --- | --- | 7.87933 | 7.5851 | 0.294234 | 1.22623 | 0.0151506 | 0.193396 |
| --- | --- | --- | 7.42239 | 7.06469 | 0.357701 | 1.28138 | 0.0060274 | 0.161186 |
| --- | --- | --- | 8.14118 | 7.74245 | 0.398729 | 1.31835 | 0.0072897 | 0.167406 |
| --- | --- | --- | 6.07626 | 5.69532 | 0.380939 | 1.30219 | 0.0120454 | 0.186203 |
| --- | --- | --- | 6.19284 | 5.82702 | 0.365827 | 1.28862 | 0.0051781 | 0.157506 |
| SUGP2 | chr19p12 | SURP and G-patch domain containing 2 | 5.78174 | 5.41413 | 0.36761 | 1.29021 | 0.0091433 | 0.172989 |
| ZKSCAN1 | chr7q22 | zinc finger with KRAB and SCAN  domains 1 | 7.00944 | 6.58912 | 0.420314 | 1.33822 | 0.0026928 | 0.148407 |
| SGSM2 | chr17p13.3 | small G protein signaling modulator 2 | 5.47503 | 5.06242 | 0.412606 | 1.33109 | 0.0144944 | 0.191711 |
| FAM118A | chr22q13 | family with sequence similarity 118,  member A | 8.21542 | 7.77241 | 0.44301 | 1.35944 | 0.0058187 | 0.160158 |
| --- | --- | --- | 6.05513 | 5.54859 | 0.506536 | 1.42064 | 0.0016806 | 0.145076 |
| SLC25A37 | chr8p21.2 | solute carrier family 25 (mitochondrial iron transporter), member 37 | 4.63682 | 4.17521 | 0.461606 | 1.37707 | 0.0052493 | 0.157641 |
| RAB11FIP4 | chr17q11.2 | RAB11 family interacting protein 4  (class II) | 4.00562 | 3.44134 | 0.564276 | 1.47865 | 0.0052815 | 0.157883 |
| ANAPC5 | chr12q24.31 | anaphase promoting complex subunit  5 | 5.82578 | 5.22757 | 0.59821 | 1.51384 | 0.0092125 | 0.172989 |
| SMURF2 | chr17q22-q23 | SMAD specific E3 ubiquitin protein  ligase 2 | 5.45304 | 4.84069 | 0.612351 | 1.52875 | 0.00413 | 0.151685 |
| --- | --- | --- | 6.41576 | 5.78774 | 0.628013 | 1.54544 | 0.0083026 | 0.169437 |
| RUSC1-AS1 | chr1q22 | RUSC1 antisense RNA 1 | 7.16558 | 6.70321 | 0.462363 | 1.3778 | 0.0168049 | 0.197728 |
| --- | --- | --- | 7.55062 | 7.07472 | 0.475892 | 1.39078 | 0.0086106 | 0.170682 |
| WSB1 | chr17q11.1 | WD repeat and SOCS box containing 1 | 6.32979 | 5.77488 | 0.554908 | 1.46908 | 0.0050954 | 0.157506 |
| PSMA3-AS1 | chr14q23.1 | PSMA3 antisense RNA 1 | 6.93661 | 6.37775 | 0.558856 | 1.4731 | 0.0099241 | 0.174971 |
| --- | --- | --- | 8.89795 | 8.403 | 0.494955 | 1.40928 | 0.0079446 | 0.168472 |
| PPP1R16B | chr20q11.23 | protein phosphatase 1, regulatory  subunit 16B | 7.8743 | 7.28632 | 0.587981 | 1.50314 | 0.0046192 | 0.155277 |
| SRSF4 | chr1p35.3 | serine/arginine-rich splicing factor 4 | 6.04794 | 5.54753 | 0.500412 | 1.41462 | 0.0077115 | 0.168472 |
| EML4 | chr2p21 | echinoderm microtubule associated  protein like 4 | 6.57275 | 5.93443 | 0.638315 | 1.55651 | 0.0010199 | 0.130044 |
| NEAT1 | chr11q13.1 | nuclear paraspeckle assembly  transcript 1 (non-protein coding) | 5.66395 | 5.20905 | 0.454897 | 1.37068 | 0.0113484 | 0.183076 |
| --- | --- | --- | 5.51141 | 4.97188 | 0.539533 | 1.4535 | 0.014065 | 0.190686 |
| --- | --- | --- | 4.92103 | 4.41593 | 0.505106 | 1.41923 | 0.0108107 | 0.181402 |
| --- | --- | --- | 8.44654 | 7.9362 | 0.510345 | 1.42439 | 0.017079 | 0.198257 |
| DLEU2 | chr13q14.3 | deleted in lymphocytic leukemia 2  (non-protein coding) | 7.16309 | 6.62063 | 0.542465 | 1.45646 | 0.0082299 | 0.169323 |
| C5orf28 | chr5p12 | chromosome 5 open reading frame 28 | 6.75975 | 6.18682 | 0.572934 | 1.48755 | 0.0164866 | 0.196619 |
| PSMA3-AS1 | chr14q23.1 | PSMA3 antisense RNA 1 | 6.80501 | 6.31777 | 0.487239 | 1.40176 | 0.0178981 | 0.198662 |
| TCP11L2 | chr12q23.3 | t-complex 11, testis-specific-like 2 | 7.21943 | 6.713 | 0.506429 | 1.42053 | 0.0088886 | 0.172413 |
| TAF15 | chr17q11.1-  q11.2 | TATA box binding protein associated  factor 15 | 7.71241 | 7.11688 | 0.595537 | 1.51103 | 0.0092478 | 0.172989 |
| CTAGE5 | chr14q13.3 | CTAGE family, member 5 | 6.54759 | 6.13728 | 0.410307 | 1.32897 | 0.015144 | 0.193396 |
| --- | --- | --- | 7.77695 | 7.3025 | 0.474451 | 1.38939 | 0.0106711 | 0.180552 |

| SMARCC2 | chr12q13.2 | SWI/SNF related, matrix associated, actin dependent regulator of chromatin, subfamily c, member 2 | 5.39172 | 4.90682 | 0.484896 | 1.39948 | 0.0089535 | 0.172413 |
| --- | --- | --- | --- | --- | --- | --- | --- | --- |
| --- | --- | --- | 5.94783 | 5.38103 | 0.566799 | 1.48123 | 0.0124023 | 0.187004 |
| --- | --- | --- | 8.29023 | 7.69008 | 0.600155 | 1.51588 | 0.0136538 | 0.190437 |
| KIAA0754  /// MACF1 | chr1p32-p31 ///  chr1p34.3 | KIAA0754 /// microtubule-actin  crosslinking factor 1 | 5.1879 | 4.64929 | 0.538618 | 1.45258 | 0.0180007 | 0.198662 |
| --- | --- | --- | 5.54298 | 4.84017 | 0.702809 | 1.62767 | 0.0018085 | 0.145277 |
| LZTFL1 | chr3p21.3 | leucine zipper transcription factor like  1 | 3.71168 | 3.28814 | 0.423543 | 1.34122 | 0.0161147 | 0.195587 |
| CXorf56 | chrXq23 | chromosome X open reading frame 56 | 3.82711 | 3.31349 | 0.513622 | 1.42763 | 0.0143497 | 0.191711 |
| AKAP5 | chr14q23.3 | A kinase (PRKA) anchor protein 5 | 4.55291 | 3.98633 | 0.566588 | 1.48102 | 0.0048593 | 0.156541 |
| --- | --- | --- | 3.94518 | 3.56041 | 0.384771 | 1.30565 | 0.0136761 | 0.190437 |
| --- | --- | --- | 4.59658 | 4.18362 | 0.412953 | 1.33141 | 0.0140454 | 0.190686 |
| --- | --- | --- | 6.42398 | 5.99489 | 0.429083 | 1.34638 | 0.0096996 | 0.173861 |
| --- | --- | --- | 4.58101 | 4.11482 | 0.466189 | 1.38146 | 0.0140234 | 0.190686 |
| TP53BP1 | chr15q15-q21 | tumor protein p53 binding protein 1 | 5.24706 | 4.93005 | 0.317007 | 1.24574 | 0.0107075 | 0.180804 |
| CAPRIN1 | chr11p13 | cell cycle associated protein 1 | 3.6663 | 3.36917 | 0.297128 | 1.2287 | 0.0096065 | 0.173173 |
| LRRFIP1 | chr2q37.3 | leucine rich repeat (in FLII) interacting  protein 1 | 5.13423 | 4.80741 | 0.326823 | 1.25425 | 0.0061298 | 0.161558 |
| SRGN | chr10q22.1 | serglycin | 6.72144 | 6.38839 | 0.333046 | 1.25967 | 0.0182178 | 0.199412 |
| LOC105379  752 | --- | uncharacterized LOC105379752 | 5.74487 | 5.33619 | 0.408673 | 1.32746 | 0.0061879 | 0.161803 |
| LOC101060  521 ///  POLR3E | chr16p12.2 | DNA-directed RNA polymerase III subunit RPC5 /// polymerase (RNA) III (DNA directed) polypeptide E (80kD) | 4.89449 | 4.47989 | 0.414595 | 1.33292 | 0.0053377 | 0.158115 |
| PCNX | chr14q24.2 | pecanex homolog (Drosophila) | 6.1623 | 5.7122 | 0.450104 | 1.36614 | 0.007223 | 0.167222 |
| GOSR1 | chr17q11 | golgi SNAP receptor complex member  1 | 3.72625 | 3.24495 | 0.481301 | 1.396 | 0.0047212 | 0.155506 |
| RNF125 | chr18q12.1 | ring finger protein 125, E3 ubiquitin  protein ligase | 6.71996 | 6.15228 | 0.567679 | 1.48214 | 0.0020449 | 0.147757 |
| VWA9 | chr15q22.31 | von Willebrand factor A domain  containing 9 | 4.58134 | 4.13867 | 0.442664 | 1.35911 | 0.0144428 | 0.191711 |
| --- | --- | --- | 5.52722 | 5.02005 | 0.507166 | 1.42126 | 0.0063332 | 0.163056 |
| GNL3L | chrXp11.22 | guanine nucleotide binding protein-  like 3 (nucleolar)-like | 6.88174 | 6.2977 | 0.584047 | 1.49905 | 0.0018149 | 0.145491 |
| --- | --- | --- | 4.92711 | 4.42443 | 0.502677 | 1.41684 | 0.0014254 | 0.139818 |
| FAM49B | chr8q24.21 | family with sequence similarity 49,  member B | 6.06951 | 5.54548 | 0.524027 | 1.43796 | 0.0012662 | 0.136585 |
| SNRPN | chr15q11.2 | small nuclear ribonucleoprotein  polypeptide N | 5.18843 | 4.60384 | 0.584592 | 1.49961 | 0.0002184 | 0.111326 |
| VPS8 | chr3q27.2 | vacuolar protein sorting 8 homolog (S.  cerevisiae) | 4.36551 | 3.75069 | 0.614814 | 1.53136 | 0.0017896 | 0.145076 |
| VEZT | chr12q22 | vezatin, adherens junctions  transmembrane protein | 3.15064 | 2.86228 | 0.28836 | 1.22125 | 0.0009962 | 0.129126 |
| TRIO | chr5p15.2 | trio Rho guanine nucleotide exchange  factor | 3.34376 | 3.02236 | 0.321402 | 1.24954 | 3.63E-05 | 0.103347 |
| ZMIZ1 | chr10q22.3 | zinc finger, MIZ-type containing 1 | 4.78914 | 4.46138 | 0.327753 | 1.25506 | 0.0010673 | 0.13333 |
| --- | --- | --- | 2.93663 | 2.58911 | 0.347517 | 1.27237 | 0.0084994 | 0.170624 |
| MAP3K2 | chr2q14.3 | mitogen-activated protein kinase  kinase kinase 2 | 4.04683 | 3.67831 | 0.368522 | 1.29103 | 0.0023324 | 0.148407 |
| ARHGAP26 | chr5q31 | Rho GTPase activating protein 26 | 2.62719 | 2.23091 | 0.396288 | 1.31612 | 0.0079174 | 0.168472 |

| --- | --- | --- | 3.7306 | 3.31668 | 0.413922 | 1.3323 | 0.004575 | 0.155277 |
| --- | --- | --- | --- | --- | --- | --- | --- | --- |
| --- | --- | --- | 5.8939 | 5.50029 | 0.393603 | 1.31367 | 0.006078 | 0.161206 |
| --- | --- | --- | 5.74985 | 5.3491 | 0.400744 | 1.32019 | 0.0083601 | 0.169992 |
| --- | --- | --- | 3.35278 | 3.00168 | 0.351093 | 1.27553 | 0.0092184 | 0.172989 |
| RAB28 | chr4p15.33 | RAB28, member RAS oncogene family | 3.47706 | 3.11542 | 0.361639 | 1.28488 | 0.0041118 | 0.151685 |
| --- | --- | --- | 4.07692 | 3.63356 | 0.443365 | 1.35977 | 0.0058706 | 0.160245 |
| RNFT1 | chr17q23.1 | ring finger protein, transmembrane 1 | 4.19365 | 3.85022 | 0.343425 | 1.26877 | 0.011447 | 0.183744 |
| --- | --- | --- | 4.04344 | 3.63709 | 0.406349 | 1.32533 | 0.0153151 | 0.193632 |
| --- | --- | --- | 4.62539 | 4.19042 | 0.434972 | 1.35188 | 0.0030432 | 0.148407 |
| --- | --- | --- | 3.48778 | 2.95793 | 0.529847 | 1.44378 | 0.0044386 | 0.153634 |
| --- | --- | --- | 5.21884 | 4.75903 | 0.459808 | 1.37536 | 0.0118676 | 0.185824 |
| --- | --- | --- | 3.82894 | 3.22034 | 0.608597 | 1.52478 | 0.0026058 | 0.148407 |
| --- | --- | --- | 4.6445 | 4.02628 | 0.618222 | 1.53498 | 0.0025853 | 0.148407 |
| --- | --- | --- | 3.53854 | 2.89545 | 0.643092 | 1.56167 | 0.0004172 | 0.112336 |
| GNL3L | chrXp11.22 | guanine nucleotide binding protein-  like 3 (nucleolar)-like | 4.44319 | 3.93409 | 0.509102 | 1.42316 | 0.0070187 | 0.166168 |
| ATM | chr11q22-q23 | ATM serine/threonine kinase | 8.21157 | 7.68497 | 0.526592 | 1.44052 | 0.0042153 | 0.151955 |
| ZNF652 | chr17q21.32 | zinc finger protein 652 | 6.50079 | 5.86221 | 0.638586 | 1.5568 | 0.0006815 | 0.128628 |
| --- | --- | --- | 4.44787 | 3.84035 | 0.607521 | 1.52364 | 0.0133445 | 0.189772 |
| TEP1 | chr14q11.2 | telomerase-associated protein 1 | 5.21148 | 4.62154 | 0.589932 | 1.50518 | 0.0042019 | 0.151955 |
| LRRC37A2  /// LRRC37A3 | chr17q21.31 /// chr17q24.1 | leucine rich repeat containing 37,  member A2 /// leucine rich repeat containing 37, member A3 | 5.35228 | 4.76801 | 0.58428 | 1.49929 | 0.0026592 | 0.148407 |
| PDP2 | chr16q22.1 | pyruvate dehyrogenase phosphatase  catalytic subunit 2 | 4.36711 | 3.70438 | 0.662732 | 1.58308 | 0.0018653 | 0.14618 |
| CRTC3 | chr15q26.1 | CREB regulated transcription  coactivator 3 | 4.78962 | 4.30555 | 0.484076 | 1.39869 | 0.0134715 | 0.189772 |
| MEF2A | chr15q26 | myocyte enhancer factor 2A | 5.0193 | 4.41024 | 0.609057 | 1.52526 | 0.0013316 | 0.137103 |
| RPAIN | chr17p13.2 | RPA interacting protein | 4.88803 | 4.46054 | 0.427499 | 1.3449 | 0.0150818 | 0.193396 |
| POLR2J3 | chr7q22.1 | polymerase (RNA) II (DNA directed)  polypeptide J3 | 5.52995 | 5.09253 | 0.437417 | 1.35418 | 0.0110652 | 0.181975 |
| --- | --- | --- | 4.54706 | 4.05007 | 0.496987 | 1.41126 | 0.0092539 | 0.172989 |
| CDC14A | chr1p21 | cell division cycle 14A | 4.76808 | 4.19189 | 0.576192 | 1.49091 | 0.0012034 | 0.136585 |
| --- | --- | --- | 3.75024 | 3.34241 | 0.407832 | 1.32669 | 0.0110225 | 0.181825 |
| UBR2 | chr6p21.1 | ubiquitin protein ligase E3 component  n-recognin 2 | 5.29115 | 4.66756 | 0.623588 | 1.5407 | 0.0009992 | 0.129126 |
| --- | --- | --- | 4.508 | 3.93846 | 0.569541 | 1.48405 | 0.0030922 | 0.148407 |
| --- | --- | --- | 5.88071 | 5.17298 | 0.707737 | 1.63324 | 0.0017701 | 0.145076 |
| LGALS3 | chr14q22.3 | lectin, galactoside-binding, soluble, 3 | 5.54583 | 4.82783 | 0.718001 | 1.6449 | 0.0106675 | 0.180552 |
| --- | --- | --- | 4.85815 | 4.27894 | 0.579208 | 1.49403 | 0.0109616 | 0.181825 |
| --- | --- | --- | 4.70033 | 4.08931 | 0.611019 | 1.52734 | 0.0046162 | 0.155277 |
| --- | --- | --- | 5.11289 | 4.53782 | 0.575063 | 1.48974 | 0.0102478 | 0.177837 |
| --- | --- | --- | 5.54093 | 4.91249 | 0.628431 | 1.54588 | 0.0011811 | 0.134826 |
| --- | --- | --- | 4.28206 | 3.63736 | 0.644705 | 1.56342 | 0.0090935 | 0.172989 |
| --- | --- | --- | 6.09937 | 5.34953 | 0.749843 | 1.68161 | 0.0032914 | 0.149592 |
| GSDMB | chr17q12 | gasdermin B | 4.17275 | 3.64118 | 0.531571 | 1.4455 | 0.0125528 | 0.187104 |
| SNAPC3 | chr9p22.3 | small nuclear RNA activating complex  polypeptide 3 | 3.93174 | 3.38044 | 0.551305 | 1.46541 | 0.0055435 | 0.158791 |
| --- | --- | --- | 5.57971 | 5.01708 | 0.56263 | 1.47696 | 0.0043031 | 0.152719 |
| ACSL4 | chrXq22.3-q23 | acyl-CoA synthetase long-chain family  member 4 | 4.24943 | 3.63851 | 0.61092 | 1.52723 | 0.0056389 | 0.158791 |

| --- | --- | --- | 4.7977 | 4.17537 | 0.622323 | 1.53935 | 0.0044507 | 0.153634 |
| --- | --- | --- | --- | --- | --- | --- | --- | --- |
| --- | --- | --- | 4.60837 | 3.9424 | 0.665964 | 1.58663 | 0.0030124 | 0.148407 |
| RAB30-AS1 | --- | RAB30 antisense RNA 1 (head to head) | 3.67306 | 2.91996 | 0.753096 | 1.68541 | 0.0001729 | 0.111326 |
| COA1 | chr7p13 | cytochrome c oxidase assembly factor  1 homolog | 7.5691 | 7.02234 | 0.546758 | 1.4608 | 0.0173435 | 0.198289 |
| --- | --- | --- | 6.54045 | 6.16544 | 0.375006 | 1.29684 | 0.0136543 | 0.190437 |
| --- | --- | --- | 4.04821 | 3.56457 | 0.483636 | 1.39826 | 0.005587 | 0.158791 |
| --- | --- | --- | 4.81315 | 4.31231 | 0.500847 | 1.41504 | 0.0102889 | 0.177873 |
| --- | --- | --- | 4.84449 | 4.29161 | 0.552873 | 1.467 | 0.007581 | 0.168472 |
| SEPT9 | chr17q25 | septin 9 | 5.87418 | 5.33765 | 0.536529 | 1.45048 | 0.007789 | 0.168472 |
| TRIOBP | chr22q13.1 | TRIO and F-actin binding protein | 4.65022 | 4.09679 | 0.553437 | 1.46758 | 0.0006512 | 0.128628 |
| LOC100131  541 | chr11q21 | uncharacterized LOC100131541 | 6.97004 | 6.42429 | 0.545753 | 1.45978 | 0.0057661 | 0.159764 |
| SMAD4 | chr18q21.1 | SMAD family member 4 | 6.48516 | 5.89437 | 0.590787 | 1.50607 | 0.0017813 | 0.145076 |
| --- | --- | --- | 6.05936 | 5.38338 | 0.675972 | 1.59767 | 0.0077076 | 0.168472 |
| --- | --- | --- | 6.37353 | 5.60139 | 0.77214 | 1.7078 | 0.0066216 | 0.16393 |
| N4BP2L2 | chr13q13.1 | NEDD4 binding protein 2-like 2 | 5.79762 | 5.02868 | 0.768937 | 1.70401 | 0.0055601 | 0.158791 |
| --- | --- | --- | 4.97147 | 4.09852 | 0.872954 | 1.83141 | 0.0027333 | 0.148407 |
| SH2D1B | chr1q23.3 | SH2 domain containing 1B | 7.09552 | 6.44419 | 0.651326 | 1.57061 | 0.0156041 | 0.194408 |
| MDM4 | chr1q32 | MDM4, p53 regulator | 6.3475 | 5.6307 | 0.716797 | 1.64353 | 0.0117562 | 0.185589 |
| EIF2S3 | chrXp22.2-p22.1 | eukaryotic translation initiation factor  2, subunit 3 gamma, 52kDa | 8.47578 | 7.71918 | 0.756602 | 1.68951 | 0.013222 | 0.189342 |
| TBCD | chr17q25.3 | tubulin folding cofactor D | 7.5629 | 6.70437 | 0.85853 | 1.81319 | 0.0169618 | 0.197956 |
| GALT | chr9p13 | galactose-1-phosphate  uridylyltransferase | 4.81983 | 4.28254 | 0.53729 | 1.45124 | 0.016117 | 0.195587 |
| IKZF3 | chr17q21 | IKAROS family zinc finger 3 | 6.75619 | 6.14137 | 0.614824 | 1.53137 | 0.0097485 | 0.173861 |
| --- | --- | --- | 4.09109 | 3.60446 | 0.486629 | 1.40117 | 0.0180831 | 0.198866 |
| --- | --- | --- | 7.14403 | 6.48557 | 0.658463 | 1.5784 | 0.0031139 | 0.148407 |
| --- | --- | --- | 4.82104 | 4.34316 | 0.477874 | 1.39269 | 0.0077443 | 0.168472 |
| --- | --- | --- | 4.60743 | 4.0855 | 0.521921 | 1.43587 | 0.0086859 | 0.170916 |
| --- | --- | --- | 5.03128 | 4.51192 | 0.519361 | 1.43332 | 0.0176799 | 0.198559 |
| --- | --- | --- | 4.67945 | 4.14675 | 0.532698 | 1.44663 | 0.0145824 | 0.191747 |
| --- | --- | --- | 6.35011 | 5.85813 | 0.491973 | 1.40637 | 0.0159819 | 0.195587 |
| ZMAT3 | chr3q26.32 | zinc finger, matrin-type 3 | 4.87503 | 4.34632 | 0.528716 | 1.44264 | 0.0144304 | 0.191711 |
| NUP43 | chr6q25.1 | nucleoporin 43kDa | 4.46943 | 3.89328 | 0.576154 | 1.49087 | 0.0081303 | 0.169196 |
| ATM | chr11q22-q23 | ATM serine/threonine kinase | 6.93647 | 6.38822 | 0.548254 | 1.46231 | 0.0125881 | 0.187104 |
| TRMT2B | chrXq22.1 | tRNA methyltransferase 2 homolog B | 5.97472 | 5.39515 | 0.579567 | 1.4944 | 0.0042651 | 0.152719 |
| CCND3 | chr6p21 | cyclin D3 | 6.2626 | 5.64806 | 0.614545 | 1.53107 | 0.0121004 | 0.186382 |
| --- | --- | --- | 6.69636 | 6.04441 | 0.651948 | 1.57129 | 0.0078242 | 0.168472 |
| --- | --- | --- | 7.76125 | 7.10689 | 0.654359 | 1.57392 | 0.006192 | 0.161803 |
| --- | --- | --- | 5.60271 | 4.92123 | 0.681479 | 1.60378 | 0.0116608 | 0.185171 |
| POLR2J2  /// POLR2J3 | chr7q22.1 | polymerase (RNA) II (DNA directed) polypeptide J2 /// polymerase (RNA) II (DNA directed) polypeptide J3 | 8.90322 | 8.22884 | 0.674381 | 1.59591 | 0.0078239 | 0.168472 |

| LOC105379  744 /// LOC441259  /// PMS2P5  /// POLR2J2  ///  POLR2J3 | chr7q11.23 /// chr7q22.1 | uncharacterized LOC105379744 /// PMS1 homolog 2, mismatch repair system component pseudogene /// PMS1 homolog 2, mismatch repair system component pseudogene 5 /// polymerase (RNA) II (DNA directed) polypeptide J2 /// polymerase (RNA) II  (DNA directed) polypeptide J3 | 8.30074 | 7.58488 | 0.715864 | 1.64247 | 0.0079509 | 0.168472 |
| --- | --- | --- | --- | --- | --- | --- | --- | --- |
| --- | --- | --- | 4.5837 | 4.01582 | 0.567876 | 1.48234 | 0.0129028 | 0.187472 |
| ZNF224 | chr19q13.2 | zinc finger protein 224 | 5.73865 | 5.32379 | 0.414857 | 1.33317 | 0.0160018 | 0.195587 |
| --- | --- | --- | 5.75716 | 5.31678 | 0.440378 | 1.35696 | 0.0042954 | 0.152719 |
| --- | --- | --- | 6.14012 | 5.66533 | 0.474788 | 1.38971 | 0.0076133 | 0.168472 |
| DCAF8 | chr1q22-q23 | DDB1 and CUL4 associated factor 8 | 4.82543 | 4.31058 | 0.514853 | 1.42885 | 0.0094725 | 0.173123 |
| --- | --- | --- | 6.84511 | 6.28043 | 0.56468 | 1.47906 | 0.0018665 | 0.14618 |
| --- | --- | --- | 6.28808 | 5.74101 | 0.547076 | 1.46112 | 0.0025983 | 0.148407 |
| MDM4 | chr1q32 | MDM4, p53 regulator | 7.01961 | 6.45503 | 0.564573 | 1.47895 | 0.0051305 | 0.157506 |
| CFDP1 | chr16q22.2-  q22.3 | craniofacial development protein 1 | 5.60639 | 4.98675 | 0.619642 | 1.53649 | 0.0038471 | 0.150786 |
| --- | --- | --- | 7.42676 | 6.76903 | 0.65773 | 1.5776 | 0.0023362 | 0.148407 |
| --- | --- | zinc finger protein 146 | 6.15391 | 5.52716 | 0.626753 | 1.54409 | 0.0030438 | 0.148407 |
| --- | --- | --- | 6.67204 | 6.01185 | 0.660195 | 1.5803 | 0.0071433 | 0.166892 |
| --- | --- | --- | 4.63798 | 4.00636 | 0.631621 | 1.5493 | 0.0043642 | 0.152986 |
| LOC100129  917 | chr4p16.3 | uncharacterized LOC100129917 | 4.45911 | 3.78516 | 0.673958 | 1.59544 | 0.002918 | 0.148407 |
| SNRPN | chr15q11.2 | small nuclear ribonucleoprotein  polypeptide N | 6.9538 | 6.2202 | 0.733599 | 1.66278 | 0.0004367 | 0.112391 |
| FTX | chrXq13.2 | FTX transcript, XIST regulator (non-  protein coding) | 6.93201 | 6.29547 | 0.636548 | 1.5546 | 0.0166469 | 0.196814 |
| --- | --- | --- | 7.14587 | 6.29368 | 0.852184 | 1.80523 | 0.0037515 | 0.150735 |
| --- | --- | --- | 7.78848 | 7.02366 | 0.764819 | 1.69916 | 0.0175212 | 0.198559 |
| LOC100190  986 | chr16p12.2 | uncharacterized LOC100190986 | 8.76264 | 7.95455 | 0.808087 | 1.75089 | 0.0091409 | 0.172989 |
| LOC100190  986 | chr16p12.2 | uncharacterized LOC100190986 | 8.12694 | 7.14802 | 0.978921 | 1.97099 | 0.0082014 | 0.169323 |

Supplemental Table S3: Ingenuity Pathway Analysis List of Upstream Regulators Predicted to be Inhibited by Lp299v *Supplementation*

| **Upstream Regulator** | **Expr Log Ratio** | **Molecule Type** | **Predicted Activation State** | **Activation z-score** | **p-value of overlap** | **Target Molecules in Dataset** |
| --- | --- | --- | --- | --- | --- | --- |
| poly rI:rC-RNA |  | biologic drug | Inhibited | -6.057 | 4.71E-12 | A2M,CCND3,CTTN,IFNB1,JAK2,LMO2,LY  96,SOD2,TNFSF10 |
| stallimycin |  | biologic drug | Inhibited | -3.576 | 4.31E-07 | ALAD,BCL2L11,FOXO3,KLF13,MITF,S100  A4,ZNF274 |
| pegintron |  | biologic drug | Inhibited | -2 | 7.57E-06 | AMPD3,CCR1,CXCL10,DUSP6,GM2A,IFI  16,IFNB1,IL1RN,ISG15,NID1,PTX3,SERPI NB2,TNFSF10 |
| oblimersen |  | biologic drug | Inhibited | -3 | 0.00162 | CASP1,CCL3,CCL8,CD80,CD86,CXCL11,I  DO1,MSR1 |
| alefacept |  | biologic drug | Inhibited | -2.433 | 0.0166 | CCL8,CD80,CD86,CXCL10,CXCL11,CXCL9  ,IFI27,IFIH1,IFIT1,IFIT2,ISG15,OAS1,OAS 2,PLSCR1,RSAD2,SOCS1,UBE2D1 |
| 2'3'-cyclic  guanosine monophosphate- adenosine |  | chemical - endogenous mammalian | Inhibited | -2.605 | 6.79E-07 | ATP1B1,CCL3,CCRL2,CFB,DNAJB4,FLT1,  GBP1,GLA,IDO1,IFIT2,INHBA,ISG15,MG LL,PLAU,PTX3,RIN2,SERPINB2,SLAMF7, SYNPO2,TARP |
| tretinoin |  | chemical -  endogenous mammalian | Inhibited | -3.217 | 6.84E-06 | CXCL10,IFI16,IFI27,IFI44,IFIH1,IFIT1,IFIT  2,IFIT3,IFNB1,INHBA,ISG15,OAS1,TNFSF  10 |
| inosine |  | chemical -  endogenous mammalian | Inhibited | -2.795 | 0.00158 | CCL3,CD80,IFNB1,IL1RN,ISG15,OAS1,RS AD2,SOD2,TGFB1,TLR7,TNFSF10 |
| lactic acid |  | chemical - endogenous mammalian | Inhibited | -2.172 | 0.00874 | BCL2L11,CCL24,CCRL2,CD80,CD86,CXCL  10,FGL2,IDO1,IFI16,IFIT2,IFIT3,IL15,MR C1,RSAD2,SERPINB9,SOCS1,TLR7,TNFSF 10,TNFSF13B |
| 5-O-mycolyl-beta- araf-(1->2)-5-O-  mycolyl-alpha- araf-(1->1')- glycerol |  | chemical - endogenous non- mammalian | Inhibited | -3.13 | 9.18E-09 | A2M,ABLIM1,ANXA1,APBB1IP,ATP2A3,  BCL2A1,BHLHE40,BLZF1,C3AR1,C5AR1,  CALM1 (includes others),CAMKK2,CASP1,CASP2,CBFB,CC L24,CCL3,CCND3,CCR1,CCRL2,CD1D,CD 36,CD47,CD6,CD80,CD86,CDC73,CREG1  ,CXCL10,DDX58,DUSP4,EIF4E,ELOC,FAB P5,FGFR1,FLT1,FOXO3,GBP4,GCA,GDF1 5,GM2A,HIVEP3,HOXD4,IFI16,IFI27,IFI4  4,IFIH1,IFIT1,IFIT2,IFIT3,IFIT5,IL15,INHB A,ISG15,JAK2,KLF13,LAMP2,LAP3,LILRB 2,LRRK2,LSP1,LYN,LZTFL1,MDM2,MND A,MRAS,NFATC3,NLN,OAS1,OAS2,OAS3  ,PARP14,PARP9,PDCD4,PIK3R1,PLAU,PL EK,PLSCR1,PPP1R16B,PTAFR,PTX3,RBL1  ,RBM14,RTP4,SAMD9L,SERPINB2,SERPI NB9,SERPINE1,SERPING1,SKP2,SLAMF7  ,SLC5A3,SMAD4,SMURF2,SOCS1,SRGN, SUMO1,TAL1,TFRC,TGFB1,TGFBI,TGFBR 2,TNFAIP6,TNFSF10,TOB1,USP18,VMP1  ,WSB1,YPEL1,YWHAZ,ZEB2 |
| salmonella minnesota R595 lipopolysaccharid es |  | chemical - endogenous non- mammalian | Inhibited | -3.003 | 0.000342 | CCL8,CCRL2,CD80,CD86,CMPK2,CXCL10  ,CXCL9,GDF15,IFIT2,ISG15,LZTFL1,RSAD 2,SERPINB2,SERPINE1,TFEC |

| E. coli lipopolysaccharid e |  | chemical -  endogenous non- mammalian | Inhibited | -2.238 | 0.000918 | IFI44,IFIT1,IFIT2,IFIT3,ISG15,OAS2 |
| --- | --- | --- | --- | --- | --- | --- |
| N-acetylmuramyl- L-alanyl-D- isoglutamine |  | chemical - endogenous non- mammalian | Inhibited | -2.412 | 0.00285 | C3AR1,CCL3,CCND3,CXCR2,DUSP6,FCG R1A,GPR183,IL1RN,LY96,LYZ,PLSCR1,SO CS1,TAL1,TFRC,TGFB1,TLR4,TLR8,TNFSF 10 |
| peptidoglycan |  | chemical - endogenous non- mammalian | Inhibited | -2.076 | 0.011 | ABL1,BHLHE40,CASP1,CCL3,CFB,CLEC7 A,CXCL10,CXCL11,CXCL9,FGF7,GLS,IDO 1,IFNB1,IL1R2,IL1RN,IL2RB,INHBA,KYN U,LYZ,NFATC2,NRG1,PLA2G4A,SERPINB 2,SERPINE1,SOD2,TGFB1,TLR4,TLR7,TN FAIP6,TNFSF10 |
| hemozoin |  | chemical -  endogenous non- mammalian | Inhibited | -2 | 0.0397 | CXCL10,IFI16,IFIH1,IFIT1,IFIT2,IFIT3,IFN B1,RSAD2 |
| cardiotoxin |  | chemical - other | Inhibited | -2.121 | 0.00281 | CASP8,CD80,CD86,CXCL10,CXCL11,CXC R3,DDX58,IDO1,IFI16,IFIH1,IFIT1,IFITM 3,IL15,ISG15,MAVS,OAS2,OAS3,PTAFR, RNASEL,RSAD2,SECTM1,SERPINE1,SOC S1,TFRC,TLR7,TNFSF13B |
| lipopolysaccharid e |  | chemical drug | Inhibited | -5.623 | 6.93E-18 | AIM2,ANXA1,APOBEC3B,BCL2L11,C3AR 1,CCL3,CCL8,CCNG2,CCRL2,CD47,CD80, CD86,CXCL10,CXCL11,CXCL9,FDFT1,FLT 1,GLIPR1,GMPR,HCAR3,IDO1,IFI27,IFIT  1,IFIT2,IFIT3,IFIT5,IFNB1,IL1RN,ISG15,L MO2,MAPK14,MFSD4B,MITF,NAMPT,P HLDB2,PLAU,SECTM1,SERPINB9,SERPIN E1,SERPING1,SOD2,TGFB1,TMEM173,T  NFAIP6,TNFSF10 |
| bromodeoxyuridi ne |  | chemical drug | Inhibited | -2.853 | 5.48E-08 | BCL2A1,CCR1,CD36,CD80,CD86,CXCL10  ,IDO1,IFI27,IFNB1,LYN,MSR1,RASSF4,SE  RPINE1,SOD2,TNFSF10,TNFSF13B |
| ribavirin |  | chemical drug | Inhibited | -3.411 | 1.12E-07 | ANXA1,CCL3,CCL8,CCND3,CD36,CD47,C D80,CD86,CDC14A,CFB,CLEC7A,CXCL10, CXCL11,CXCL9,DDX58,EIF2S3,FCGR1A,G BP1,GDF15,IDO1,IFI16,IFI27,IFIH1,IFIT1, IFIT2,IFIT3,IFIT5,IFITM3,IFNB1,IL15,INH BA,ISG15,JAK2,KMO,KYNU,LYN,LYZ,MR AS,MSR1,NAMPT,NKG7,OAS1,OAS2,OA S3,PLAU,RSAD2,SAT1,SERPING1,SOCS1, SOD2,SRGN,TGFB1,TLR4,TNFAIP6,TNFS  F10,TNFSF13B,TRAFD1 |
| lenalidomide |  | chemical drug | Inhibited | -2.262 | 4.04E-06 | CXCL10,IFI44,IFIT2,IFIT3,IFNB1,ISG15,O  AS1,RSAD2,USP18 |

| resiquimod |  | chemical drug | Inhibited | -2.527 | 7.81E-06 | BCL2A1,CCL3,DUSP6,FYN,IDO1,IFIT2,IL1 RN,INHBA,KMO,MRC1,NFATC2 |
| --- | --- | --- | --- | --- | --- | --- |
| CpG  oligonucleotide |  | chemical drug | Inhibited | -3.091 | 0.000143 | A2M,ANXA1,C1QB,C1QC,CFB,CFD,IFIT  M3,SERPING1 |
| monophosphoryl  lipid A |  | chemical drug | Inhibited | -2.215 | 0.000358 | CCL24,CCL3,CCL8,CXCL10,CXCL9 |
| imiquimod |  | chemical drug | Inhibited | -2.761 | 0.00178 | CCL3,CD80,CD86,CXCL10,IFNB1,ISG15 |
| 3M-001 |  | chemical drug | Inhibited | -2.425 | 0.00908 | CCL8,CCRL2,CD86,CXCL10,CXCL11,CXCL 9,GBP5,IFIH1,IFIT1,IFIT2,ISG15,PIK3AP1  ,PMEPA1,RSAD2,USP18 |
| green tea polyphenol |  | chemical drug | Inhibited | -2.177 | 0.0164 | AIM2,ANKRD10,APOBEC3B,AUTS2,BCL2 A1,C3AR1,CASP1,CASP8,CCL3,CCND3,C CR1,CD1D,CD80,CD86,CHMP5,CXCL10, CXCL11,CXCL9,CXCR3,DDX58,DUSP6,FC ER1A,FCGR1A,GBP1,GLS,GPR180,HERC 5,IDO1,IFI16,IFI27,IFI44,IFIH1,IFIT1,IFIT  2,IFIT3,IFITM3,IFNB1,IL15,IL1RN,IL21R,I SG15,LAP3,MDM2,MNDA,NCR1,NFE2L 3,NKG7,NT5C3A,OAS1,OAS2,OAS3,PAR P14,PARP9,PI4K2B,PLSCR1,PNPT1,RAB GAP1L,RNASEL,RSAD2,RTP4,SAMD9,SA MD9L,SAT1,SERPINB9,SOCS1,STAP1,TD RD7,TGFB1,TLR4,TLR7,TLR8,TNFSF10,T  NFSF13B,TRIOBP,USP18,USP6NL |
| ssRNA40 |  | chemical  reagent | Inhibited | -2.236 | 0.00106 | CCL24,CCL3,CCL8,CD86,CXCL10,CXCL9 |
| 3M-011 |  | chemical reagent | Inhibited | -2.425 | 0.00492 | CCL3,CCL8,CD80,CD86,CXCL10,FGF7,FG FR1,GBP1,GNB4,IL1R2,IL1RN,INHBA,PL A2G4A,PLAU,PTX3,SERPINA1,SERPINE1, SOD2,TGFB1 |
| CpG ODN 1826 |  | chemical  reagent | Inhibited | -2.395 | 0.0354 | CCL8,CXCL10,CXCL11,CXCL9,IFIH1,IFIT1,  ISG15 |
| E. coli B4 lipopolysaccharid e |  | chemical toxicant | Inhibited | -3.866 | 8.13E-10 | ARG2,ATP1B1,BLZF1,CFB,CXCL11,DDX5 8,EMP1,GBP1,GBP5,GFOD1,GLS,HERC5, IFI16,IFI27,IFI44,IFIH1,IFIT1,IFIT2,IFIT3,I FIT5,IFITM3,IL1RN,ISG15,LAP3,LGALS3, MBP,MITF,NKG7,OAS1,OAS2,OAS3,PLA 2G4A,PLAU,PLSCR1,QKI,RCAN3,SECTM  1,TDRD7,TMEM158,TNFSF10,UBE2I,US  P18 |

| Salmonella enterica serotype abortus equi lipopolysaccharid e |  | chemical toxicant | Inhibited | -2.892 | 0.000422 | ABCC1,ACVR2A,AK4,AMPD3,ANKRD22, ANXA1,APOBEC3B,ARG2,ARL4C,ATP2B 1,BCL2A1,BCL2L11,BLZF1,C3AR1,C5AR1  ,CACNA1A,CALM1 (includes others),CARD6,CASP1,CASP8,CCL24,CCL 3,CCL8,CCND3,CCNG2,CCR1,CCRL2,CD1 D,CD36,CD80,CD86,CDC42EP3,CFB,CFD  ,CLEC7A,CMPK2,CREG1,CXCL10,CXCL11  ,CXCL9,CXCR2,CXCR3,CYCS,DDX58,DDX 6,DRAM1,DUSP4,ERN1,ETV7,FABP5,FB XO32,FGL2,FLT1,FYN,GBP1,GBP4,GBP5, GCA,GCLC,GIMAP7,GLCCI1,GLS,GPR183  ,GUCY1B1,HCAR3,HDAC9,HERC5,HESX1  ,IDO1,IDO2,IFI16,IFI27,IFI44,IFIH1,IFIT1, IFIT2,IFIT3,IFIT5,IFNB1,IGSF6,IKZF3,IL15  ,IL1R2,IL1RN,IL21R,IL2RB,INHBA,IRAK3,I RAK4,ISG15,JAK2,KCNAB2,KMO,KYNU,L ACC1,LCP2,LGALS3,LRRFIP1,LRRK2,LY96  ,LYN,LYZ,MARCKS,MRC1,MRTFA,MSR1, NAMPT,NID1,NT5C3A,OAS1,OAS2,OAS 3,PAPSS2,PDCD4,PDK4,PHLDB2,PIK3AP  1,PIK3R1,PLAU,PLEK,PLSCR1,PPP1CB,PP P1R16B,PTAFR,PTX3,RAB20,RAB6A,RSA D2,SAT1,SECTM1,SERPINA1,SERPINB2,S ERPINB9,SERPINE1,SFXN1,SKI,SLAMF7, SLC31A2,SMAD4,SOCS1,SOD2,STAP1,ST EAP4,SUMO1,TCF4,TCF7L2,TFEC,TFPI,T FRC,TGFB1,TLR4,TLR7,TLR8,TNFAIP6,TN FSF10,TNFSF13B,TNFSF14,TRAFD1,TXN,  UBE2I,USP18,UTRN,VHL |
| --- | --- | --- | --- | --- | --- | --- |
| lipid A |  | chemical toxicant | Inhibited | -2.621 | 0.00326 | AMPD3,ANXA1,C1QB,CASP2,CASP8,CD 80,CD86,CXCL10,CXCL9,DDX58,FCGR1A  ,FGL2,GBP4,GLIPR1,IFI16,IFIT3,IFITM3,I FNB1,IL15,IL1RN,IRAK3,KMO,LCP2,LRRF IP1,MRC1,PARP9,PDLIM5,PLA2G4A,PLE K,RAB20,RSAD2,RTP4,SEH1L,SOCS1,SO  D2,TLR4,TNFSF10,USP18 |
| NFkB (complex) |  | complex | Inhibited | -3.494 | 2.34E-05 | CALHM6,CMPK2,CXCL10,DDX58,GBP4,I FI44,IFIH1,IFIT2,IFIT3,ISG15,JAK2,LGALS 3,NUMA1,OAS1,RTP4,SAMD9L,SERPINE  1,SOCS1,TGFBI,TRAFD1,USP18 |
| Fcer1 |  | complex | Inhibited | -2.029 | 9.49E-05 | CCL3,CD80,CD86,CXCL10,CXCR2,IDO1,I FNB1,IRAK3,SOCS1,TNFSF13B |
| Ifn gamma |  | complex | Inhibited | -2.17 | 0.00102 | BCL2A1,C3AR1,CCL3,CCL8,CD86,CIRBP, CXCL10,CXCL11,CXCL9,FBXO32,INHBA,S ERPINE1,TNFSF13B |
| PI3K (complex) |  | complex | Inhibited | -2.173 | 0.00326 | C3AR1,CCL3,CD80,CD86,CXCL10,CXCL1 1,DDX58,HERC5,IDO1,IFI16,IFI27,IFI44,I FIH1,IFIT1,IFIT2,IFIT3,IL15,IL1RN,ISG15, OAS1,OAS2,PNPT1,RSAD2,SOCS1,TGFB 1,TNFSF10,USP18 |

| IgG1 |  | complex | Inhibited | -2 | 0.0156 | AMPD3,CCR1,CXCL10,DUSP6,GBP4,GM 2A,HSP90AA1,IFI16,IFIT2,IFNB1,IL1RN,I SG15,NID1,PLA2G4A,PLAU,PLSCR1,PTX 3,SERPINB2,TNFSF13B |
| --- | --- | --- | --- | --- | --- | --- |
| IFNG | -0.212 | cytokine | Inhibited | -6.505 | 8.18E-19 | CCL8,ISG15,NAMPT,RSAD2 |
| IFNA2 | -0.102 | cytokine | Inhibited | -4.972 | 2.84E-15 | ATM,CASP8,FYN,GDF15,INHBA,MEF2A, MYO1B,N4BP2L2,PLAU,SLC7A5,SMURF 2,STK17A,TGFBR2 |
| IFNL1 | -0.052 | cytokine | Inhibited | -5.039 | 1.38E-14 | ABLIM1,ANGPT1,BCL2L11,CAMKK2,CD3 6,CD6,CXCL10,CXCL9,CXCR3,DUSP4,IL1 R2,IL2RB,MARF1,PDCD4,SOD2,TIAM1,T LE3,TNFRSF18,TNFSF10 |
| IFNB1 | -0.323 | cytokine | Inhibited | -3.67 | 2.31E-13 | CXCL10,IFI16,IFIT2,IFIT3,OAS2,RSAD2,U  SP18 |
| TNF | -0.23 | cytokine | Inhibited | -2.413 | 2.81E-11 | CXCL10,CXCL11,DDX58,IFIH1,ISG15,OA  S1,OAS2 |
| IFNA1/IFNA13 | -0.061 | cytokine | Inhibited | -3.516 | 9.46E-11 | CASP1,CD36,CLEC1A,CMPK2,DDX58,FA R2,GBP1,GCA,GDF15,GIMAP7,GMPR,ID O1,IFNB1,LAP3,MCTP1,NT5C3A,PAPSS2  ,PARP14,PARP9,PLSCR1,PNPT1,RTP4,SA  MD9,SAMD9L,TENT5A,TMEM158,TNFR SF10D,USP18,ZMAT3 |
| IFNA4 | -0.169 | cytokine | Inhibited | -3.198 | 1.13E-10 | DDX58,ISG15,RNF125,USP18 |
| IFNL4 |  | cytokine | Inhibited | -2.623 | 1.13E-07 | CD80,CD86,DDX58,IFNB1,TLR8 |
| PRL | -0.068 | cytokine | Inhibited | -4.799 | 2.07E-07 | CXCL10,DDX58,IFIT1,IFIT2,IFNB1,ISG15,  OAS1 |
| IFNA10 | -0.04 | cytokine | Inhibited | -2.412 | 6.79E-07 | CD80,CD86,CXCL10,CXCL11,CXCL9,EIF4  E,IFIH1,IFIT2,USP18 |

| IFNA5 | 0.003 | cytokine | Inhibited | -2.412 | 6.79E-07 | A2M,AAK1,ABCC1,ALAD,AMPD3,ANGP T1,ANXA1,APOBEC3B,ATP2B1,B4GALT1  ,BCL2A1,BCL2L11,BHLHE40,BLVRA,C3A R1,C5AR1,CARD16,CARD6,CASP1,CASP 2,CASP8,CCL3,CCND3,CCR1,CD36,CD47, CD80,CD86,CFB,CFD,CLASP1,CTDSPL,CT TN,CXCL10,CXCL11,CXCL9,CXCR2,CXCR 3,DBT,DDX58,DUSP4,DUSP6,EMP1,ERN  1,FABP5,FBXO32,FGFR1,FLT1,FYN,GBP1  ,GCLC,GDF15,GLS,GM2A,GNB4,HCAR3, HDAC9,HERC1,HERC5,HSPA4,IDO1,IFI1 6,IFI27,IFIH1,IFIT1,IFIT3,IFIT5,IFNB1,IL1  5,IL1R2,IL1RN,IL21R,INHBA,IRAK3,ISG1  5,KIAA1671,KMO,KYNU,LGALS3,LY96,LY N,MAPK14,MBP,MITF,MSR1,MTHFD2L, MYLK,NAMPT,NFATC2,NID1,NUCB2,OA S1,OAS2,OAS3,OVOS2,PAFAH2,PARP14  ,PLA2G4A,PLAU,PLSCR1,PNPLA8,PPIF,P TX3,QKI,RAB6A,RANBP9,RNASE4,RNF1 25,SAMD9,SAT1,SEC22B,SERPINB2,SER PINB9,SERPINE1,SKI,SLC12A6,SLC1A4,S MURF2,SNRK,SOCS1,SOD2,SQLE,STEAP 4,TBC1D8,TDRD7,TFPI,TFRC,TGFB1,TGF BR2,TGIF1,TLR4,TLR7,TLR8,TNFAIP6,TN FRSF10D,TNFRSF18,TNFSF10,TNFSF13B  ,TNFSF14,TRAFD1,TXN,UBR2,VASH1,V  MP1 |
| --- | --- | --- | --- | --- | --- | --- |
| IFNA7 | 0.04 | cytokine | Inhibited | -2.412 | 6.79E-07 | CCL8,CXCL10,CXCL11,CXCL9,IFIH1,IFIT1,  ISG15 |
| IFNA14 | 0.023 | cytokine | Inhibited | -2.412 | 6.79E-07 | CCL8,CXCL10,CXCL11,CXCL9,IFIH1,IFIT1,  ISG15 |
| IFNA6 | -0.062 | cytokine | Inhibited | -2.412 | 6.79E-07 | CCL8,CXCL10,CXCL11,CXCL9,IFIH1,IFIT1,  ISG15 |
| IFNA21 | -0.098 | cytokine | Inhibited | -2.409 | 6.79E-07 | CCL8,CXCL10,CXCL11,CXCL9,IFIH1,IFIT1,  ISG15 |
| IFNA8 | -0.09 | cytokine | Inhibited | -2.412 | 1.4E-06 | CCL8,CXCL10,CXCL11,CXCL9,IFIH1,IFIT1,  ISG15 |
| IFNA16 | -0.076 | cytokine | Inhibited | -2.412 | 1.4E-06 | CCL8,CXCL10,CXCL11,CXCL9,IFIH1,IFIT1,  ISG15 |
| CD40LG | 0.026 | cytokine | Inhibited | -2.027 | 3.31E-06 | APOBEC3B,ATM,CD80,CD86,CMPK2,CX CL10,CXCL11,FGL2,IFI44,IFIT1,IFIT2,IFIT 3,IFNB1,IL15,INHBA,ISG15,OAS1,OAS2, OAS3,RBL1,RSAD2 |
| IFNE | -0.107 | cytokine | Inhibited | -2.449 | 1.54E-05 | ANXA1,CBFB,CCL3,CCL8,CD86,CMPK2,C XCL10,CXCL11,CXCL9,CXCR3,DDX58,GB P1,GBP4,HERC5,IDO1,IFI16,IFI27,IFI44,I FIH1,IFIT1,IFIT2,IFIT3,IFIT5,IFITM3,IFNB 1,IL2RB,ISG15,LILRB2,LYN,MDM2,OAS1, OAS2,OAS3,PARP9,PLSCR1,PTAFR,RBL1, RSAD2,SAMD9,SERPINB9,SOCS1,TDRD7  ,TNFRSF10D,TNFSF10,USP18 |

| IFNK | 0.08 | cytokine | Inhibited | -2 | 2.06E-05 | BCL2A1,BHLHE40,DDX58,IFI27,IFIT1,IFI T5,IFNB1,ISG15,OAS1,OAS3,PARP9,PLS CR1,SOD2,USP18,ZNF292 |
| --- | --- | --- | --- | --- | --- | --- |
| IL1B | -0.253 | cytokine | Inhibited | -4.452 | 0.000122 | CD80,CD86,FCGR1A,IFNB1,SLC1A4,SLC7  A5 |
| CSF3 | -0.088 | cytokine | Inhibited | -2.496 | 0.00303 | A2M,AIM2,ARG2,ATP1B1,AUTS2,BCL2A 1,BCL2L11,BTN3A1,BTN3A2,C1QB,C1Q C,C5AR1,CALHM6,CARD6,CASP1,CASP2  ,CASP8,CCL3,CCL8,CCND3,CCR1,CCRL2, CD1D,CD36,CD80,CD86,CFB,CIRBP,CLEC 7A,CLIC5,CMPK2,CXCL10,CXCL11,CXCL9  ,CXCR3,DDX58,DTX3L,DUSP4,ETV7,FAB P5,FCGR1A,FCGR1B,FGL2,FLT1,FZD2,GB P1,GBP4,GBP5,GDF15,GLA,GLS,GMPR, GNB4,GNB5,HCAR3,HDAC9,HOXD4,HSP 90AA1,IDO1,IDO2,IFI16,IFI27,IFI44,IFIH  1,IFIT1,IFIT2,IFIT3,IFIT5,IFITM3,IFNB1,IL  15,IL1RN,INHBA,ISG15,JAK2,KMO,KYNU  ,LCP2,LGALS3,LY96,LYN,MAPK14,MDM 2,MITF,MNDA,MRAS,MRC1,MSR1,MTH FD2L,NAMPT,NEURL3,NFE2L3,OAS1,OA S2,OAS3,OPN3,PARP9,PLAU,PLEK,PLSC R1,PSMA4,PTAFR,PTX3,RAB20,RAP2B,R SAD2,RTP4,SAMD9,SERPINA1,SERPINB 9,SERPINE1,SERPING1,SLC7A5,SMAD4, SOCS1,SOD2,SORT1,SPON1,SQLE,TCF7L 2,TFRC,TGFB1,TGFBR2,TGIF1,TLR4,TLR7  ,TLR8,TMEM158,TNFAIP6,TNFSF10,TNF SF13B,TRAFD1,UBE2D1,USP18,USP6NL,  XIST,ZKSCAN1 |
| IL1A | -0.075 | cytokine | Inhibited | -2.699 | 0.00876 | CD80,CD86,CXCL10,IFNB1,IL1RN |
| TNFSF12 | 0.075 | cytokine | Inhibited | -2.372 | 0.0278 | CD80,CD86,CXCL10,CXCL11,CXCL9,IFIH  1,OAS1 |
| CGAS | -0.047 | enzyme | Inhibited | -2.774 | 1.47E-10 | BCL2A1,CCL24,CD86,CXCL10,DLD,FCGR 1A,FCGR1B,GDF15,GM2A,IFNB1,IL15,IN HBA,IRAK3,LAP3,NAMPT,PLAU,PTX3,RA P1A,SOD2,STAP1,TLR8,TNFSF10,TNFSF1 3B |
| DDX58 | -0.448 | enzyme | Inhibited | -2.691 | 2.29E-08 | ANXA1,CASP8,PIK3R1,PLAU,TGFB1 |
| TGM2 | 0.105 | enzyme | Inhibited | -3.966 | 8.03E-07 | CXCL10,DDX58,IFI16,IFI44,IFIT2,IFIT3,IS G15,OAS1,OAS2,OAS3,RSAD2,USP18 |

| IFIH1 | -0.412 | enzyme | Inhibited | -2.38 | 2.07E-05 | BCL2A1,CASP1,CCL3,CCND3,CCNG2,CC R1,CD1D,CD27,CD80,CD86,CXCL10,CXC R3,DUSP4,FLT1,GATB,GPR183,HSP90AA 1,IDO1,IDO2,IFI44,IFIT1,IFIT2,IFIT3,IL15, IL21R,IL2RB,INHBA,ISG15,JAK2,MARCKS  ,NAMPT,PLAU,PLEK,RNF5,SERPINE1,SO CS1,SOD2,TFRC,TGIF1,TNFAIP6,TNFSF1  0 |
| --- | --- | --- | --- | --- | --- | --- |
| PARP9 | -0.34 | enzyme | Inhibited | -2.449 | 0.000223 | A2M,CCND3,CMPK2,CXCL10,CXCL11,CX CL9,DDX58,DTX3L,ELOC,GMPR,HERC5,I FI44,IFIH1,IFIT1,IFIT3,IFIT5,ISG15,JAK2, MICOS10- NBL1/NBL1,OAS1,OAS2,OAS3,PARP14, PDK4,PLSCR1,PNPT1,RNASE4,RSAD2,SA MD9,SAMD9L,SOCS1,TDRD7,TGFB1,TN FSF13B,USP18 |
| RNASE2 | -0.082 | enzyme | Inhibited | -2.394 | 0.00347 | CCL8,CXCL10,CXCL11,CXCL9,IFIH1,IFIT1,  ISG15 |
| RNASE1 | -0.179 | enzyme | Inhibited | -2.213 | 0.00398 | BCL2A1,CD80,CD86,CFD,CYCS,IFIT3,IFN B1,IL1RN,ISG15,PLSCR1,SFXN1,TGFB1,T LR4,TNFSF10,TNFSF13B |
| PLAAT4 | 0.184 | enzyme | Inhibited | -2.173 | 0.0122 | AIM2,APOBEC3B,CASP1,CD86,CXCL10, DDX58,FCER1A,IDO1,IFI16,IFIH1,IFIT2,IF IT3,IFITM3,IFNB1,IL15,ISG15,OAS1,OAS  2,PNPT1,RSAD2,TNFSF10,USP18 |
| NOS2 | -0.052 | enzyme | Inhibited | -2.204 | 0.014 | CXCL10,CXCL11,IFIT1,IFNB1,ISG15 |
| TBK1 | -0.183 | kinase | Inhibited | -3.032 | 1.15E-10 | CXCL10,LY96,MRC1,NCR1,TXN |
| EIF2AK2 | -0.227 | kinase | Inhibited | -2.543 | 2.82E-05 | BCL2L11,CCL3,CCND3,CCNG2,CCR1,CD8 6,CXCL10,FABP5,FBXO32,FRY,GCLC,IFIT  1,IFNB1,IL1RN,LSP1,MITF,MRC1,PDCD4  ,PLA2G4A,RASA3,SERPINE1,SKIL,SLC12 A6,TLR4,TNFSF10 |
| IKBKE | 0.134 | kinase | Inhibited | -2.667 | 0.00424 | CCL3,CD80,CD86,CXCL10,IFNB1,ISG15 |
| CHUK | -0.234 | kinase | Inhibited | -2.648 | 0.00429 | CD80,CD86,CXCL10,IFNB1,LY96,TLR4 |
| IKBKG | -0.046 | kinase | Inhibited | -2.966 | 0.00845 | BCL2A1,CCL3,CCL8,CD80,CD86,CXCL10,I FNB1,IL1RN,SOD2,TLR4,TLR7 |
| mir-15 |  | microRNA | Inhibited | -2.261 | 0.00307 | CCL3,CXCL10,DDX58,IFI27,IFI44,IFIH1,IF IT1,IFIT2,IFIT3,IFNB1,ISG15,OAS1,RSAD 2,SOCS1,TNFSF10 |
| MAVS | 0.31 | other | Inhibited | -3.207 | 3.14E-09 | IFI16,IFI27,IFI44,IFIT1,IFIT3,ISG15,OAS1,  OAS2,RSAD2 |
| TICAM1 | -0.044 | other | Inhibited | -3.66 | 1.26E-08 | A2M,BCL2A1,CCL3,CCND3,CD1D,CD86, CXCL10,CXCR3,DDX58,GBP5,IFI16,IFI44, IFIH1,IFIT1,IFIT2,IFIT3,IFNB1,IL15,IL2RB, ISG15,JAK2,OAS1,OAS2,SOCS1,TXN |

| PAF1 | -0.04 | other | Inhibited | -3.051 | 1.8E-07 | AIM2,CASP1,CD86,CXCL10,IFNB1,ISG15  ,RSAD2 |
| --- | --- | --- | --- | --- | --- | --- |
| DOCK8 | 0.336 | other | Inhibited | -2.138 | 1.61E-06 | CXCL10,DDX58,IDO1,IFI16,IFIH1,IFIT1,IF  IT2,ISG15,TNFSF10 |
| TMEM173 | 0.312 | other | Inhibited | -2.975 | 3.42E-06 | BCL2L11,CCL3,CCL8,CD80,CD86,CXCL10  ,IFNB1,IL15,TGFB1,TLR4 |
| SASH1 | -0.491 | other | Inhibited | -2.138 | 3.72E-06 | CXCL10,DDX58,FBXO32,IFIH1,IFIT1,IFIT 2,IFIT3,IFITM3,ISG15,RSAD2,SOCS1 |
| MYD88 | -0.143 | other | Inhibited | -2.393 | 2.62E-05 | CCL24,CCL3,CCR1,CXCL10,CYCS,FBXO32  ,IL1R2,MEF2A,PTX3 |
| APP | -0.206 | other | Inhibited | -2.93 | 0.00591 | CCL3,CCR1,CD80,CXCR2 |
| HRG | -0.15 | other | Inhibited | -2 | 0.0156 | DDX58,EIF4G2,IFIT1,IFNB1,ISG15,LAMP 1,OAS1,RNF125,SMURF2 |
| IRF7 | -0.173 | transcription regulator | Inhibited | -6.15 | 3.05E-21 | ACSL4,ADGRG3,ANGPT1,ANXA1,ARRB1, ATP6V1C1,BRF1,CARD6,CCL3,CCND3,C DC42EP3,CEP63,CLEC7A,CRTC3,CXCL10, CYSLTR1,DDX58,ETV7,FABP5,FCER1A,F GL2,FLT3LG,GBP1,GBP5,GLIPR1,GPR18 3,HERC5,IFI44,IFIT1,IFIT2,IFIT3,IFIT5,IL1 R2,IL2RB,IRAK3,IRAK4,ISG15,JAK2,LAP3, LGALS2,LGALS3,MAPK14,MEF2A,MPEG 1,MS4A4A,MS4A6A,NDC80,OAS3,PARP  14,PATZ1,PF4V1,RABGAP1L,RSAD2,S10  0A4,SERPING1,SOCS1,SORT1,STK3,TLR4  ,TLR8,TNFRSF25,TRIO,ZBTB20 |
| STAT1 | 0.267 | transcription regulator | Inhibited | -4.007 | 1.1E-17 | CCNG2,CMPK2,CXCL10,DDX58,GBP4,H AVCR2,HIVEP3,IFI16,IFIH1,IFIT2,PARP14  ,PDCD4,RNASEL,RSAD2,RTP4,TLR8,TNF  SF10,USP18 |
| IRF3 | 0.178 | transcription regulator | Inhibited | -5.658 | 5.76E-13 | APOBEC3B,BCL2L11,CCL3,CD80,CD86,C XCL10,EIF1AX,FABP5,IFNB1,IL15,INHBA, ISG15,LGALS2,PLA2G4A,PLSCR1,PTX3,S ERPINE1,SOD2,TCF7L2,TGFB1,TLR4,TNF  RSF18 |
| IRF5 | -0.18 | transcription  regulator | Inhibited | -4.458 | 1.4E-11 | CXCL10,IFI16,IFI44,IFIT2,IFNB1,ISG15,U  SP18 |
| IRF1 | 0.11 | transcription regulator | Inhibited | -3.433 | 1.26E-09 | AKT3,ANKH,ARG2,CALHM6,CD1D,CD36, CFB,CLEC12A,DRAM1,FABP5,GBP5,HTR 7,IFI16,IL1RN,INHBA,MECR,NAMPT,RSA D2,SCIN,SECTM1,SERPINB9,SPON1,TFE C,TGFB1 |
| IRF9 | 0.027 | transcription regulator | Inhibited | -2.745 | 5.25E-08 | AUTS2,BCL11B,C12orf66,C2orf68,CARD 16,CASP1,CCL3,DLD,DLEU2,ERN1,FZD2, IFIH1,NUDCD1,NUP43,PPIF,RASGRP3,R CAN3,RTP4,SEPTIN2,SNRPN,TCF4,TCF7L 2,USP9X,UTRN,WDR4,YWHAZ |

| SPI1 | 0.008 | transcription regulator | Inhibited | -3.592 | 2.81E-06 | AIM2,ARG2,BCL2A1,BCL2L11,CD86,FAR 2,FZD2,GPAT3,IDO1,IFNB1,ME1,MIR15  5HG,MS4A4A,MTMR11,PIK3AP1,PLA2G  4A,RSAD2,SLC25A37,TNFAIP6,TNFRSF1  0D,TNFSF10,TNFSF13B |
| --- | --- | --- | --- | --- | --- | --- |
| NFKBIA | -0.045 | transcription regulator | Inhibited | -2.821 | 1.65E-05 | CXCL10,IFI16,IFI27,IFI44,IFIH1,IFIT1,IFIT 2,IFIT3,IFNB1,INHBA,ISG15,OAS1,SERPI NE1,TGFBI,TNFSF10 |
| NUPR1 | -0.049 | transcription regulator | Inhibited | -2.874 | 0.0397 | APOBEC3B,CD80,CD86,DDX58,HERC5,IF  I27,IFIT2,IFIT3,ISG15,RNF125,RSAD2,US P18 |
| TLR3 | 0.018 | transmembran e receptor | Inhibited | -3.502 | 2.32E-12 | ACSL4,AK4,AKT3,BHLHE40,CALD1,CBFB, CD7,CD86,EIF4G2,GLIPR1,INTS14,JAM3, KLF13,MYLK,OAS3,OPN3,PDP2,PICALM, PPIF,PTAFR,RAP2B,SOS2,TMX1,TP53BP 1,ZHX1 |
| TLR9 | 0.033 | transmembran e receptor | Inhibited | -3.478 | 2.34E-09 | ARRB1,ATM,CXCL10,CXCL11,HDAC2,IFI 27,IFIH1,IFIT1,IFIT3,IFITM3,LY96,MAP3 K3,NFATC2,OAS1,OAS3,PDGFC,PLSCR1, PTAFR,SERPINA1,TSC2 |
| FAS | -0.074 | transmembran e receptor | Inhibited | -2.913 | 2.36E-09 | C5AR1,CASP1,CCND3,CCNE2,CCR1,CD1 80,FGFR1,GM2A,HSP90AA1,IL1RN,LRRF IP1,LTBP1,MRC1,RBL1,SFXN1,TNFRSF18 |
| TLR4 | -0.75 | transmembran  e receptor | Inhibited | -2.506 | 1E-07 | CD80,CD86,CTTN,FCER1A |
| TLR7 | -0.395 | transmembran  e receptor | Inhibited | -3.203 | 1.74E-07 | AUTS2,CXCL9,DLG5,IL2RB,NCR1,SOCS1,  TGFB1 |
| LTBR | -0.093 | transmembran e receptor | Inhibited | -2.449 | 0.0153 | A2M,AMPD3,ANGPT1,ANKH,ANXA1,BC L2A1,CCL24,CCL3,CCR1,CCRL2,CD80,CD 86,CFB,CMPK2,CXCL10,CXCL11,CXCL9,C YSLTR1,EIF4E,FABP5,FGF7,FLT1,GBP1,G CLC,GDF15,GLA,GM2A,HERC5,IDO1,IFI1 6,IFIT1,IFIT3,IL15,IL1R2,IL1RN,INHBA,IR AK3,ISG15,LY96,MAPK14,MYLK,NAMPT  ,NFATC2,OAS2,PLA2G4A,PLAU,PLSCR1, PTX3,RSAD2,SERPINB2,SERPINB9,SERPI NE1,SOCS1,SOD2,SRGN,TGFB1,TGFBR2, TLR4,TLR7,TLR8,TNFAIP6,TNFSF10,TNFS  F13B,TOB1,TRAFD1,USP18 |
| TLR2 | -0.058 | transmembran e receptor | Inhibited | -2.436 | 0.0182 | BHLHE40,CARD6,CASP1,CASP8,CCL3,CD 80,CD86,CMPK2,CXCL10,CXCL11,CXCL9, DDX58,GBP4,GBP5,GNB4,HERC5,IDO1,I FI16,IFI27,IFIH1,IFIT1,IFIT2,IFIT3,IFNB1,I RAK4,ISG15,NT5C3A,OAS1,OAS2,PDK4, PHLDB2,RNASE4,RNASEL,RSAD2,SERPI NB2,SH2D1B,SOCS1,SQLE,TLR7,TLR8,TN  FSF10,USP18,XIST |

**Supplemental Table S4: Ingenuity Pathway Analysis List of Upstream Regulators Predicted to be Activated by Lp299v**

***Supplementation***

| **Upstream Regulator** | **Expr Log Ratio** | **Molecule Type** | **Predicted Activation State** | **Activation z- score** | **p-value of overlap** | **Target Molecules in Dataset** |
| --- | --- | --- | --- | --- | --- | --- |
| IL10RA | -0.034 | transmembrane  receptor | Activated | 2.858 | 0.00645 | CXCL10,GBP1,IFI27,IFIT1,IFIT2,IFIT3,IFITM3,IFNB1,ISG15,OAS2,SOCS1,T  NFSF10 |
| SP110 | -0.137 | transcription regulator | Activated | 3.578 | 0.0000101 | APOBEC3B,ATM,CASP8,CCL3,CCL8,CD80,CD86,CFB,CMPK2,CXCL10,CX CR2,FBXO32,FGL2,FLT3LG,IFI16,IFIT2,IFIT3,IFITM3,IFNB1,IL15,IL2RB,IN HBA,IRAK3,IRAK4,ISG15,LRRK2,LYST,LZTFL1,ORMDL1,ORMDL3,PLEK,PT X3,RBL1,RSAD2,SOCS1,SOD2,TCF4,TFRC,TGFB1,TLR4,TNFSF10 |
| TRIM24 | -0.154 | transcription regulator | Activated | 2.728 | 0.000000003 | CASP1,CASP2,CASP8,CFB,CMPK2,CXCL10,CXCL11,DDX58,FGL2,IDO1,IFI 27,IFIH1,IFIT1,IFIT2,IFIT3,IFIT5,IFITM3,IFNB1,IL15,ISG15,JAK2,OAS1,OA S2,POLH,RSAD2,SOCS1,TGFB1,TNFSF10,TNFSF13B |
| CLOCK | -0.157 | transcription regulator | Activated | 2.138 | 0.00125 | APOBEC3B,ATM,CCRL2,CD80,CD86,CMPK2,CXCL10,FANCF,FBXO32,GD F15,IFIT2,IFNB1,IL15,IL1RN,INHBA,ISG15,LZTFL1,MRC1,MSR1,PLA2G4A  ,RSAD2,SERPINE1,SOCS1,TFEC,TGFB1,TNFSF13B,USP18 |
| NKX2-3 | 0.029 | transcription  regulator | Activated | 2.064 | 0.00000123 | CXCL10,GBP5,IFI16,IFI44,IFIT3,IFITM3,IFNB1,ISG15,OAS1,RSAD2,USP18 |
| PRDM1 | 0.025 | transcription  regulator | Activated | 2 | 0.00143 | CMPK2,CXCL10,DDX58,IFIT1,IFIT2,IFIT3,IFITM3,IFNB1,ISG15,NT5C3A,O  AS1,OAS2,RSAD2,SOCS1,TLR7 |
| TRIM28 | 0.031 | transcription  regulator | Activated | 2 | 0.0202 | CCL3,DDX58,HERC5,IDO1,IFI44,IFIT3,IFITM3,ISG15,OAS2,OAS3,SERPINE  1,SOD2,TENT5A |
| SOCS1 | 0.473 | other | Activated | 2.75 | 1.3E-10 | ABHD15,ALDH5A1,APOBEC3B,CASP2,DLG1,FOXO3,GDF15,GPCPD1,GTF 3C2,H3C1,HIVEP3,IFIT2,IPP,MGLL,NAPEPLD,NUP50,OSGEPL1,PARP9,PI K3R1,POLH,RAB20,RBM14,SAT1,SERPINE1,SIGMAR1,SKP2,SPG7,SPTSS A,SRSF1,TCAF2,TEP1,TMEM158,TOB1,TP53BP1,TRAFD1 |
| Irgm1 |  | other | Activated | 2.646 | 0.00279 | C1QC,C3AR1,CCL3,CCNE2,CD180,CD1D,CMPK2,CXCL10,DUSP6,FCER1A  ,IFI27,IFI44,IFIT1,IFIT2,IFIT3,IFITM3,IL1R2,IL1RN,IRAK3,ISG15,KLF13,LM O2,LYZ,MBP,MRC1,RSAD2,TFEC,TLR4,TNFSF10,USP18 |
| MAPK1 | 0.177 | kinase | Activated | 3.629 | 0.00000013 | CD80,CD86,IFNB1,LYZ,SERPINA1,TNFSF13B |
| ACKR2 | -0.154 | G-protein coupled receptor | Activated | 3.464 | 5.25E-08 | BCL2A1,CASP8,CCL3,CCR1,CCRL2,CD1D,CD80,CD86,CLASP1,CXCL10,CX CL11,CXCL9,CXCR2,CXCR3,FGL2,FLT1,FOXO3,GBP1,GLIPR1,GRK5,HCAR 3,IFIT1,LGALS3,LILRA1,LILRB2,NCR1,NUMA1,PDLIM7,PLAU,PTX3,RSAD  2,SERPINB2,SERPINB9,SERPINE1,SKIL,SOD2,SORL1,SPG7,SPTBN1,STK17  A,STK3,TFRC,TGFB1,TLR8,TNFAIP6,TNFSF10,TP53BP1 |
| PTGER4 | 0.43 | G-protein coupled receptor | Activated | 2.868 | 0.000103 | AIM2,AKT3,ARG2,CCL3,CD80,CD86,CFB,CMPK2,CSRP3,CXCL10,CXCL11, DDX58,FLT3LG,GBP4,HERC5,IFI16,IFI44,IFIH1,IFIT1,IFIT2,IFIT3,IFNB1,IL1 5,IL1R2,IL1RN,ISG15,NFE2L3,OAS1,PIK3R1,PTX3,RSAD2,SERPINB9,SERP INE1,SOCS1,TLR4,TLR7,TNFSF10,TNFSF13B,USP18 |
| TAB1 | 0.037 | enzyme | Activated | 2.804 | 0.0000377 | A2M,AMPD3,BCL2A1,CASP8,CCNE2,CCR1,CD80,CD86,CKS1B,CXCL10,C XCL11,DUSP6,EIF4G2,GM2A,GRK5,IFI16,IFNB1,IL15,IL1RN,ISG15,MDM 2,NID1,PICALM,PIK3R1,PLAU,PMEPA1,PTX3,RASA3,SAT1,SERPINB2,SM AD4,SOD2,SORL1,SPTBN1,TCEAL9,TFRC,TGFB1,TLR4,TNFSF10,TNFSF14  ,USP9X,YWHAZ |
| DNASE2 | 0.327 | enzyme | Activated | 2.59 | 0.000000006 | CCL3,CMPK2,CXCL10,CXCL11,DDX58,IFI44,IFIH1,IFIT1,IFIT2,IFIT3,IFITM 3,IFNB1,ISG15,NAMPT,NT5C3A,OAS1,OAS2,PLSCR1,RSAD2,TGFB1,TNFS  F10 |
| IDO1 | -0.559 | enzyme | Activated | 2.433 | 0.000223 | APOBEC3B,ARG2,CALHM6,CCL3,CCRL2,CD86,CMPK2,CXCL10,DDX58,F CGR1A,GBP1,GBP5,IFI16,IFI27,IFI44,IFIH1,IFIT1,IFIT2,IFIT3,IFITM3,IFNB 1,IL15,ISG15,NT5C3A,OAS1,OAS2,OAS3,PARP14,RSAD2,SAMD9L,SORL1  ,TDRD7,TLR4,TNFSF10,USP18 |
| TREX1 | -0.117 | enzyme | Activated | 2.207 | 3.55E-08 | C1QB,C1QC,CCL24,FCGR1A |
| IL1RN | -0.671 | cytokine | Activated | 4.022 | 0.00000119 | CCR1,CD80,CD86,CXCL10,IFNB1,IL15,IL1RN,IRAK3,IRAK4,PTX3,SOCS1,T  GFB1,TLR4 |
| 2-aminopurine |  | chemical reagent | Activated | 2.609 | 0.0000656 | A2M,APOBEC3B,BCL2L11,CALHM6,CASP1,CASP2,CASP8,CCL3,CCND3,C CRL2,CD86,CFB,CLIC5,CMPK2,CXCL10,CXCL11,CXCL9,CXCR3,FCGR1A,F GF7,FGL2,GBP1,GBP4,GBP5,HAVCR2,IDO1,IFI16,IFI27,IFIH1,IFIT1,IFIT2,I FIT3,IFITM3,IFNB1,IL15,ISG15,JAK2,LY96,MDM2,NEURL3,OAS1,OAS2,P ARP9,PDGFC,RSAD2,RTP4,SAMD9L,SERPING1,SOCS1,SORT1,TLR4,TLR8,  TNFSF10,TNFSF13B,TRAFD1,USP18,ZNF652 |

| 5-azacytidine |  | chemical drug | Activated | 2.142 | 0.0298 | ATM,CMPK2,CXCL10,FGL2,IFIT2,IFIT3,IFNB1,IL15,INHBA,ISG15,RBL1,RS  AD2,SOCS1,TCF4 |
| --- | --- | --- | --- | --- | --- | --- |
| tacrolimus |  | chemical drug | Activated | 2.065 | 0.0242 | ABL1,ATM,ATP1B1,BCL2L11,CCL3,CCNG2,CD86,CDC14A,CMPK2,CTTN, CXCL10,CXCR2,CYCS,DDX58,EVL,FCGR1A,FGFR1,FOXO3,FYN,GBP4,GDF 15,HAVCR2,HIVEP3,HSP90AA1,IDO1,IFI16,IFIH1,IFIT2,INHBA,KYNU,LGA LS3,MBP,MRC1,MSR1,NAMPT,PARP14,PDCD4,PIK3R1,PLAU,RBL1,RNA SEL,RSAD2,RTP4,SERPINE1,SIGMAR1,SMARCA2,SOCS1,SOD2,TGFB1,TG  FBR2,TLR4,TLR7,TLR8,TNFSF10,TUBB2A,TXN,USP18,YWHAZ |
| SB203580 |  | chemical - kinase inhibitor | Activated | 2.914 | 0.000000201 | ARG2,CCL3,CD27,CD80,CD86,CXCL10,CXCL9,FLT3LG,IDO1,IFI16,IFIT1,IF IT2,IFIT3,IFNB1,IL21R,IRAK4,ISG15,LAMP2,LYST,NAMPT,OAS2,OAS3,OR MDL1,RSAD2,SERPINE1,SOCS1,TGFB1,TLR4,TLR7,TNFSF10,TNFSF13B,U  SP18 |
| SP600125 |  | chemical - kinase inhibitor | Activated | 2.413 | 0.0191 | CALHM6,CARD16,CCL8,CCRL2,CD80,CMPK2,CXCL10,CXCL9,DDX58,FCG R1A,GBP1,GBP4,GBP5,HERC5,IDO1,IFI16,IFI44,IFIH1,IFIT1,IFIT2,IFIT3,IFI TM3,IFNB1,IL15,ISG15,JAK2,NAMPT,NT5C3A,OAS1,OAS2,OAS3,PARP14  ,PLSCR1,RSAD2,RTP4,SAMD9L,SOCS1,TDRD7,TLR4,TLR8,TNFSF10,TNFS  F13B,USP18 |
| AG490 |  | chemical - kinase  inhibitor | Activated | 2.155 | 0.0319 | ATM,CMPK2,CXCL10,FGL2,IFIT2,IFIT3,IFNB1,IL15,INHBA,ISG15,RBL1,RS  AD2,SOCS1,TCF4 |
| filgrastim |  | biologic drug | Activated | 2.955 | 2.52E-13 | BCL2A1,CCL3,CD1D,CD80,CD86,CXCL10,CXCL9,DMXL2,IDO1,IFIT1,IFIT3  ,IFNB1,IL21R,IRAK4,ISG15,OAS2,OAS3,PLAU,PTX3,RSAD2,SOCS1,SOD2, TLR7 |
| fontolizumab |  | biologic drug | Activated | 2 | 0.0104 | APOBEC3B,CCRL2,CD80,CD86,CFB,CMPK2,CXCL10,CXCL11,DDX58,GDF 15,IFI16,IFIT1,IFIT2,IFIT3,IFNB1,IL15,ISG15,LZTFL1,RSAD2,SERPINE1,SO  CS1,TFEC,TNFSF10 |

Supplemental Table S5: Correlations Between Changes in Gene Expression and Changes in Brachial Artery Flow- Mediated Dilation

| **Gene symbol** | **Pearson's R** | **P-Value** |
| --- | --- | --- |
| ZNF790-AS1 | -0.149969955 | 0.593705 |
| ZNF777 | 0.061201184 | 0.828466 |
| ZNF652 | -0.323136005 | 0.240083 |
| ZNF652 | 0.32645185 | 0.235014 |
| ZNF549 | -0.158109624 | 0.573577 |
| ZNF514 | -0.113789639 | 0.686368 |
| ZNF451 | -0.241305399 | 0.38627 |
| ZNF397 | -0.017609817 | 0.950332 |
| ZNF345 | -0.180342896 | 0.520109 |
| ZMIZ1 | -0.486721467 | 0.065782 |
| ZEB2 | -0.447736842 | 0.09421 |
| ZBTB20 | 0.002832447 | 0.992007 |
| ZBTB20 | -0.003484428 | 0.990167 |
| ZBTB20 | 0.146530922 | 0.602294 |
| ZBTB20 | 0.179265421 | 0.522647 |
| ZBTB20 | -0.093213001 | 0.741088 |
| ZBTB18 | -0.061659101 | 0.8272 |
| YIPF5 | 0.517967358 | 0.047948 |
| WFDC21P | -0.337597689 | 0.218469 |
| WBP5 | -0.130719009 | 0.642395 |
| VPS8 | -0.482066349 | 0.0688 |
| VCAN | -0.275558705 | 0.320189 |
| USP6NL | -0.023761944 | 0.933011 |
| UBXN7 | -0.12185567 | 0.665291 |
| UBR2 | -0.119023258 | 0.672667 |
| UBE2D1 | -0.510855127 | 0.05165 |
| UBE2D1 | -0.490088691 | 0.063659 |
| TXN | -0.048212421 | 0.864519 |
| TTC37 | 0.056263889 | 0.842137 |
| TRMT2B | -0.327858417 | 0.232885 |
| TRIQK | -0.127095202 | 0.651721 |
| TRIOBP | -0.250644232 | 0.367573 |
| TNFSF13B | -0.664027603 | 0.00694 |
| TNFSF13B | -0.639955247 | 0.010184 |
| TNFSF10 | -0.76319979 | 0.000932 |
| TNFSF10 | -0.817365804 | 0.000198 |
| TNFSF10 | -0.667209573 | 0.006581 |
| TNFAIP6 | -0.292660314 | 0.289811 |
| TNFAIP6 | -0.465340671 | 0.08047 |
| TMX1 | -0.480904657 | 0.069569 |
| TMEM245 | -0.28273961 | 0.307218 |

| TLR8 | -0.370624668 | 0.173854 |
| --- | --- | --- |
| TLR7 | -0.727035319 | 0.002134 |
| TLR4 | -0.770631201 | 0.000773 |
| TLR4 | -0.600808989 | 0.017855 |
| TLR4 | -0.631978805 | 0.011486 |
| TLE3 | 0.18381019 | 0.51198 |
| TIAM1 | -0.344625279 | 0.208425 |
| THUMPD3-AS1 | -0.362042606 | 0.184818 |
| THUMPD3-AS1 | -0.49905511 | 0.058249 |
| TFPI | 0.023296692 | 0.93432 |
| TFEC | -0.439308331 | 0.101341 |
| TFEC | -0.477726906 | 0.071702 |
| TFEC | -0.369834433 | 0.174846 |
| TDRD7 | -0.744564811 | 0.001452 |
| TCF7L2 | -0.632334484 | 0.011425 |
| TCF7L2 | -0.656538229 | 0.007847 |
| TCF7L2 | -0.457375302 | 0.086498 |
| TCAF2 | -0.001820179 | 0.994863 |
| TBCD | 0.194668926 | 0.486906 |
| SYNJ2BP | -0.132024476 | 0.639047 |
| STK3 | -0.51550687 | 0.049206 |
| SSH2 | -0.003381003 | 0.990459 |
| SSB | -0.250307613 | 0.368238 |
| SRSF3 | -0.063820611 | 0.821231 |
| SPRED1 | -0.371534031 | 0.172718 |
| SOS2 | -0.400095416 | 0.139492 |
| SORT1 | -0.580097387 | 0.023392 |
| SORT1 | -0.578721457 | 0.023802 |
| SOD2 | -0.188152233 | 0.501883 |
| SOCS1 | -0.308923781 | 0.262568 |
| SNRPN | -0.176657397 | 0.528813 |
| SNRPN | -0.524371926 | 0.044786 |
| SNRK | -0.357654962 | 0.190593 |
| SMCR8 | -0.274290434 | 0.322512 |
| SMAD4 | -0.426790897 | 0.112614 |
| SLC5A3 | -0.695598828 | 0.003982 |
| SLC5A3 | -0.664166377 | 0.006924 |
| SLC44A1 | -0.428276473 | 0.111233 |
| SLC44A1 | -0.365319868 | 0.18058 |
| SLC31A2 | -0.550573783 | 0.033433 |
| SLC12A8 | -0.616038542 | 0.014472 |
| SLAMF7 | -0.650331472 | 0.008666 |
| SKIL | -0.155727681 | 0.579438 |
| SKI | 0.409759767 | 0.129306 |
| SIPA1L1 | -0.210560925 | 0.451295 |
| SGOL2 | -0.029752416 | 0.916173 |
| SGOL2 | 0.056408074 | 0.841737 |

| SFSWAP | -0.438531704 | 0.102016 |
| --- | --- | --- |
| SERPING1 | -0.663146758 | 0.007042 |
| SERPINE1 | 0.480681369 | 0.069717 |
| SERPINB2 | -0.503109985 | 0.055916 |
| SERPINA1 | -0.394759485 | 0.145342 |
| SERPINA1 | -0.42004819 | 0.119034 |
| 9-Sep | 0.178576463 | 0.524272 |
| SECTM1 | -0.626448408 | 0.012462 |
| SEC63 | -0.293326802 | 0.288663 |
| SCIN | -0.696896217 | 0.003887 |
| SAT1 | -0.418986406 | 0.120067 |
| SASH1 | -0.592621003 | 0.019908 |
| SASH1 | -0.481788878 | 0.068983 |
| SAMD9L | -0.702139681 | 0.00352 |
| SAMD9L | -0.723376783 | 0.002304 |
| SAMD9 | -0.42786362 | 0.111616 |
| SAMD9 | -0.53097155 | 0.041691 |
| S100A12 | 0.091217596 | 0.74646 |
| RTN1 | -0.317591083 | 0.248709 |
| RSAD2 | -0.732364853 | 0.001904 |
| RNF219 | -0.131469364 | 0.64047 |
| RNF125 | -0.336211606 | 0.220485 |
| RIN2 | -0.693995622 | 0.004103 |
| RGL1 | -0.5451628 | 0.035577 |
| RCAN3 | 0.139253236 | 0.620628 |
| RBM48 | 0.083346248 | 0.767758 |
| RBM47 | -0.444350861 | 0.097031 |
| RASSF4 | -0.113351518 | 0.68752 |
| RANBP9 | -0.072572895 | 0.797158 |
| RAD51AP1 | -0.409063926 | 0.130022 |
| RABL2A /// RABL2B | -0.240379287 | 0.388151 |
| RAB35 | -0.308538267 | 0.263195 |
| RAB30-AS1 | -0.504881551 | 0.054918 |
| RAB20 | -0.486278124 | 0.066065 |
| RAB11FIP4 | 0.154453913 | 0.582582 |
| R3HCC1L | 0.035851837 | 0.899062 |
| PTX3 | -0.378516295 | 0.164157 |
| PTRH2 | 0.187218492 | 0.504047 |
| PPP1R3B | -0.329384601 | 0.230587 |
| PPP1R12A | -0.39837545 | 0.14136 |
| PLSCR1 | -0.582472857 | 0.022698 |
| PLEKHA2 | 0.022613118 | 0.936244 |
| PLAU | -0.466765428 | 0.079425 |
| PLA2G4A | -0.453941322 | 0.089192 |
| PIK3AP1 | -0.668349685 | 0.006456 |
| PIGX | 0.224461217 | 0.421248 |
| PIGC | 0.334111969 | 0.223562 |

| PID1 | -0.458431096 | 0.085681 |
| --- | --- | --- |
| PHC3 | -0.382198747 | 0.159757 |
| PDP2 | -0.45083469 | 0.091681 |
| PDLIM7 | -0.132889713 | 0.636832 |
| PDK4 | -0.426931137 | 0.112484 |
| PDGFC | -0.385313783 | 0.156097 |
| PDE4DIP | -0.38501487 | 0.156446 |
| PCNX | -0.481821242 | 0.068962 |
| PAPSS2 | -0.123502356 | 0.661015 |
| OSBPL1A | -0.76900284 | 0.000805 |
| OSBPL1A | -0.656889682 | 0.007802 |
| ORMDL3 | 0.019823096 | 0.944098 |
| OAS1 | -0.724488281 | 0.002251 |
| NXT2 | -0.172881367 | 0.537798 |
| NUMA1 | -0.092184397 | 0.743856 |
| NPHP3 | -0.19253197 | 0.491794 |
| NMT2 | -0.202054785 | 0.470192 |
| NID1 | -0.256173201 | 0.356741 |
| NGLY1 | -0.037977718 | 0.893107 |
| NFXL1 | -0.747183907 | 0.001367 |
| NFE2L3 | -0.20722529 | 0.45866 |
| NFATC3 | -0.246345901 | 0.376116 |
| NFATC2 | 0.192548077 | 0.491757 |
| NEXN | -0.463580639 | 0.081776 |
| NDRG3 | -0.115927042 | 0.680761 |
| MYO1B | -0.51391468 | 0.050033 |
| MSR1 | -0.521331107 | 0.046267 |
| MSI2 | -0.178089204 | 0.525424 |
| MS4A6A | -0.341176102 | 0.213317 |
| MS4A4A | 0.09200162 | 0.744348 |
| MS4A14 | -0.611690272 | 0.015383 |
| MPEG1 | -0.595587507 | 0.019144 |
| MPEG1 | -0.662272412 | 0.007145 |
| MNDA | -0.591378168 | 0.020235 |
| MITF | -0.173105088 | 0.537263 |
| MITF | -0.401470795 | 0.13801 |
| MIR21 /// VMP1 | -0.546802024 | 0.034917 |
| MIR155HG | -0.419823197 | 0.119252 |
| MGLL | -0.303064133 | 0.272198 |
| MGEA5 | -0.102730885 | 0.715616 |
| METTL12 | -0.25436449 | 0.360265 |
| MEF2A | -0.266362644 | 0.337251 |
| ME1 | -0.192873042 | 0.491012 |
| MDM2 | -0.326985803 | 0.234205 |
| MARCKS | -0.406189595 | 0.133008 |
| 43525 | -0.185998769 | 0.506879 |
| 43525 | -0.067786586 | 0.810303 |

| 43525 | -0.275202635 | 0.32084 |
| --- | --- | --- |
| MAP3K7CL | -0.049619713 | 0.8606 |
| MAP3K2 | -0.425886842 | 0.113461 |
| MALAT1 | 0.173582304 | 0.536125 |
| LY96 | -0.220764597 | 0.429136 |
| LSP1 | 0.320045642 | 0.244867 |
| LRRC37A2 /// LRRC37A3 | -0.496740346 | 0.059612 |
| LRCH3 | -0.065632916 | 0.816233 |
| LONP2 | -0.059673365 | 0.832692 |
| LOC729732 | 0.266993919 | 0.336063 |
| LOC101928143 /// NUMB | -0.568134408 | 0.027137 |
| LOC100129917 | -0.357265664 | 0.191111 |
| LOC100129518 /// SOD2 | -0.638794905 | 0.010366 |
| LIX1L | -0.065017091 | 0.817931 |
| LINC-PINT | -0.012746131 | 0.96404 |
| LINC01128 | 0.209732955 | 0.453118 |
| LINC00158 | -0.291937275 | 0.29106 |
| LILRB2 | -0.258856424 | 0.35155 |
| LGALS2 | -0.270393101 | 0.329711 |
| LAP3 | -0.796050959 | 0.000385 |
| LAMP2 | -0.493518155 | 0.061548 |
| LAMP1 | 0.039526488 | 0.888772 |
| KYNU | -0.397240025 | 0.142602 |
| KMO | -0.308785266 | 0.262793 |
| KMO | -0.295925241 | 0.284213 |
| JAKMIP2 | -0.44885389 | 0.093292 |
| JAK2 | -0.792050565 | 0.000432 |
| JAK2 | -0.774927621 | 0.000691 |
| IZUMO4 | 0.347544436 | 0.204341 |
| IRAK3 | -0.300486261 | 0.276501 |
| IRAK3 | -0.371002515 | 0.173382 |
| INPP5A | 0.395456399 | 0.144569 |
| INHBA | 0.196742218 | 0.482186 |
| IL21R | 0.621363629 | 0.013415 |
| IL1RN | -0.701942766 | 0.003533 |
| IL1R2 | -0.64847423 | 0.008924 |
| IKBIP | -0.055319285 | 0.844757 |
| IGSF6 | -0.435280622 | 0.104878 |
| IGSF6 | -0.457103059 | 0.086709 |
| IFNB1 | -0.497720938 | 0.059032 |
| IFIT3 | -0.733790558 | 0.001846 |
| IFIT2 | -0.55488925 | 0.031793 |
| IFIT2 | -0.704227832 | 0.003381 |
| IFIT1 | -0.562290251 | 0.029123 |
| IFIH1 | -0.794701273 | 0.0004 |
| IFI44 | -0.769434838 | 0.000797 |
| IFI27 | -0.279388833 | 0.313232 |

| IFI16 | -0.47076034 | 0.076545 |
| --- | --- | --- |
| IFI16 | -0.460043848 | 0.084444 |
| IDO2 | -0.675272541 | 0.005736 |
| IDO1 | -0.520603375 | 0.046627 |
| ICE2 | -0.038091364 | 0.892789 |
| HTR2B | -0.379947088 | 0.162438 |
| HNMT | -0.094859665 | 0.736662 |
| HNMT | -0.185782421 | 0.507382 |
| HIST1H3I | -0.35618123 | 0.192559 |
| HIST1H2BC | 0.01934487 | 0.945445 |
| HGSNAT | -0.402142588 | 0.13729 |
| HESX1 | -0.622869447 | 0.013127 |
| HERC5 | -0.764886091 | 0.000894 |
| HDAC2 | 0.013706944 | 0.961331 |
| HCAR3 | -0.371669888 | 0.172549 |
| GRPEL2 | -0.080652748 | 0.775083 |
| GPR132 | 0.261322996 | 0.346814 |
| GOLGA4 | -0.061984141 | 0.826302 |
| GNL3L | -0.382581446 | 0.159305 |
| GNB4 | -0.562559108 | 0.029029 |
| GMPR | -0.662982819 | 0.007062 |
| GLYR1 | 0.114050706 | 0.685683 |
| GLYR1 | -0.327998153 | 0.232674 |
| GLCCI1 | -0.058781898 | 0.835159 |
| GDF15 | -0.235342675 | 0.398469 |
| GCA | -0.159916356 | 0.569148 |
| GBP1 | -0.708919386 | 0.003086 |
| GBP1 | -0.857442514 | 4.39E-05 |
| GBP1 | -0.656455065 | 0.007857 |
| GAS2L3 | -0.454997396 | 0.088357 |
| GALNT10 | 0.146126307 | 0.603307 |
| FZD2 | -0.526075582 | 0.043971 |
| FYN | -0.046839443 | 0.868346 |
| FXYD6 | -0.539898209 | 0.037761 |
| FRY | -0.473067808 | 0.074916 |
| FNTB | -0.483292726 | 0.067995 |
| FKBP1B | -0.282482794 | 0.307677 |
| FGL2 | -0.379007493 | 0.163566 |
| FGL2 | -0.457689525 | 0.086254 |
| FGFR1OP2 | -0.212878625 | 0.446213 |
| FGFR1 | 0.256285138 | 0.356524 |
| FCGR1B | -0.507480336 | 0.053478 |
| FCER1A | 0.019838512 | 0.944055 |
| FAR2 | -0.639122066 | 0.010314 |
| FAR2 | -0.797581848 | 0.000368 |
| FAN1 | -0.08628385 | 0.759791 |
| FAM72A /// FAM72B /// F | 0.008507478 | 0.975994 |

| FAM49B | -0.424841826 | 0.114445 |
| --- | --- | --- |
| FAM46A | -0.661748291 | 0.007207 |
| FAM26F | -0.291150105 | 0.292422 |
| FAM26F | -0.252116983 | 0.36467 |
| FABP5 | -0.462724159 | 0.082416 |
| EXOC4 | -0.132871055 | 0.63688 |
| ETV7 | -0.671413113 | 0.006129 |
| ERN1 | -0.011995108 | 0.966158 |
| EPS8 | -0.563346211 | 0.028756 |
| EPRS | 0.348456094 | 0.203076 |
| EPB41 | 0.022821353 | 0.935658 |
| EMP1 | -0.576815247 | 0.024378 |
| EMP1 | -0.541193009 | 0.037215 |
| EML4 | -0.014793762 | 0.958268 |
| EML4 | 0.095860911 | 0.733975 |
| EML4 | 0.231085068 | 0.407303 |
| DUSP6 | -0.48568999 | 0.066442 |
| DUSP6 | -0.517237861 | 0.048319 |
| DUSP6 | -0.501079767 | 0.057075 |
| DRAM1 | -0.685840335 | 0.004761 |
| DNPH1 | 0.487071086 | 0.065559 |
| DNAJB4 | -0.420545167 | 0.118552 |
| DMXL2 | -0.541548381 | 0.037066 |
| DLG5 | 0.398450374 | 0.141278 |
| DLG1 | -0.265815192 | 0.338282 |
| DLD | -0.416479764 | 0.122531 |
| DHX35 | -0.172490516 | 0.538731 |
| DHRS9 | -0.67950572 | 0.005329 |
| DENND1B | 0.026295288 | 0.925887 |
| DDX58 | -0.64965272 | 0.00876 |
| DCANP1 /// TIFAB | -0.222744156 | 0.424903 |
| CYP1B1 | -0.409486144 | 0.129587 |
| CYB561D2 | -0.198539319 | 0.478113 |
| CXCL11 | -0.457529395 | 0.086378 |
| CXCL11 | -0.49645063 | 0.059784 |
| CXCL10 | -0.606420501 | 0.016544 |
| CSTA | 0.069496167 | 0.805602 |
| CPVL | -0.219720348 | 0.431378 |
| CPNE8 | -0.594425834 | 0.019441 |
| CPED1 | -0.301147422 | 0.275393 |
| COX15 | -0.206243116 | 0.46084 |
| CMPK2 | -0.752170806 | 0.001217 |
| CLN8 | -0.187946736 | 0.502359 |
| CLIC5 | 0.278646347 | 0.314574 |
| CLIC2 | -0.0407496 | 0.885351 |
| CLECL1 | -0.16734648 | 0.551088 |
| CLEC7A | -0.587536728 | 0.021271 |

| CLEC4E | -0.319193179 | 0.246197 |
| --- | --- | --- |
| CLEC4A | -0.39275635 | 0.14758 |
| CLEC4A | -0.31984625 | 0.245178 |
| CLEC1A | -0.280679271 | 0.310908 |
| CLEC12A | -0.381778774 | 0.160255 |
| CLEC10A | 0.242876616 | 0.383089 |
| CLASP2 | -0.041272329 | 0.883889 |
| CHMP5 | -0.148906195 | 0.596356 |
| CFD | -0.312301586 | 0.257112 |
| CFB | -0.500805744 | 0.057233 |
| CEP68 | -0.084105929 | 0.765696 |
| CEP68 | -0.100266838 | 0.722185 |
| CENPK | -0.064039241 | 0.820628 |
| CDC42EP3 | -0.558234405 | 0.030564 |
| CDC14A | -0.111436983 | 0.692558 |
| CD86 | 0.051163308 | 0.856304 |
| CD80 | -0.480684324 | 0.069715 |
| CD6 | 0.204482126 | 0.464761 |
| CD36 | -0.406227204 | 0.132969 |
| CD36 | -0.405055134 | 0.134199 |
| CD36 | -0.527320878 | 0.043382 |
| CD36 | -0.529348229 | 0.042437 |
| CCRL2 | -0.515176402 | 0.049377 |
| CCR1 | -0.457931634 | 0.086067 |
| CCR1 | -0.467203879 | 0.079105 |
| CCL8 | -0.687782209 | 0.004598 |
| CCL24 | -0.48813871 | 0.064882 |
| CCDC90B | -0.183618077 | 0.512429 |
| CCDC18-AS1 | 0.117911928 | 0.675568 |
| CBWD1 /// CBWD2 /// CB | -0.573548039 | 0.02539 |
| CBFA2T2 | 0.088216075 | 0.754563 |
| CBFA2T2 | -0.156555826 | 0.577397 |
| CASP1 | -0.409120755 | 0.129964 |
| CASK | -0.43466928 | 0.105423 |
| CARD6 | -0.609145481 | 0.015935 |
| CARD16 | -0.126594831 | 0.653013 |
| C5AR1 | -0.449610841 | 0.092674 |
| C3AR1 | -0.616357688 | 0.014407 |
| C1QB | -0.191921385 | 0.493195 |
| C1orf174 | 0.294450431 | 0.286734 |
| C1GALT1C1 | -0.470547433 | 0.076696 |
| BZW1 | -0.109432114 | 0.697846 |
| BROX | -0.333366973 | 0.22466 |
| BRF1 | -0.632525306 | 0.011392 |
| BMS1P5 /// LOC10272445 | -0.276020815 | 0.319345 |
| BMP2K | -0.63423846 | 0.011104 |
| BLZF1 | -0.38479091 | 0.156708 |

| BLVRA | -0.593126898 | 0.019776 |
| --- | --- | --- |
| BLVRA | -0.485984437 | 0.066253 |
| BCOR | 0.175470284 | 0.53163 |
| BCL2L11 | 0.264376438 | 0.341002 |
| BCL2A1 | -0.00019997 | 0.999436 |
| AUTS2 | 0.270765534 | 0.329019 |
| ATP9B | 0.11393587 | 0.685984 |
| ATP6V1C1 | -0.162228126 | 0.563502 |
| ATP2A3 | -0.132704779 | 0.637305 |
| ATP1B1 | -0.067416399 | 0.811322 |
| ATM | -0.380723498 | 0.161511 |
| ARMCX1 | -0.429443468 | 0.110156 |
| ARMC9 | -0.36080205 | 0.186439 |
| ARMC9 | -0.595518019 | 0.019162 |
| ARHGEF7 | -0.055931038 | 0.84306 |
| APOBEC3B | -0.215850952 | 0.439737 |
| ANKRD22 | -0.543300994 | 0.036339 |
| ANKH | -0.217708509 | 0.435714 |
| ALG14 | -0.347128891 | 0.204919 |
| ALAD | -0.185313885 | 0.508473 |
| AKAP11 | -0.483472904 | 0.067878 |
| AK4 | -0.152606414 | 0.587154 |
| AIM2 | -0.354633335 | 0.194637 |
| ADGRG3 | 0.13882491 | 0.621714 |
| ADAMDEC1 | -0.112748212 | 0.689106 |
| ACSL4 | -0.533485563 | 0.040555 |
| ACSL4 | -0.297669674 | 0.281248 |
| AATBC | -0.358661438 | 0.189258 |
| --- | -0.496225094 | 0.059919 |
| --- | -0.496040338 | 0.060029 |
| --- | -0.419797458 | 0.119277 |
| --- | -0.259966121 | 0.349415 |
| --- | -0.559018988 | 0.030281 |
| --- | -0.108106606 | 0.70135 |
| --- | -0.118531298 | 0.673951 |
| --- | -0.51717096 | 0.048353 |
| --- | -0.064107699 | 0.820439 |
| --- | -0.708788148 | 0.003094 |
| --- | -0.365074628 | 0.180895 |
| --- | -0.446607842 | 0.095144 |
| --- | -0.49426272 | 0.061097 |
| --- | -0.515488556 | 0.049215 |
| --- | -0.020381511 | 0.942526 |
| --- | -0.450749709 | 0.091749 |
| --- | -0.058118341 | 0.836997 |
| --- | -0.413328798 | 0.125677 |
| --- | -0.447756274 | 0.094194 |

| --- | 0.260118469 | 0.349122 |
| --- | --- | --- |
| --- | -0.106384044 | 0.705912 |
| --- | 0.064597139 | 0.819089 |
| --- | -0.24555226 | 0.377705 |
| --- | 0.052085353 | 0.85374 |
| --- | 0.134917012 | 0.631653 |
| --- | -0.487212815 | 0.065469 |
| --- | -0.260170905 | 0.349022 |
| --- | -0.126525224 | 0.653193 |
| --- | -0.13354727 | 0.635151 |
| --- | -0.271163928 | 0.32828 |
| --- | -0.083646197 | 0.766944 |
| --- | -0.343021946 | 0.21069 |
| --- | -0.284492239 | 0.3041 |
| --- | -0.436368733 | 0.103914 |
| --- | -0.311169268 | 0.258933 |
| --- | -0.68957138 | 0.004451 |
| --- | -0.113764602 | 0.686434 |
| --- | -0.06245482 | 0.825002 |
| --- | -0.428404403 | 0.111115 |
| --- | -0.367286121 | 0.178067 |
| --- | 0.363950757 | 0.182343 |
| --- | -0.14768299 | 0.599411 |
| --- | -0.128851043 | 0.647197 |
| --- | 0.020873061 | 0.941142 |
| --- | 0.100880549 | 0.720547 |
| --- | -0.052327126 | 0.853068 |
| --- | -0.24227049 | 0.384314 |
| --- | -0.42222851 | 0.116931 |
| --- | -0.101673016 | 0.718434 |
| --- | -0.178181856 | 0.525205 |
| --- | 0.059633413 | 0.832802 |
| --- | -0.513586262 | 0.050205 |
| --- | -0.167276973 | 0.551255 |
| --- | -0.10073675 | 0.720931 |
| --- | -0.371934815 | 0.172219 |
| --- | 0.149571339 | 0.594698 |
| --- | -0.198423666 | 0.478374 |
| --- | -0.308945281 | 0.262533 |
| --- | 0.062971513 | 0.823575 |
| --- | -0.152690603 | 0.586946 |
| --- | 0.067744451 | 0.810419 |
| --- | -0.033407284 | 0.905915 |
| --- | 0.159729917 | 0.569604 |
| --- | -0.058261303 | 0.836601 |
| --- | -0.197233195 | 0.481072 |
| --- | 0.072891085 | 0.796285 |

| --- | -0.437401953 | 0.103005 |
| --- | --- | --- |
| --- | -0.080770899 | 0.774761 |
| --- | -0.708108148 | 0.003136 |
| --- | 0.021224747 | 0.940152 |
| --- | -0.172657126 | 0.538333 |
